# Nintedanib inhibits the VEGFR–ERK signaling pathway in human *KRAS*-mutated cancer cells

**DOI:** 10.1038/s41419-026-09015-2

**Published:** 2026-06-23

**Authors:** Sivasundaram Karnan, Akinobu Ota, Muhammad Nazmul Hasan, Toshinori Hyodo, Nushrat Jahan, Hideki Murakami, Md Towhid Ahmed Shihan, Ichiro Hanamura, Lam Quang Vu, Miho Riku, Hideaki Ito, Yoshifumi Kaneko, Yinzhi Lin, Md Wahiduzzaman, Md. Lutfur Rahman, Shingo Inaguma, Takuya Matsui, Hiroyuki Konishi, Shinobu Tsuzuki, Yoshitaka Hosokawa

**Affiliations:** 1 Department of Biochemistry, Aichi Medical University School of Medicine, Nagakute, Japan; 2 Department of Nutritional Environment, College of Human Life and Environment, Kinjo Gakuin University, Nagoya, Japan; 3 EuGEF Research Foundation, Chattogram, Bangladesh; 4 Department of Internal Medicine, University of Texas Southwestern Medical Center, Dallas, TX USA; 5 Department of Pathology, Aichi Medical University School of Medicine, Nagakute, Japan; 6 Division of Hematology, Department of Internal Medicine, Aichi Medical University School of Medicine, Nagakute, Japan; 7 Department of Pathology, University of Michigan, Ann Arbor, MI USA; 8 Department of Foundations of Medicine, NYU Grossman Long Island School of Medicine, Mineola, NY USA; 9 Department of Biochemistry, Emory University School of Medicine, Atlanta, GA USA; 10 Department of Pathology, Nagoya City University East Medical Center, Nagoya, Japan; 11 Department of physiology, Aichi Medical University School of Medicine, Nagakute, Japan

**Keywords:** Drug development, High-throughput screening

## Abstract

*KRAS* mutations are significant drivers in various cancers, and existing drug discovery attempts targeting these mutations have largely been unsuccessful, emphasizing the need for more effective therapies. In this study, the screening library, which contains 1,374 chemical compounds, identified nintedanib, a VEGFR inhibitor, as exerting a potent and selective antiproliferative effect against *KRAS*-mutant cells, surpassing other VEGFR inhibitors. Nintedanib effectively suppressed tumor growth in xenografted mice with *KRAS* mutations and significantly inhibited phosphorylated VEGFR2 levels and its downstream signaling molecules pAKT and pERK in *KRAS*-mutant cells, suggesting that VEGFR2 inhibition affects the oncogenic AKT/ERK pathway. Moreover, in VEGFR2-knockout cells, inhibition of SOS1 protein reduced KRAS-GTP activity, which decreased the phosphorylation of ERK, AKT, and DRP1, thereby inducing apoptosis. Remarkably, *KRAS*^G12D^ overexpression augmented VEGFR2 expression, establishing a positive feedback loop between *KRAS* mutations and VEGFR2 signaling within the ERK pathway. Immunohistochemical analyses of pancreatic cancer tissues revealed high VEGFR2 expression in 83% (67/80) of samples, significantly exceeding the levels observed in normal pancreatic tissues. These results underscore VEGFR2 as a promising molecular target and propose a novel therapeutic avenue for *KRAS*-mutant cancers.

## Introduction

*KRAS* mutations play a vital role in the development and progression of various cancers, particularly pancreatic, colorectal, and lung cancers [1–3]. The high mutation rate of *KRAS* in pancreatic cancer, estimated at 70%–90%, correlates with a poor 5-year survival rate of <5%, emphasizing the urgent need for more effective therapeutic strategies [2, 4, 5]. Although the EGFR kinase inhibitor erlotinib has received approval as a treatment choice, its impact on survival has been limited [5–9]. In colorectal cancer, anti-EGFR monoclonal antibodies have been successfully deployed; however, the presence of RAS mutations severely compromises their efficacy, thereby rendering them ineffective [6, 7, 10, 11]. Similarly, in the context of lung cancer, although erlotinib is commercially available for patients with EGFR mutations, it has been demonstrated to be ineffective in those with *KRAS* mutations [12].

Nevertheless, in recent years, advancements in drug discovery and deeper understanding of KRAS biology have resulted in some breakthroughs. For instance, the development of small molecule inhibitors that specifically target *KRAS*^G12C^ mutations has shown promise in preclinical studies and clinical trials. Drugs such as sotorasib (AMG 510) and adagrasib (MRTX849) have received attention for their ability to selectively inhibit the G12C variant of *KRAS*, resulting in significant clinical responses in patients with tumors harboring this specific mutation [10, 11, 13–17]. Nevertheless, a broader challenge remains because several variants lack a defined binding pocket for effective inhibitor binding. Therefore, research continues to refine our understanding of RAS biology and develop innovative therapeutic strategies that effectively target these persistent oncogenic drivers [18, 19].

Considering the critical role of mutant KRAS in tumorigenesis, there is an urgent need to investigate novel therapeutic avenues. Despite the significance of mutant *KRAS* in the proliferation of cancer cells, there have been significant challenges in the development of direct KRAS inhibitors due to the absence of unique structural characteristics that could serve as drug targets [13, 20]. Furthermore, existing inhibitors targeting downstream signaling or lipid modifications demonstrate limited effectiveness [14, 21]. Based on microarray analysis, we found that the levels of VEGF signaling molecules are increased in *KRAS*-mutant cells. Therefore, a deeper understanding of the regulation of KRAS and its upstream signaling molecules, particularly the vascular endothelial growth factor receptor (VEGFR), is essential. This knowledge could result in the development of new therapies for cancers associated with *KRAS* mutations.

*KRAS* mutations play a vital role in cell signaling pathways that determine cell proliferation and survival [15, 18]. Mutations typically occur in the GTP-binding pocket, particularly at residues 12, 13, and 61 [20–22]. These mutations inhibit RAS GTPase activity, leading to a state of sustained activity that continuously signals downstream pathways without the usual regulatory mechanisms provided by RAS GTPase-activating protein (GAP) [23]. To further investigate the molecular dynamics, it is insightful to refer to the role of the Son of Sevenless family of guanine nucleotide exchange factors that activate RAS by promoting the exchange from the GDP-bound state to the GTP-bound state. [20, 24]. Associated with RAS mutations, hyperactive signaling is further exacerbated by increased SOS activity and contributes to the oncogenicity of mutant RAS proteins [25]. Studies on SOS1 interaction with its mutant RAS proteins have provided valuable insights into potential therapeutic strategies [26]. previous studies have elucidated the molecular mechanisms underlying KRAS-mediated oncogenesis, emphasizing the role of downstream effectors such as the ERK/MAPK and PI3K/AKT pathways [27, 28]. These pathways are crucial mediators of cell proliferation, survival, and differentiation, and their dysregulation is a hallmark of KRAS-driven cancers. Our work demonstrates how specific interventions modulate these signaling cascades, with evidence pointing to alterations in phosphorylated ERK (pERK) and AKT (pAKT) levels, as well as the impact of VEGFR2 knockout (KO) and SOS1–KRAS-GTP activity assays. In particular, in *VEGFR2*-KO/PL8 and *VEGFR2*-KO/HCT116 mutant cells in our study, inhibition of SOS1 reduced KRAS-GTP activity, resulting in decreased phosphorylation of ERK, AKT, and DRP1, and ultimately induced apoptosis.

In our study, we introduced a mutation at the G12 position of *KRAS* in various human somatic cells and myeloma cell lines. This approach aimed to clarify the altered molecular functions resulting from *KRAS* mutations and identify novel therapeutic targets. In addition, we screened a library of pharmacological agents and identified nintedanib, an intracellular tyrosine kinase inhibitor, as a potent inhibitor of cell survival in the context of KRAS mutants. Our results suggest that nintedanib selectively inhibits the proliferation of *KRAS*-mutant cells, warranting further research as a potential candidate for targeted therapy in KRAS-driven malignancies.

## Results

### Establishment of KRAS-mutant human pancreatic cells

**Fig. 1:**
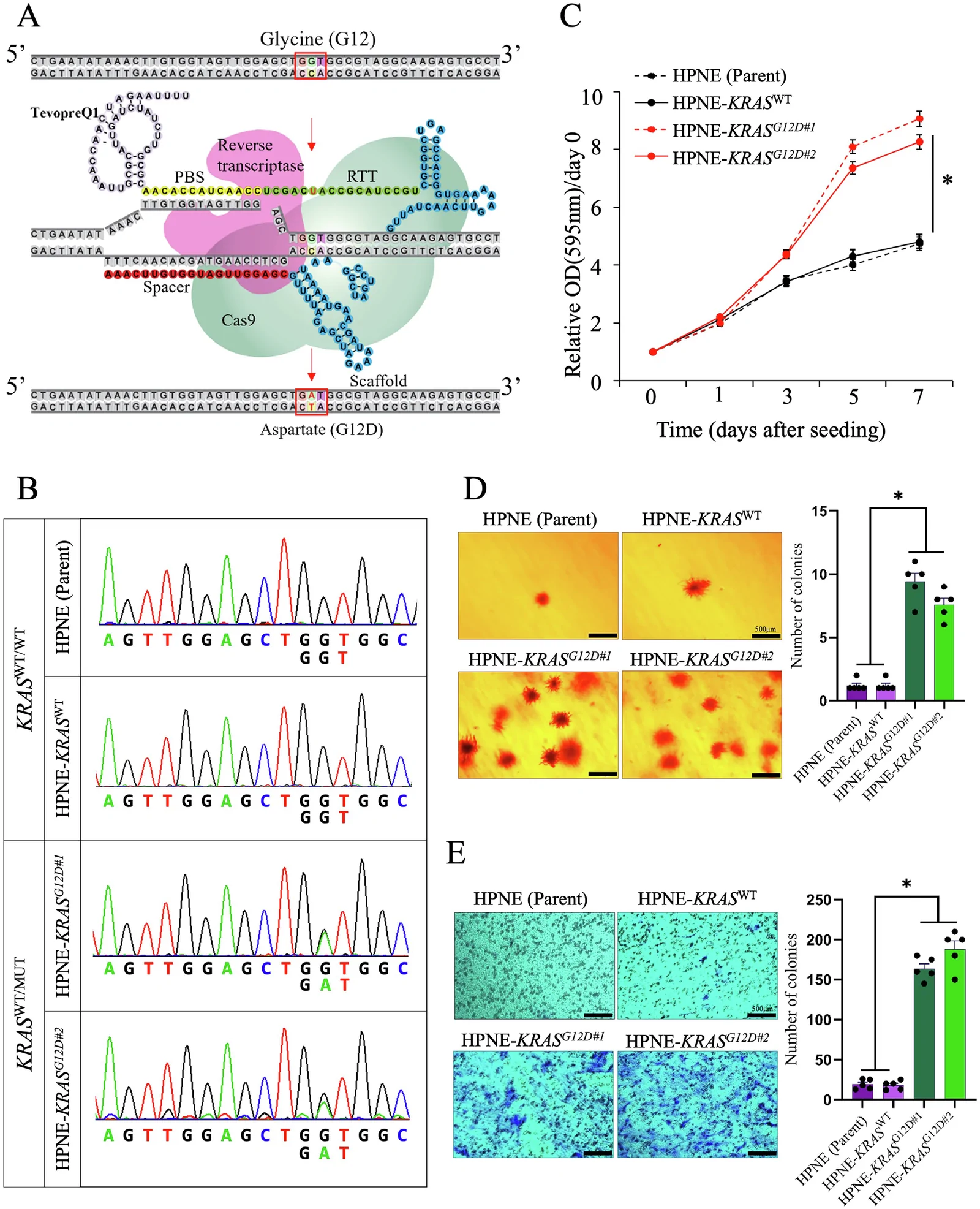
Establishment of KRAS-G12D knock-in cells by prime editing. **A** Schematic of the prime editing mechanism using pegRNA and Cas9 nickase for precise genome editing. The pegRNA comprises a spacer (red) for target recognition, a scaffold (blue) for stability, a reverse transcription template (RTT, light green) for intended edit, a primer-binding site (PBS, yellow) for reverse transcription initiation, and a 3′ tevopreQ_1_structural motif (purple) for increased pegRNA stability. Upon binding to the target genomic DNA (black and gray), Cas9 nickase (green) induces a single-strand break in the opposite strand, creating a nick. The pegRNA subsequently hybridizes to the nicked DNA, enabling the reverse transcriptase (pink) to extend and incorporate the desired sequence from the RTT. The edited strand is depicted in black, with the complementary strand in light gray, demonstrating prime editing. **B** Genomic sequence validation of *KRAS* in HPNE knock-in cell clones compared with that in parental cells. **C** MTT-based proliferation assay of parental HPNE cells, control HPNE-*KRAS*^WT^ cell clone, and two HPNE-*KRAS*^G12D^ cell clones (#1 and #2). The relative OD at 595 nm was calculated by dividing the OD of day 0 at each time point (0, 1, 3, 5, and 7) and is expressed as mean ± SEM (n = 3). **D** Soft agar colony formation assay. A total of 500 cells of each clone were seeded in a 12-well plate and cultured for 14 days, followed by MTT staining and imaging. Representative images (left) and quantified colony numbers (right bar graph) are depicted. Data are expressed as mean ± SEM (n = 5). **E** Boyden chamber migration assay. Cells (2.5 × 10^5^/well) were seeded in transwell inserts placed in 24-well plates and allowed to migrate for 24 h. The migrated cells were stained with crystal violet and imaged. The bar graph (right) illustrates the quantification of migrated cells. Data indicate mean ± SEM (n = 5). (*p < 0.05).

To determine the role of *KRAS* mutations in pancreatic epithelial cells, we generated *KRAS*-mutant HPNE cell clones using the CRISPR-Cas9 prime editing system (PE_max_ + epegRNAtevopreQ_1_) targeting exon 2 of *KRAS* in the hTERT-immortalized HPNE cell lines (Fig. 1A). Two *KRAS*^G12D^ knock-in clones and one *KRAS*^WT^ control clone were successfully isolated (designated HPNE-*KRAS*^G12D #1^, HPNE-*KRAS*^G12D #2^, and HPNE-*KRAS*^WT^). Sanger sequencing confirmed heteroallelic G12D knock-in events in the mutant clones (Fig. 1B).

### KRAS^G12D^ knock-in increases cell proliferation and clonogenicity in HPNE cells

Cell growth analysis using the MTT assay revealed significantly increased proliferation in *KRAS*^G12D^ clones compared with that in the parental HPNE and *KRAS*^WT^ control cells (Fig. 1C). Moreover, *KRAS*^G12D^ clones formed a greater number of colonies in soft agar assays than control cells (Fig. 1D). Transwell migration assays also indicated a marked increase in migratory capacity in *KRAS*^G12D^ cells (Fig. 1E). These results emphasize the oncogenic potential of *KRAS*^G12D^ in driving proliferation, anchorage-independent growth, and migration in HPNE cells*.*

### KRAS mutation induces the expression of KRAS-related molecules

In the cDNA microarray analysis comparing *KRAS*^G12D^ clones with control cells, 95 genes upregulated (>5-fold) and 13 genes downregulated (<0.2-fold) were identified in *KRAS*^G12D^ cells (Fig. S1A, Tables S1, S2). Pathway enrichment using Panther classification analysis highlighted the activation of cell proliferation pathways, such as EGF, FGF, and VEGF signaling (Fig. S1B). Gene set enrichment analysis further confirmed the activation of VEGF_A_UP.V1 and KRAS_DF.V1 oncogenic gene sets in KRAS^G12D^ clones (Fig. S2A, B). Western blotting validated the increased expression of VEGF-A in *KRAS*^G12D^ cells compared with that in KRAS^WT^ cells, implicating *KRAS*^G12D^ in modulating the VEGF–VEGFR axis (Fig. S1C).

### Screening of 1374 pharmacological compounds for selective efficacy in KRAS-mutant cells

**Fig. 2:**
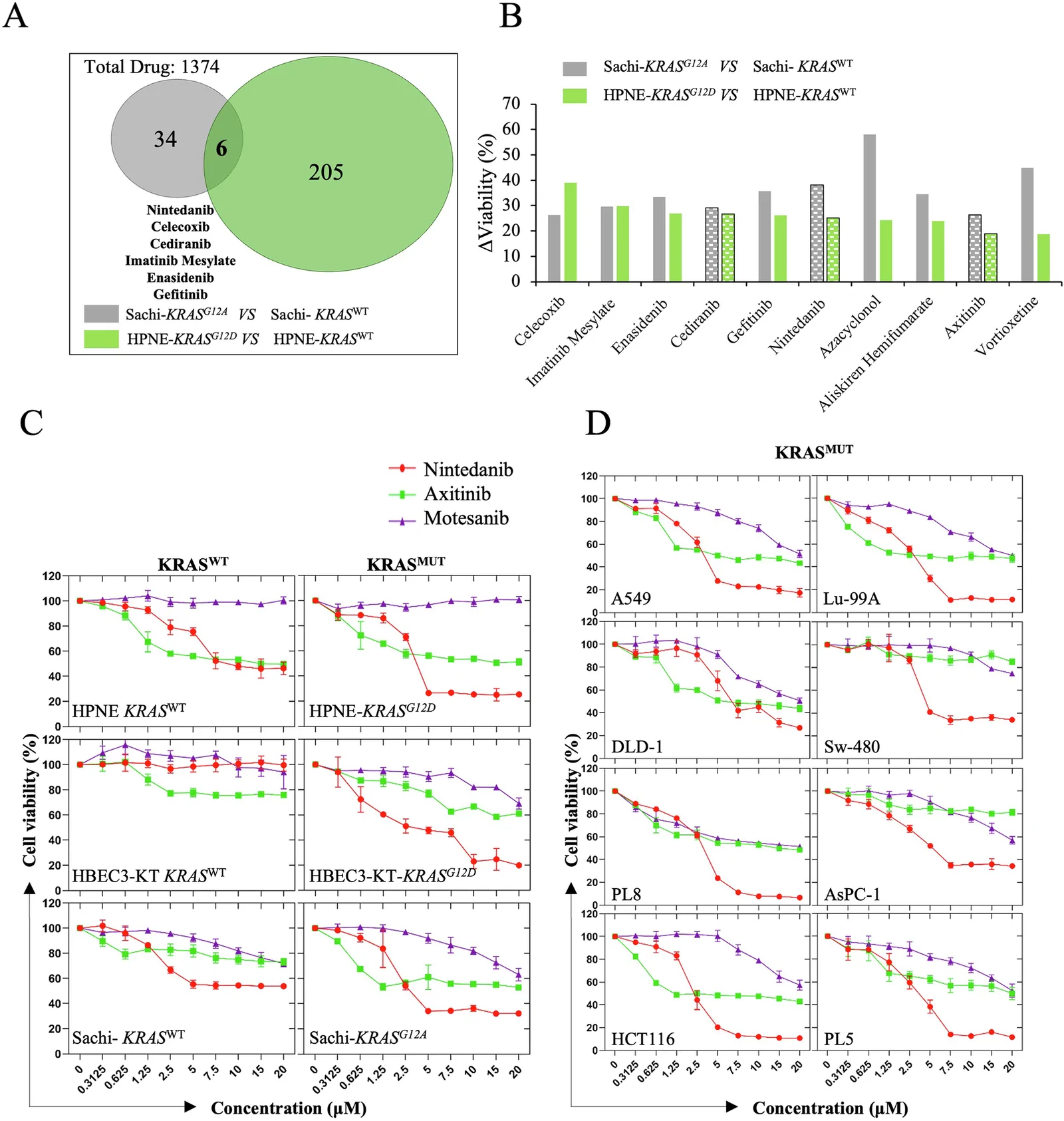
Identification of VEGFR inhibitors targeting KRAS-mutant cells and their effects on cell viability. **A** A total of 1 374 compounds were screened to determine their effects on cell proliferation using *KRAS*-mutant knock-in cell lines. The criterion for selecting active compounds was a reduction in cell viability by ≥25% in mutant cells compared with that in wild-type cells (i.e., Sachi vs. Sachi-KRAS^G12A^ and HPNE vs HPNE-KRAS^G12D^). From this screening, 34 active compounds were identified in Sachi-KRAS^G12A^ cells (gray), and 205 active compounds were identified in HPNE-KRAS^G12D^ cells (green), with 6 compounds commonly active in both *KRAS*-mutant cell lines. **B** Top 10 candidate compounds identified from the screening. Pattern-filled bar representing VEGFR inhibitors among the top hits. **C** Effect of axitinib, motesanib, and nintedanib on cell survival in various KRAS knock-in cell lines compared with that in KRAS wild-type cells (HPNE, HBEC3-KT, and Sachi) and **D** other cancer cell lines (A549, Lu-99A, DLD1, SW-480, HCT116, PL5, PL8, and AsPC-1). Cells were treated with indicated drug concentrations (20, 15, 10, 7.5, 5, 2.5, 1.25, 0.625, and 0 μM) for 72 h. Cell viability was determined using MTT assays (OD595), and survival percentages were calculated accordingly. Nintedanib, axitinib, and motesanib are represented by red, green, and purple lines, respectively. Data are expressed as mean ± SEM (n = 3).

We next conducted a drug-screening assay using the Selleck L2000 library (catalog number Z295656-384), which contains 1374 chemical compounds, including known FDA-approved drugs. The screening was conducted using KRAS-mutant cells (*KRAS*^*G12A*^ Sachi and *KRAS*^*G12D*^ HPNE cell clones) and the corresponding KRAS wild-type controls (Sachi and HPNE cells). Table S3 presents a summary of the results. Of the 1374 compounds tested, 677 (49%) demonstrated antiproliferative activity in both *KRAS*^*G12A*^ Sachi and *KRAS*^*G12D*^ HPNE cells compared with *KRAS*^*WT*^ counterparts. To identify the compounds with selective efficacy in KRAS-mutant cells, we applied a threshold of at least 25% greater reduction in mutant versus wild-type cells. On the basis of this criterion, 34 and 205 compounds were identified in *KRAS*^*G12A*^ Sachi cells and *KRAS*^*G12D*^ HPNE cells, respectively, with 6 compounds (nintedanib, celecoxib, cediranib, imatinib mesylate, enasidenib, and gefitinib) common to both cell lines (Fig. 2A). Remarkably, among the top 10 candidate compounds, 3 (nintedanib, cediranib, and axitinib) targeted the VEGF signaling pathway (Fig. 2B), emphasizing the potential importance of VEGFR inhibition in *KRAS*-mutant contexts. Specifically, in the compound library screening, Nintedanib ranked 9th among 1,374 compounds in the comparison between Sach-KRAS WT and Sach-KRAS mutant cells, producing a 25% reduction in cell viability. In contrast, in the comparison between HPNE-KRAS WT and HPNE-KRAS mutant cells, Nintedanib ranked 203rd. Among both cell lines, when comparing KRAS WT and KRAS mutant cells, Nintedanib was the most effective compound in reducing cell viability by 25% across the cell lines tested.

We subsequently examined all VEGF-targeting inhibitors in the library and identified 21 compounds, of which at least 16 demonstrated minimal antiproliferative activity in both cell lines (Fig. S3). A detailed summary of their effects on cell viability is provided in Table S4.

### Nintedanib exerts the best antiproliferative effect on KRAS-mutant cancer cells

Next, we compared the efficacy of two VEGFR inhibitors identified from our screening, nintedanib and axitinib, and included an additional VEGFR inhibitor, motesanib, for evaluation in *KRAS*-mutant cell lines. The MTT assays and IC_50_ analyses revealed that nintedanib exerted the strongest antiproliferative effect against KRAS-mutant cells among the tested VEGFR inhibitors. Remarkably, nintedanib significantly reduced the cell viability not only in cells with induced *KRAS*^G12D^ and *KRAS*^G12A^ mutations (Sachi, HPNE, and HBEC3-KT) (Fig. 2C) but also in several cancer cell lines harboring spontaneous *KRAS* mutations, such as A549, Lu-99A, DLD-1, Sw-480, PL8, AsPc-1, HCT116, and PL5 (Fig. 2D; Table S5). The mean IC_50_ value of nintedanib was ~20 μM for *KRAS* wild-type cells and 5.0 μM for *KRAS*-mutant cells (Fig. S4 and Table S5). These data strongly suggest that nintedanib preferentially suppresses the proliferation of *KRAS*-mutant cancer cells.

### Nintedanib promotes mitochondrial fusion processes and triggers apoptosis in KRAS-mutant cell lines

**Fig. 3:**
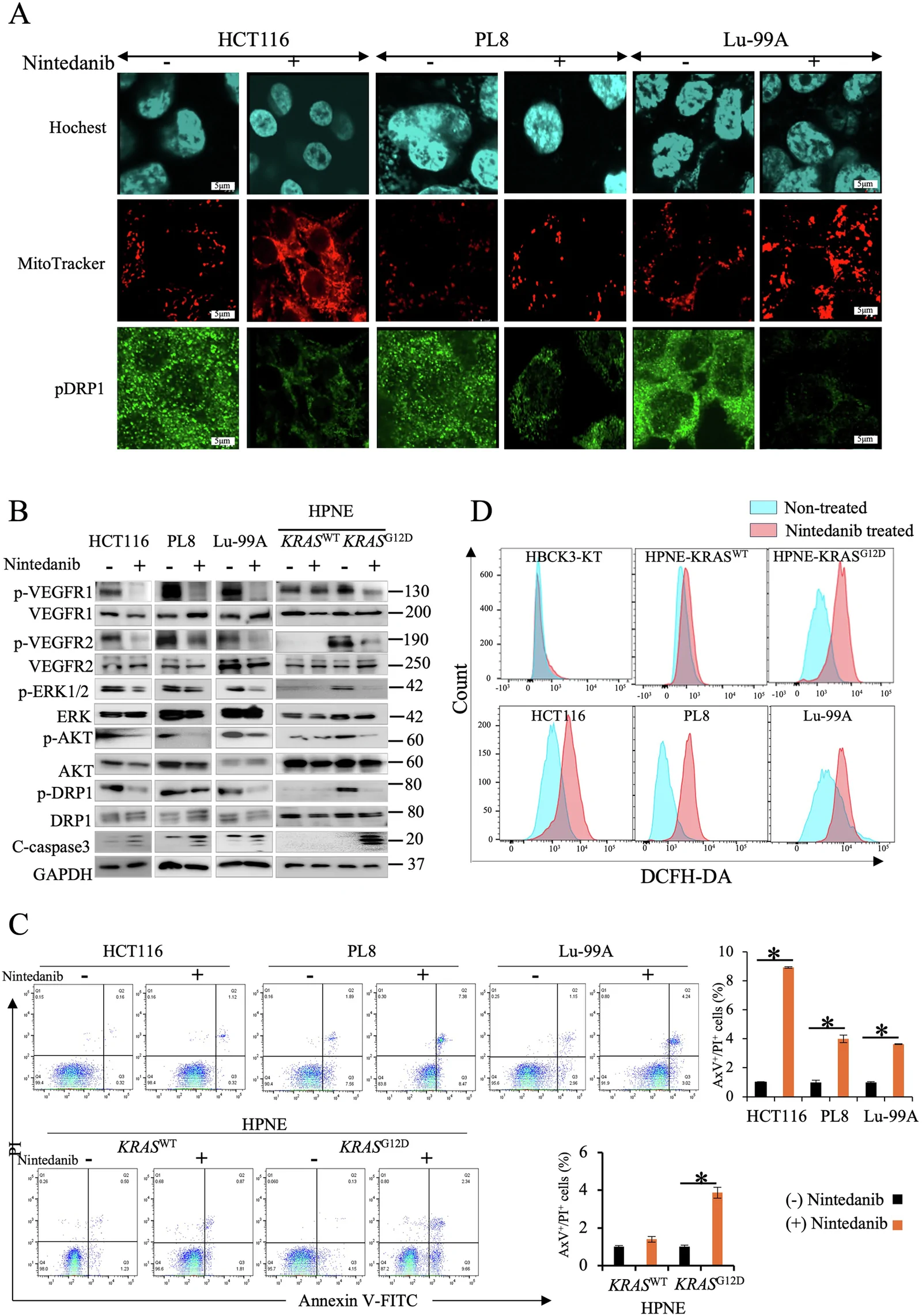
Role of nintedanib in mitochondrial morphology and apoptosis in *KRAS*-mutant cells. **A** Mitochondria were visualized using MitoTracker (red), and nuclei were counterstained with Hoechst (blue) in HCT116 (upper left panel), PL8 (upper center panel), and Lu-99A (upper right) cells. Cells were immunostained with Drp1 antibody (green) and Hoechst (blue) in the mitochondria, as depicted in the left middle panel (HCT116), middle center panel (PL8), and middle right panels (Lu99A). **B** Western blot analysis of p-VEGFR1-Y1213, VEGFR1, p-VEGFR2-Y1175, VEGFR2, pERK-Thr202/Tyr204, tERK, p-AKT-S473, AKT, pDRP1-S616, DRP1, C-caspase3, and GAPDH in HCT116, PL8, Lu-99A, and HPNE (*KRAS*^WT^ and *KRAS*^G12D^) cells treated with nintedanib (7.5 µM) for 48 h. **C** Flow cytometry analysis using Annexin V^+^/PI^+^ staining. Representative plots of AxV-PI-based staining are presented on the left. The graphs on the right display the percentage of AxV^+^/PI^+^ apoptotic cells after nintedanib treatment (7.5 µM) for 48 h measured using LSRFortessa X-20 Flow Cytometer (BD Biosciences). Data indicate mean ± SE (n = 3). Asterisks denote significant differences between nintedanib-treated cells and nontreated cells (HCT116, PL8, Lu-99A, HPNE, and HPNE-KRAS^G12D^) (*p < 0.05). **D** DCFH-DA-based DCF assay: Effect of DCFH-DH (10 ${\rm{\mu }}$M, 45 min) on ROS production in nintedanib-treated cells (red) compared with that in nontreated cells (blue) in HBEC3-KT (*KRAS*^*WT*^), HPNE (*KRAS*^*WT*^), HPNE (*KRAS-*mutant), and HCT116, PL8, and Lu-99A (*KRAS*-mutant) cell lines.

To clarify the biochemical and molecular mechanisms underlying the action of nintedanib on *KRAS-*mutant cancer cells, we evaluated mitochondrial morphology after nintedanib treatment using MitoTracker, a dye that labels functional mitochondria independent of cell viability. Confocal microscopy revealed that nintedanib increased the mitochondrial fusion signals, which were characterized by an elongated tubular network structure, in *KRAS*-mutant HCT116, PL8, and Lu-99A cells (Fig. 3A). We then determined the effect of nintedanib on the phosphorylation of dynamin-related protein 1 (DRP1), a vital mediator of mitochondrial fission. Remarkably, treatment with nintedanib attenuated the phosphorylation of DRP1 in *KRAS*-mutant cells, suggesting a regulatory effect of nintedanib on mitochondrial fission processes (Fig. 3A, B). Western blot analyses further confirmed that nintedanib treatment reduced the phosphorylation levels of several key signaling proteins, including VEGFR1, VEGFR2, ERK, and AKT (Fig. 3B). Moreover, the addition of nintedanib to KRAS-mutant cell lines (HCT116, PL8, Lu-99A, and HPNE-*KRAS*^G12D^) significantly increased the percentage of apoptotic cells, indicating that nintedanib effectively induces apoptosis in these KRAS-mutant strains (Fig. 3C). Similarly, the levels of cleaved caspase3 markedly increased in the nintedanib-treated KRAS-mutant cells (Fig. 3B). To further investigate the mechanism underlying nintedanib-induced apoptosis, we measured the levels of intracellular reactive oxygen species (ROS) using the DCFH-DA assay. Remarkably, nintedanib treatment increased the production of ROS in *KRAS*-mutant cells (HPNE-*KRAS*^G12D^, HCT116, PL8, and Lu-99A), whereas the ROS levels in *KRAS* wild-type cells (HBEC3-KT and HPNE-WT) remained unchanged under both treated and nontreated conditions (Fig. 3D). Overall, these findings suggest that nintedanib selectively induces apoptosis through mitochondrial hyperfusion and increased oxidative stress in *KRAS*-mutant cells.

### VEGFR2 regulates the activity of KRAS-GTP in the cancer cell line

**Fig. 4:**
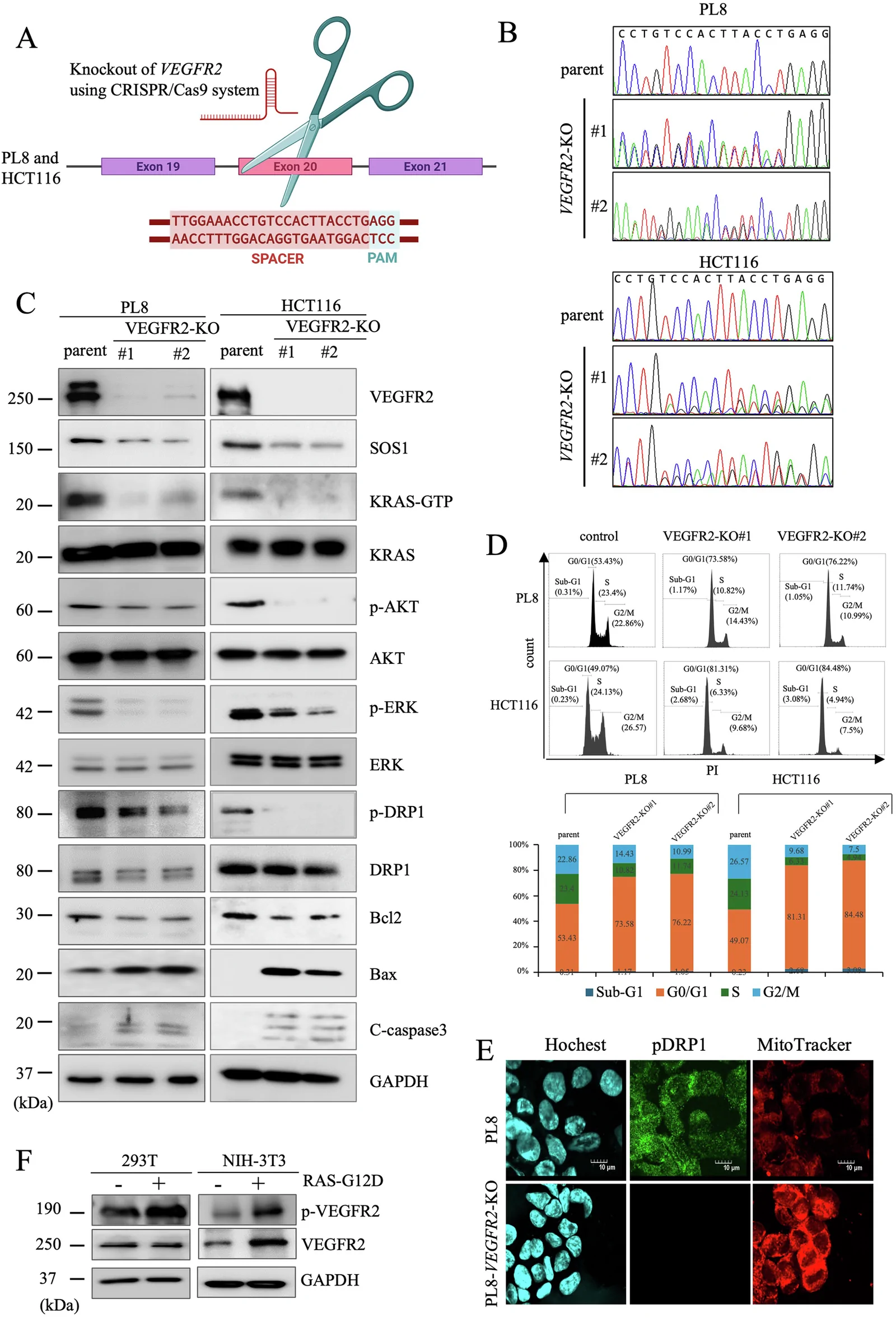
Effect of KRAS-GTP on PL8 VEGFR2-KO and HCT116 VEGFR2-KO cells. Generation of vascular endothelial growth factor receptor 2 (*VEGFR2*)-knockout (VEGFR2-KO) cell clones using the CRISPR/Cas9 system with the human PL8 an HCT116 cell line. **A** A single-cell line guide RNA sequence was designed against exon 20 of the *VEGFR2* locus. **B** The genomic sequence analysis of VEGFR2 in PL8 and HCT116 cells was compared with their parental cells. **C** Protein expression levels of SOS1, KRas-GTP, total KRAS, AKT-S473, AKT, ERK1/2 (Thr202/Tyr204), tERK, DRP1-S616, DRP1, Bcl2, C-caspase 3, VEGFR2, and GAPDH were analyzed by western blotting in PL8 and PL8-VEGFR2-KO cells. **D** Cell cycle was analyzed via Cyto Flex Srt and FlowJosoftware.G0/G1-phase ratio was elevated in VEGFR2-KO cells compared to their parental cells. **E** Mitochondria were visualized using the MitoTracker probe (red). Nuclei were stained with Hoechst (blue), pDRP1 (green) in PL8 (upper panel), and PL8-VEGFR2-KO (down panel). **F** Effect of exogenous RAS-GTP expression on the protein levels of pVEGFR2 in 293 T and 3T3 cells.

To clarify the role of VEGFR2 in *KRAS*-mutated cancer cells, we generated VEGFR2 knockout (referred to as VEGFR2-KO/PL8 and VEGFR2-KO/HCT116) cell clones #1 and #2 using a KRAS-mutated pancreatic cancer cell line PL8, colon cancer cell line HCT116 and lung cancer cell line A549 by targeting exon 20 of the *KDR*/*VEGFR2* gene (Figs. 4A, S5). The successful knockout of *VEGFR2* was confirmed by Sanger sequencing and western blotting (Figs. 4B, 4C and S5). Cell growth analysis using the MTT assay revealed significantly decreased proliferation in VEGFR2-KO cells compared with that in the parental PL8 and HCT116 cells (Fig. S6). Western blot analysis showed that SOS1 and RAS-GTP levels and the phosphorylation levels of AKT, ERK, and DRP1 readily decreased in *VEGFR2*-KO/PL8, *VEGFR2*-KO/HCT116 and *VEGFR2*-KO/A549 cells compared with those in parental cells. Moreover, the protein expression of the proapoptotic proteins Bax and cleaved caspase-3 increased, whereas that of the antiapoptotic protein Bcl2 decreased in the *VEGFR2*-KO/PL8 and VEGFR2-KO/HCT116 cell clones (Fig. 4C, Fig. S5). To gain additional insights, we conducted cell cycle analysis in VEGFR2 knockout and parental cells. The results revealed an increased proportion of cells in the G0/G1 phase in VEGFR2-KO cells compared to the parental cells (Fig. 4D), indicating that VEGFR2 knockout promotes apoptosis and induces cell-cycle arrest in PL8 and HCT116 cells.

In addition, the protein expression of DRP1 decreased in *VEGFR2*-KO/PL8 and *VEGFR2*-KO/HCT116 cells (Fig. 4C, E). Interestingly, confocal imaging revealed a substantial increase in the elongated, tubular mitochondrial structures in *VEGFR2*-KO/*KRAS*-mutant cells compared with those in parental cells (Fig. 4E).

To clarify the involvement of KRAS-GTP in VEGFR2 expression, we introduced the exogenous expression of *KRAS*^G12D^ in 293 T cells and a mouse fibroblast cell line 3T3 cells. Exogenous *KRAS*^G12D^ increased the expression levels of VEGFR2, suggesting that mutant KRAS^G12D^ induces the phosphorylation of the VEGFR2-mediated oncogenic signal (Fig. 4F).

### VEGFR2 is preferentially expressed in human pancreatic cancer tissues

**Fig. 5:**
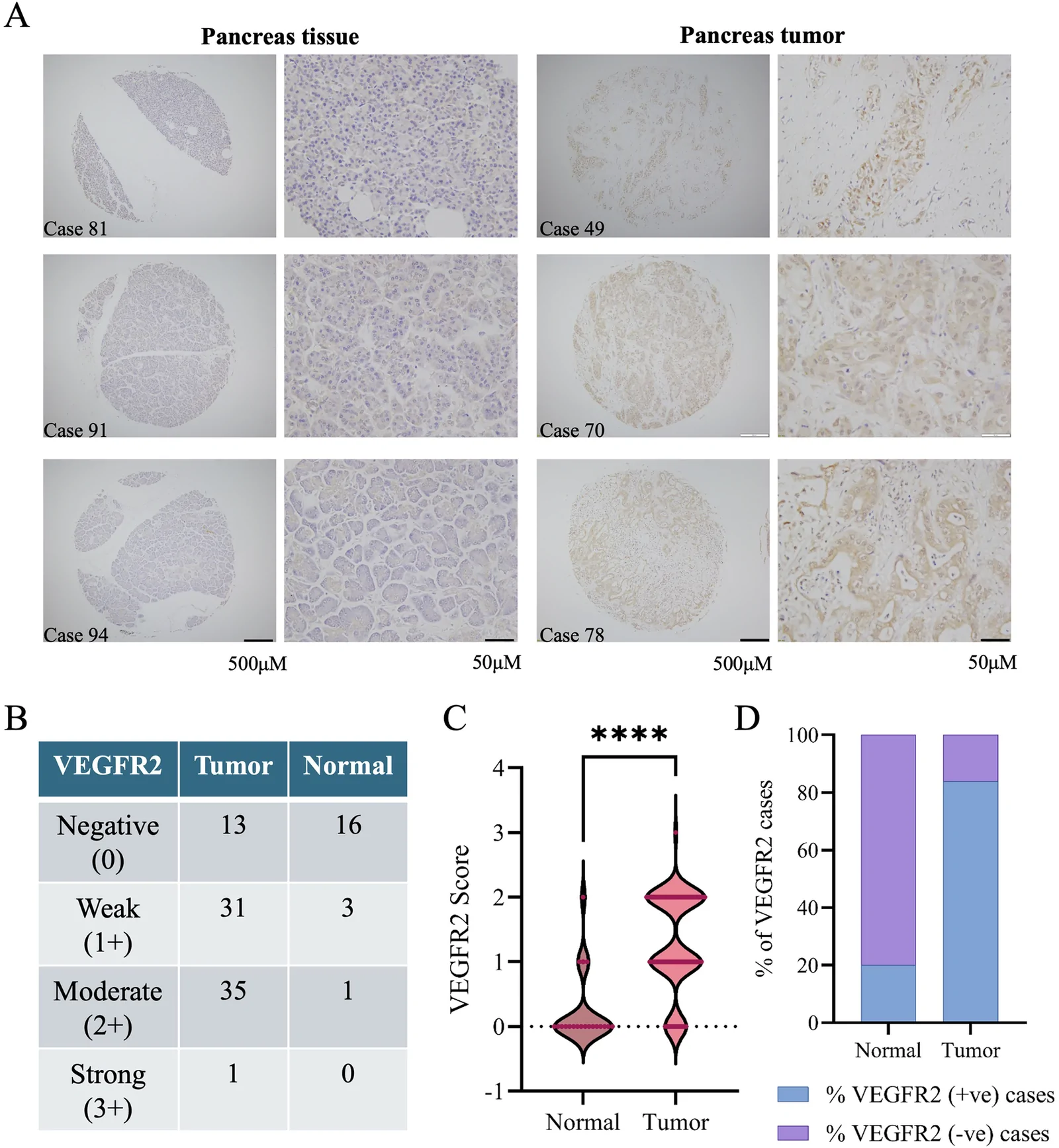
Immunohistochemistry (IHC) for vascular endothelial growth factor receptor (VEGFR2) expression. **A** Expression in VEGFR2-negative pancreas tissue pleural (cases 81, 91, and 94) and VEGFR2-positive pancreatic tumor (cases 49, 70, and 78). **B** Summary of IHC results in pancreatic tumor tissues. The immunoreactivities were independently evaluated by two investigators. The intensity of staining was scored as strong (3+), moderate (2+), weak (1+), or negative (0). **C** Violin plot depicting VEGFR2 scores (Y-axis) in normal and pancreatic tumor tissues. Scores were independently evaluated by two investigators. Statistical analysis was conducted using the Mann–Whitney test; ****p < 0.0001. **D** The bar graph represents the percentage of total cases with VEGFR2 expression (strong, moderate, and weak) in normal pancreatic and tumor tissues.

To investigate the expression of VEGFR2, p53 in human pancreatic cancer tissues, we conducted immunohistochemical analysis with 80 human pancreatic cancer tissues and 20 normal pancreatic tissues (Table 1 and Fig. 5A, Fig. S7). In the microscopic analysis, we detected 1 strong (3+), 35 moderate (2+), and 31 weak (1+) VEGFR2-positive signals in the 80 pancreatic cancer tissues, but only 1 moderate and 3 weak signals in the 20 normal pancreatic tissues (Table 1 and Fig. 5B, C). Accordingly, the positivity rate for VEGFR2 signals in pancreatic cancer tissues (67 of 80 samples; 83%) was significantly higher than that in normal pancreatic tissues (4 of 20 samples; 20%; Fig. 5D), suggesting that VEGFR-mediated signaling is active in pancreatic cancer tissues.

**Table 1:** Summary of immunohistochemistry in this study.

Case No.	Age	Sex	Organ/Anatomic Site	Pathology diagnosis	Grade	Stage	Type	VEGFR2 score	P53 status
1	41	F	Pancreas	Duct adenocarcinoma	1	IIB	Malignant	1+	+
2	65	M	Pancreas	Duct adenocarcinoma	1	IIA	Malignant	2+	-
3	58	F	Pancreas	Duct adenocarcinoma	1	IB	Malignant	2+	-
4	72	F	Pancreas	Duct adenocarcinoma	1	IIA	Malignant	2+	-
5	60	M	Pancreas	Duct adenocarcinoma	1	IIA	Malignant	2+	-
6	41	F	Pancreas	Duct adenocarcinoma	1	IIA	Malignant	2+	+
7	65	M	Pancreas	Duct adenocarcinoma	1	IIA	Malignant	2+	+
8	58	F	Pancreas	Duct adenocarcinoma	-	IB	Malignant	2+	+
9	72	F	Pancreas	Duct adenocarcinoma	1	IIA	Malignant	2+	-
10	60	M	Pancreas	Duct adenocarcinoma	1	IIA	Malignant	2+	-
11	52	M	Pancreas	Duct adenocarcinoma	1	IIB	Malignant	2+	-
12	65	M	Pancreas	Duct adenocarcinoma with cataplasia	2	IIB	Malignant	0	-
13	41	M	Pancreas	Duct adenocarcinoma	2	IB	Malignant	1+	-
14	44	M	Pancreas	Duct adenocarcinoma	2	IIA	Malignant	1+	-
15	55	F	Pancreas	Duct adenocarcinoma	1	IB	Malignant	1+	+
16	52	M	Pancreas	Duct adenocarcinoma	1	IIB	Malignant	2+	-
17	65	M	Pancreas	Duct adenocarcinoma with cataplasia	2	IIB	Malignant	0	-
18	41	M	Pancreas	Duct adenocarcinoma	2	IB	Malignant	1+	-
19	44	M	Pancreas	Duct adenocarcinoma	2	IIA	Malignant	1+	-
20	55	F	Pancreas	Duct adenocarcinoma	1	IB	Malignant	1+	+
21	55	M	Pancreas	Duct adenocarcinoma	1	IIA	Malignant	0	+
22	55	M	Pancreas	Duct adenocarcinoma	2	IB	Malignant	0	-
23	34	M	Pancreas	Duct adenocarcinoma	2	IIA	Malignant	1+	-
24	42	M	Pancreas	Duct adenocarcinoma	1	IIA	Malignant	1+	-
25	44	M	Pancreas	Duct adenocarcinoma	3	IIA	Malignant	1+	-
26	55	M	Pancreas	Duct adenocarcinoma	1	IIA	Malignant	2+	+
27	55	M	Pancreas	Duct adenocarcinoma	2	IB	Malignant	1+	-
28	34	M	Pancreas	Duct adenocarcinoma	2	IIA	Malignant	2+	-
29	42	M	Pancreas	Duct adenocarcinoma	2	IIA	Malignant	2+	-
30	44	M	Pancreas	Duct adenocarcinoma	3	IIA	Malignant	2+	-
31	59	M	Pancreas	Duct adenocarcinoma	2	IIA	Malignant	2+	+
32	53	M	Pancreas	Duct adenocarcinoma	3	IIA	Malignant	0	-
33	52	M	Pancreas	Duct adenocarcinoma	2	IIA	Malignant	1+	-
34	48	F	Pancreas	Duct adenocarcinoma	2	IIA	Malignant	1+	-
35	68	F	Pancreas	Duct adenocarcinoma	3	IIA	Malignant	1+	+
36	59	M	Pancreas	Duct adenocarcinoma	2	IIA	Malignant	2+	-
37	53	M	Pancreas	Duct adenocarcinoma	2	IIA	Malignant	1+	-
38	52	M	Pancreas	Duct adenocarcinoma	2	IIA	Malignant	3+	-
39	48	F	Pancreas	Duct adenocarcinoma	2	IIA	Malignant	2+	-
40	68	F	Pancreas	Duct adenocarcinoma	3	IIA	Malignant	1+	+
41	41	M	Pancreas	Duct adenocarcinoma	3	III	Malignant	1+	-
42	64	M	Pancreas	Duct adenocarcinoma	3	IIA	Malignant	1+	-
43	58	F	Pancreas	Duct adenocarcinoma	3	IIA	Malignant	1+	-
44	60	F	Pancreas	Duct adenocarcinoma	3	III	Malignant	1+	-
45	72	F	Pancreas	Duct adenocarcinoma	3	IIA	Malignant	2+	+
46	41	M	Pancreas	Duct adenocarcinoma	3	III	Malignant	2+	-
47	64	M	Pancreas	Duct adenocarcinoma	3	IIA	Malignant	2+	-
48	58	F	Pancreas	Duct adenocarcinoma	1	IIA	Malignant	1+	-
49	60	F	Pancreas	Duct adenocarcinoma	3	III	Malignant	2+	-
50	72	F	Pancreas	Duct adenocarcinoma	3	IIA	Malignant	2+	+
51	56	M	Pancreas	Duct adenocarcinoma	3	IIB	Malignant	0	-
52	63	M	Pancreas	Duct adenocarcinoma	3	IB	Malignant	1+	-
53	62	M	Pancreas	Duct adenocarcinoma	3	IB	Malignant	1+	-
54	59	M	Pancreas	Duct adenocarcinoma	3	IB	Malignant	1+	-
55	60	F	Pancreas	Duct adenocarcinoma	3	IV	Malignant	2+	-
56	56	M	Pancreas	Duct adenocarcinoma	3	IIB	Malignant	1+	-
57	63	M	Pancreas	Duct adenocarcinoma	3	IB	Malignant	2+	-
58	62	M	Pancreas	Duct adenocarcinoma	3	IB	Malignant	2+	-
59	59	M	Pancreas	Duct adenocarcinoma	3	IB	Malignant	2+	+
60	60	F	Pancreas	Duct adenocarcinoma	3	IV	Malignant	2+	-
61	53	F	Pancreas	Duct adenocarcinoma	3	IIA	Malignant	0	-
62	50	M	Pancreas	Duct adenocarcinoma	3	IIB	Malignant	0	-
63	51	M	Pancreas	Duct adenocarcinoma	3	IIA	Malignant	0	-
64	62	F	Pancreas	Duct adenocarcinoma	3	IIA	Malignant	1+	+
65	78	M	Pancreas	Duct adenocarcinoma	3	IB	Malignant	2+	+
66	53	F	Pancreas	Duct adenocarcinoma	3	IIA	Malignant	2+	-
67	50	M	Pancreas	Duct adenocarcinoma	3	IIB	Malignant	1+	-
68	51	M	Pancreas	Duct adenocarcinoma	3	IIA	Malignant	1+	-
69	62	F	Pancreas	Duct adenocarcinoma	3	IIA	Malignant	1+	+
70	78	M	Pancreas	Duct adenocarcinoma	3	IB	Malignant	2+	+
71	39	F	Pancreas	Duct adenocarcinoma	3	IIA	Malignant	0	-
72	55	M	Pancreas	Duct adenocarcinoma	3	IB	Malignant	1+	-
73	76	F	Pancreas	Duct adenocarcinoma	3	III	Malignant	2+	+
74	76	M	Pancreas	Duct adenocarcinoma	3	IIA	Malignant	0	-
75	57	F	Pancreas	Duct adenocarcinoma	3	IB	Malignant	1+	+
76	39	F	Pancreas	Duct adenocarcinoma	3	IIA	Malignant	2+	-
77	55	M	Pancreas	Duct adenocarcinoma	3	IB	Malignant	2+	-
78	76	F	Pancreas	Duct adenocarcinoma with necrosis	3	III	Malignant	2+	+
79	76	M	Pancreas	Duct adenocarcinoma	3	IIA	Malignant	0	-
80	57	F	Pancreas	Duct adenocarcinoma	3	IB	Malignant	0	+
81	40	M	Pancreas	Pancreas tissue	-	-	Normal	0	-
82	47	M	Pancreas	Pancreas tissue	-	-	Normal	0	-
83	25	M	Pancreas	Pancreas tissue	-	-	Normal	0	-
84	30	M	Pancreas	Pancreas tissue	-	-	Normal	1+	-
85	30	M	Pancreas	Pancreas tissue	-	-	Normal	0	-
86	40	M	Pancreas	Pancreas tissue	-	-	Normal	0	-
87	47	M	Pancreas	Pancreas tissue	-	-	Normal	0	-
88	25	M	Pancreas	Pancreas tissue	-	-	Normal	1+	-
89	30	M	Pancreas	Pancreas tissue	-	-	Normal	2+	-
90	30	M	Pancreas	Pancreas tissue	-	-	Normal	1+	-
91	50	M	Pancreas	Pancreas tissue	-	-	Normal	0	-
92	35	M	Pancreas	Pancreas tissue	-	-	Normal	0	-
93	38	M	Pancreas	Pancreas tissue	-	-	Normal	0	-
94	35	F	Pancreas	Pancreas tissue	-	-	Normal	0	-
95	21	F	Pancreas	Pancreas tissue	-	-	Normal	0	-
96	50	M	Pancreas	Pancreas tissue	-	-	Normal	0	-
97	35	M	Pancreas	Pancreas tissue	-	-	Normal	0	-
98	38	M	Pancreas	Pancreas tissue	-	-	Normal	0	-
99	35	F	Pancreas	Pancreas tissue	-	-	Normal	0	-
100	21	F	Pancreas	Pancreas tissue	-	-	Normal	0	-

Staining intensity was scored as strong (3+), moderate (2+), weak (1+), or negative (0), p53 positive (+), p53 negative (−).

### Nintedanib significantly retards tumor growth in PL8 xenograft mice

**Fig. 6:**
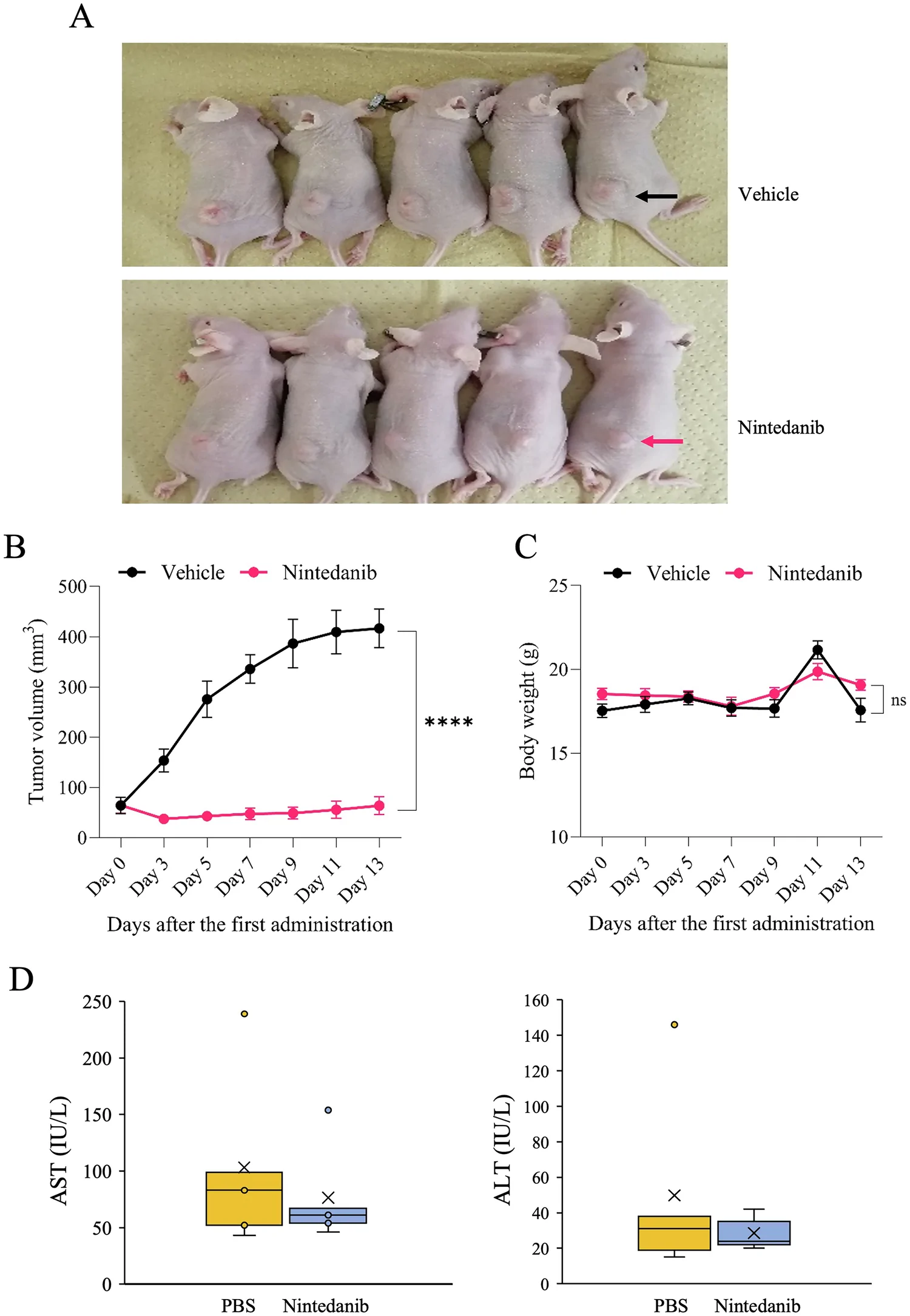
Effect of nintedanib on the growth of PL8 tumor cells in vivo*.* PL8 cells (5 × 10^6^/mouse) were subcutaneously xenografted into nude mice. When tumors reached ~60 mm^3^ (day 0), nintedanib (15 mg/kg body weight) or vehicle (PBS) was intraperitoneally administered on days 0, 3, 5, 7, 9, 11, and 13 into xenografted mice. **A** Representative image depicting tumor-bearing xenografted mice in each group. Growth curves of (**B**) tumor volume (mm^3^) and (**C**) mouse body weight (g) during nintedanib treatment. Data are expressed as mean ± SEM (n = 5). ****p < 0.0001, ns, not significant; two-way ANOVA. **D** Blood chemistry analysis (AST and ALT) after nintedanib treatment compared with vehicle control.

Finally, we investigated the effect of nintedanib on tumor growth in vivo using the *KRAS*-mutated pancreatic cancer cell line PL8 with immunodeficient nude mice. Remarkably, nintedanib administration significantly decreased tumor growth compared with that in the vehicle control group, as shown in Fig. 6A, B. Importantly, treatment with nintedanib did not result in significant weight loss in mice (Fig. 6C). Moreover, blood chemistry tests revealed that nintedanib administration exerted no effect on the levels of aspartate aminotransferase (AST) and alanine aminotransferase (ALT) (Fig. 6D). These results support the hypothesis that targeting VEGFR with nintedanib is a viable therapeutic strategy for *KRAS*-mutated pancreatic cancers, emphasizing its potential as a promising candidate for molecular-targeted anticancer therapies.

## Discussion

*KRAS* mutations are frequently detected in several aggressive malignancies, including pancreatic, colorectal, lung, and certain hematological cancers. These mutations result in the constitutive activation of downstream signaling pathways, particularly the MAPK and PI3K/AKT pathways [29]. Current anticancer therapies targeting KRAS remain limited, largely due to the absence of binding pockets on the KRAS protein, as well as the unknown molecular mechanism regulating the KRAS-mediated signaling pathway in KRAS-mutated tumors. Therefore, the development of novel therapeutic strategies remains a major unmet clinical need [30, 31]. Recent progress in the development of direct KRAS inhibitors, particularly against KRAS G12C, has demonstrated that mutant-selective targeting is feasible in certain contexts; however, effective therapeutic options remain limited for many other *KRAS* variants [10, 13, 32, 33]. In addition, accumulating evidence indicates that distinct *KRAS* mutations, such as G12C, G12D, G12V, G13D, and Q61 alterations, differ in their biochemical behavior, downstream signaling output, and therapeutic vulnerability [16, 34–36]. Therefore, a better understanding of context-dependent KRAS signaling and alternative therapeutic targets remains highly important [16, 34, 37]. Our results emphasize the potential of nintedanib as a promising candidate for targeting *KRAS*-mutated cancers.

In our screening with a large chemical library, nintedanib emerged as a potent agent that selectively inhibited the proliferation of *KRAS*-mutant cells. This selectivity is particularly remarkable, considering that several attempts for directly targeting KRAS have been unsuccessful, primarily due to the “undruggable” nature of the protein [13]. In this study, we identified an upstream signaling component toward KRAS, specifically VEGFR2, as a candidate molecule in our human cell model. In fact, VEGFR2 has been reported to play a pivotal role in maintaining KRAS-mediated oncogenic signaling [25].

The MTT assays revealed that nintedanib exhibited significantly lower IC_50_ values in *KRAS*-mutant cells than in their wild-type counterparts, emphasizing its selectivity. Importantly, nintedanib exhibited superior antiproliferative efficacy than other VEGFR inhibitors, such as axitinib and motesanib, across various cellular and mutational contexts. Mechanistically, nintedanib suppressed the expression of downstream signaling molecules, including pAKT and pERK, indicating that VEGFR2 inhibition disrupts critical survival and proliferation pathways [38, 39]. Its improved efficacy is probably due to its broader activity against multiple tyrosine kinases, including FGFRs and PDGFRs, in addition to VEGFR2, which amplifies the antiangiogenic and antiproliferative effects [40, 41]. Combined with favorable pharmacokinetic properties and dosing advantages, these features probably explain the superior clinical and preclinical performance of nintedanib [42, 43]. Furthermore, the induction of apoptosis in *KRAS*-mutant cells was accompanied by mitochondrial hyperfusion and elevated ROS production, suggesting that nintedanib disrupts mitochondrial homeostasis and redox balance to selectively compromise the survival advantage conferred by mutant *KRAS*.

An interesting aspect of our study is the regulatory feedback loop identified between KRAS-GTP and VEGFR2 expression. The overexpression of *KRAS*^G12D^ resulted in increased VEGFR2 protein levels, suggesting a complex interaction that contributes to tumor aggressiveness in KRAS-driven cancers [44]. Moreover, our knock-in cell lines and *KRAS*-mutant cells exhibited increased VEGFR2 phosphorylation. Recent data further support the finding that *KRAS* mutations are associated with increased VEGFR2 phosphorylation in both tumor vasculature [45] and cancer cells. *KRAS* mutations can drive metabolic reprogramming that modulates VEGFR2 activity [46]. Furthermore, *KRAS*-driven cancers exhibit resistance to anti-VEGF therapies, which correlate with alterations in VEGFR2 signaling pathways [47]. Altogether, these data suggest that *KRAS* mutations promote VEGFR2 activation, thereby contributing to tumor angiogenesis and therapy resistance, whereas nintedanib selectively counteracts this process by inducing apoptosis and suppressing tumor growth. Supporting this assumption further, VEGFR2 knockout models using PL8, HCT116 and A549 cells demonstrated that the loss of VEGFR2 decreased both SOS1 protein levels and KRAS-GTP activity, accompanied by reduced AKT-ERK signaling and increased apoptosis, leading to cell cycle arrest at the G0/G1 phase and a subsequent reduction in cell growth. This result strongly suggests that VEGFR2 plays a pivotal role in maintaining KRAS activity in *KRAS*-mutant cells. Furthermore, recent evidence indicates that ERK2-dependent phosphorylation of Drp1 plays a vital role in *KRAS*-driven tumor growth by facilitating mitochondrial fission, which is crucial for metabolic reprogramming and tumor progression, particularly in pancreatic cancer [48–51]. Inhibition of Drp1 can disrupt glycolytic flux and impair mitochondrial metabolic functions in cells harboring oncogenic *KRAS* [52–54]. In this study, treatment of *KRAS*-mutant cells with nintedanib reduced Drp1 activity and mitochondrial fission in VEGFR2-KO *KRAS*-mutant cells. Consistent with these findings, VEGFR2 inhibition by nintedanib suppressed the *KRAS* mutation–driven increase in mitochondrial fission, suggesting that nintedanib can also block KRAS downstream proliferation signals mediated through mitochondria, emphasizing its potential as a highly effective molecular-targeted therapy.

Our immunohistochemical results showed that VEGFR2 is predominantly expressed in *KRAS*-mutant cell lines and patient-derived pancreatic cancer tissues. IHC analysis of human pancreatic cancer samples revealed significantly higher VEGFR2 expression in tumor tissues than in normal tissues, with an 83% positivity rate suggesting the involvement of VEGFR2 in *KRAS*-driven oncogenesis and its potential as a biomarker for disease progression and therapeutic targeting.

In vivo, nintedanib treatment significantly inhibited tumor growth in *KRAS*-mutant xenograft models without remarkable toxicity, as evidenced by stable body weights and normal liver enzyme levels. These data are promising, because tolerability remains a critical consideration in cancer therapy [8]. Nonetheless, our study has limitations. The dependence on tumor cell lines, cell line–derived xenografts, and tissue samples restricts the translatability of our findings. Future studies should incorporate patient-derived xenograft models, which better recapitulate the tumor microenvironment and heterogeneity, as demonstrated in previous research that evaluated the efficacy of nintedanib in breast, lung, pancreatic, and kidney cancers [55–58]. More importantly, comprehensive studies are required to validate these preliminary findings and establish clinical relevance.

In conclusion, our study identified nintedanib as a potent therapeutic candidate against *KRAS*-mutant cancers by targeting the VEGFR2–ERK signaling axis. The correlation between *KRAS* mutations and VEGFR2 expression, supported by molecular and clinical data, suggests VEGFR2 as both a driver and a biomarker of KRAS-driven oncogenesis. Further clinical studies are necessary to determine the utility of nintedanib in treating patients harboring *KRAS* mutations.

## Materials and methods

### Cell culture

The human normal epithelial cell lines hTERT-HPNE (CRL-4023) and HBEC3-KT (CRL-4051) were obtained from the American Type Culture Collection (Manassas, VA). Additional cell lines, including A549, Lu-99A, DLD1, HCT116, Sw-480, AsPC-1, PL5, and PL8, were generously provided by Dr. Ben Ho Park at Johns Hopkins University (Baltimore, MD). HBEC3-KT cells were cultured in Ham’s F-12 medium supplemented with 10% fetal bovine serum (FBS) and 1% penicillin. The remaining cell lines (HPNE, Sachi, A549, Lu-99A, DLD1, HCT116, Sw-480, AsPC-1, PL5, and PL8) were maintained in RPMI-1640 medium (Wako, Osaka, Japan) supplemented with 10% FBS (Sigma) and 1% penicillin–streptomycin (Wako). All cultures were incubated at 37 °C in a humidified atmosphere containing 5% CO_2_. Cells were routinely subcultured upon reaching ~80% confluence, maintaining optimal growth conditions. The culture media were changed every 2–3 days, and cell viability and morphology were evaluated regularly to ensure healthy growth and appropriate characteristics of the cell lines. All tested cell lines are free of mycoplasma contamination.

### Gene knock-in using the prime editing system

The epegRNA plasmid was constructed using the Golden Gate cloning strategy, involving the assembly of a BsaI-digested epegRNA backbone (pU6-tevopreq1-GG-acceptor, Addgene: #174038) [59, 60]. After digestion, the reaction mixture was incubated at 37 °C for 4 h. Verification of proper digestion was accomplished by gel electrophoresis, where the expected bands for a correctly digested pegRNA backbone appeared at 2.2 kb. The 2.2-kb fragment was isolated using the Qiagen Gel Extraction Kit. Three annealed double-stranded DNA fragments—the epegRNA spacer sequence, epegRNA scaffold, and epegRNA extension sequence (which includes the phosphate-buffered saline (PBS) and RT template)—were prepared based on primers listed in Table S6. These fragments were mixed and ligated with the epegRNA backbone. To establish a knock-in clone, we transfected 1 µg of the epegRNA G12D plasmid along with the pCMV-PEmax-P2A-GFP (Addgene #180020) plasmid into HPNE and HBEC3-KT cells (1 × 10^6^ cells) using a 4D-Nucleofector instrument (Lonza Japan, Tokyo, Japan). After 3 days posttransfection, GFP-expressing cells were sorted by fluorescence-activated cell sorting (FACS) using a BD FACSAria™ III single-cell sorter (BD Biosciences, San Jose, CA, USA). An individual clone was isolated, expanded, and later used for downstream biological assays.

### Gene knock-in using the CRISPR editing system

The gRNA plasmid was constructed by Golden Gate cloning using BsaI-digested PX458 (Addgene #48138). The backbone was digested at 37 °C for 1 h, purified by Qiagen Gel Extraction, and ligated with an annealed double-stranded gRNA based on the primer 5ʹ-AAACTTGTGGTAGTTGGAGC-3ʹ. Moreover, 900-bp exon 2 fragments from RPMI-8226 genomic DNA were amplified and cloned into pcDNA3.1. For knock-in, 1 μg of PX458-RAS gRNA and pcDNA3.1 KRAS^G12A^ donor were cotransfected into 1 × 10^6^ Sachi cells using the 4D-Nucleofector. After 3 days, GFP-positive cells were sorted by FACS, and single clones were isolated, expanded, and used for further analysis.

### Gene knockout using the CRISPR/Cas9 system

The CRISPR/Cas9 system was used to knock out VEGFR2 in the PL8 cell line, according to established procedures [61]. The pSpCas9(BB)-2A-GFP (PX458) plasmid was generously provided by Feng Zhang (plasmid #48138; Addgene, Watertown, MA, USA) [62]. Briefly, sgRNA sequences were selected using an optimized CRISPR design tool (http://crispr.mit.edu/). The selected sgRNA sequences for VEGFR2 were 5′-GAAACCTGTCCACTTACCTG-3′ in exon 20. Plasmids expressing hCas9 and sgRNA were generated by ligating oligonucleotides into the BbsI site of PX458 (*VEGFR2*/PX458). To construct the expression vector KRAS^G12D^, the cDNA fragments of KRAS^G12D^ were amplified by PCR using the Prime STAR Max DNA polymerase (Takara Bio, Otsu, Japan). The cDNA fragments were then introduced into the PiggyBac expression vector (Addgene Cat no #203312). Backbone PiggyBac was used as a control vector. To establish a knockout (KO) clone, 1 μg of the *VEGFR2*/PX458 plasmid was transfected into PL8, HCT116 and A549 cells (1 × 10^6^) using the 4D-Nucleofector instrument (Lonza Japan, Tokyo, Japan). After 3 days, GFP-expressing cells were sorted by FACS (BD FACSAria™ III Cell Sorter; BD Biosciences, San Jose, CA, USA). A single clone was selected, expanded, and used for the biological assays.

### Cell growth assay

Cell proliferation was analyzed using the MTT colorimetric assay. Cells were seeded into 96-well plates at a density of 1 × 10^3^/well and allowed to adhere and grow for predetermined time intervals (0, 24, 48, and 72 h). At each time point, 10 μL of MTT solution (5 mg/mL; Sigma-Aldrich) was added to each well, followed by incubation for 4 h at 37 °C to enable viable cells to convert MTT into purple formazan crystals. After incubation, the formazan was completely solubilized, and absorbance was measured at 595 nm using a SpectraMAX M5 microplate reader (Molecular Devices, Sunnyvale, CA, USA). The measured optical density (OD) correlated directly with the number of metabolically active cells. All experiments were conducted in triplicate, and data are expressed as mean ± standard deviation.

### Western blot analysis

Western blotting was performed to detect specific proteins in sample extracts as described previously [63, 64]. Proteins were first separated by SDS-PAGE and then transferred to a PVDF membrane (Millipore Cat. no IPVH00010), which was blocked with 5% nonfat milk in TBST to minimize nonspecific binding. The membrane was incubated overnight with primary antibodies (listed in Table S7) at 4°C, followed by washing and application of HRP-conjugated secondary antibodies for 1 h at room temperature. Immune complexes were detected using ImmunoStar LD chemiluminescent substrate (FUJIFILM Wako Chemicals, USA, Cat no. 292-69903), and bands were visualized using an LAS-4000 image analyzer. Protein expression levels were quantified by densitometry using the ImageJ software, with normalization to GAPDH as a loading control. All experiments were conducted in triplicate for reproducibility.

### Soft agar colony formation assay

The soft agar colony formation assay was performed as described previously [65, 66]. The number of colonies was counted using the Colony Counter software (Keyence, Tokyo, Japan). Data are expressed as mean ± SEM (n = 5).

### Migration assay

A total of 10,000 cells suspended in 100 μL serum-free medium were added to the upper chambers of a transwell (8 μm for a 24-well plate; Millipore, Tokyo, Japan), as described previously [63], and culture medium was added to the lower chambers. After 24 h, the cells were fixed with formalin and stained with 0.1% crystal violet. The number of colonies was manually counted under a microscope.

### Annexin V assay

Cells were plated into six-well culture plates (5 × 10^5^/well) and treated with nintedanib (7.5 µg/mL) for 48 h. Subsequently, the cells were exposed to annexin V (Ax)-FITC and propidium iodide (PI) (10 μg/mL) for 15 min at 25 °C. Fluorescence intensities were quantified by FACS analysis (LSRFortessa X-20 Flow Cytometer, BD Biosciences, Franklin Lakes, NJ, USA) [60].

### Immunofluorescence

Cells were cultured on glass coverslips and fixed with a 4% paraformaldehyde solution for 20 min at 25 °C. Then, the cells were permeabilized with PBS containing 0.1% Triton X-100, blocked in PBS containing 7% serum for 30 min, and incubated with primary antibody (pDRP1) followed by Alexa Fluor–conjugated secondary antibodies (Invitrogen). Cell staining was performed using MitoTracker (stock solution, 1 mM; diluted at 1:10,000) for 1 h at 37 °C to visualize mitochondria. After staining, the cells were washed with PBS and fixed with cold paraformaldehyde (3.2% in PBS) for 20 min at room temperature. After additional washing steps, the samples were mounted using PermaFluor, and images were captured using the FLUOVIEW FV3000 Series of Confocal Laser Scanning Microscopes.

### DCFH-DA-based DCF assay

Cells were seeded into six-well plates at a density of 3 × 10^5^/well. After 24 h, the cells were exposed to 5.0 μM nintedanib for 1 day and then incubated with 10 μM DCFH-DA (2ʹ,7ʹ-dichlorodihydrofluorescein diacetate) in culture medium for 45 min, protected from light. After incubation, the dye was removed, and the cells were washed with PBS. The cells were then trypsinized, collected by centrifugation at 1000 rpm for 5 min, and resuspended in PBS in 0.5-mL tubes on ice. Intracellular ROS levels were determined by flow cytometry using LSRFortessa X-20 (BD Biosciences) [60].

### Cell cycle analysis

Briefly, 5 × 10^5^ cells were seeded into each well of a six-well tissue culture plate. After 48 h, the cells were harvested and fixed overnight in 70% ethanol at −30 °C. The cells were then resuspended, incubated with RNase A (100 μg/mL) at 37 °C for 30 min, and subsequently stained with propidium iodide (PI; 100 μg/mL) for another 30 min at 4 °C. Finally, the PI-labeled cells were analyzed by flow cytometry (LSRFortessa X-20 Flow Cytometer, BD Biosciences).

### Immunohistochemistry

Immunohistochemical analysis was performed according to previously established protocols [67, 68]. A human pancreatic cancer tissue array (PA1002b; US Biomax, Rockville, MD, USA) was used, wherein tissue sections were treated with primary antibodies against VEGFR2 (CST 2479) and p53 (DO-7, Agilent). Negative controls included normal rabbit IgG or omission of the primary antibody. Immunoreactivity was determined independently by two investigators (S.K. and H.M.), and staining intensity was scored as strong (3+), moderate (2+), weak (1+), or negative (0).

### cDNA microarray analysis

The cDNA microarray analysis was conducted according to the manufacturer’s protocol (Agilent Technologies), as described previously [64]. Briefly, cDNA synthesis and cRNA labeling were performed using cyanine 3 (Cy3) dye with the Agilent Low Input Quick Amp Labeling Kit (Agilent Technologies). The Cy3-labeled cRNA was then purified, fragmented, and hybridized onto a Human Gene Expression 8 × 60 K v2 Microarray Chip, which contains 62,969 Entrez Gene RNAs, using the Agilent Gene Expression Hybridization Kit. Raw and normalized microarray data have been deposited in the Gene Expression Omnibus database at the National Center for Biotechnology Information (accession number GSE312012). Gene set enrichment analysis was conducted according to standard protocols.

### Screening of the anticancer drug library

A library consisting of 1374 compounds (Selleck Catalog No. L2000-Z295656, FDA-Approved Drug Library 1300) was screened. Specifically, 500 cells of each of the following lines were seeded into 384-well cell culture plates: Sachi-KRAS^WT^, HPNE-KRAS^WT^, Sachi-KRAS^G12A^, and HPNE-KRAS^G12D^ (*KRAS* gene mutants). The cells were then incubated for 24 h at 37 °C. Next, a library of 1543 compounds was added to each well to achieve a final concentration of 7.5 ${\rm{\mu }}$M, and the cells were incubated at 37 °C for an additional 72 h, after which cell viability was evaluated using the MTT assay. Absorbance was measured at 595 nm using a SpectraMAX M5 spectrophotometer (Molecular Devices, Sunnyvale, CA, USA). Control wells containing no drug treatment were considered to have 100% viability, allowing for the calculation of relative cell viability after drug exposure.

### Xenograft experiments

All animal experiments were performed according to the protocols (approval number-2023-101) approved by the ethical committee of Aichi Medical University and followed established guidelines. Female nude mice (BALB/cSlc-nu/nu) (aged 5 weeks, each weighing 14–15 g) were purchased from CLEA Japan, Inc (Tokyo, Japan) and bred at the Institute of Animal Experiments in Aichi Medical University in specified pathogen-free animal facilities. PL8 cells (1 × 10^7^) were injected subcutaneously into these mice. When the inoculated tumor reached ~60 mm^3^ (day 0), the mice were randomly divided into two groups (treatment and control groups). Nintedanib (15 mg/kg body weight) was intraperitoneally administered on days 0, 3, 5, 7, 9, 11, and 13 to each mouse in the treatment group. The control group received PBS as a vehicle control. The tumor volume was measured on days 0, 3, 5, 7, 9, 11, and 13 and calculated using the modified ellipsoid formula (1/2 × length × width^2^). Data are expressed as the mean ± SEM (n = 5).

### Blood chemistry

After 2 weeks of nintedanib treatment, mice in both the control and treatment groups were anesthetized with isoflurane, and ~1 mL of blood was collected in a heparin tube. The blood samples were centrifuged at 800 × *g* for 20 min for collecting serum, which was then examined for AST and ALT by the Nagahama Life Science Laboratory (Oriental Yeast Co., Ltd., Shiga, Japan).

### Statistical analysis

The data are presented as mean ± SE (M) values. Statistical significance among groups was assessed using one-way analysis of variance followed by Dunnett’s comparison. All statistical analyses were conducted using GraphPad Prism and/or SPSS version 23.0 (SPSS Inc., Chicago, IL, USA).

## Supplementary information

KRAS-Supplemental Figures

Table S1

Table S2

Table S3

Table S4

Table S5

Table S6

Table S7

All the full and uncropped version of the western blot images

## Data Availability

All accessible data are provided either in the main manuscript or in the supplementary materials. Complete and unaltered Western blot data utilized in the study are available in the supplementary information. Furthermore, specific inquiries, data, and materials can be obtained upon reasonable request from the corresponding author.

## References

[1] Uniyal P, Kashyap VK, Behl T, Parashar D, Rawat R. KRAS mutations in cancer: understanding signaling pathways to immune regulation and the potential of immunotherapy. Cancers. 2025;17:785. 40075634 10.3390/cancers17050785 PMC11899378

[2] Norton C, Shaw MS, Rubnitz Z, Smith J, Soares HP, Nevala-Plagemann CD, et al. KRAS mutation status and treatment outcomes in patients with metastatic pancreatic adenocarcinoma. JAMA Netw Open. 2025;8:e2453588. 39777438 10.1001/jamanetworkopen.2024.53588 PMC11707629

[3] Wolfgang CL, Herman JM, Laheru DA, Klein AP, Erdek MA, Fishman EK, et al. Recent progress in pancreatic cancer. CA Cancer J Clin. 2013;63:318–48. 23856911 10.3322/caac.21190 PMC3769458

[4] Bardeesy N, DePinho RA. Pancreatic cancer biology and genetics. Nat Rev Cancer. 2002;2:897–909. 12459728 10.1038/nrc949

[5] Moore MJ, Goldstein D, Hamm J, Figer A, Hecht JR, Gallinger S, et al. Erlotinib plus gemcitabine compared with gemcitabine alone in patients with advanced pancreatic cancer: a phase III trial of the National Cancer Institute of Canada Clinical Trials Group. J Clin Oncol. 2023;41:4714–20. 37847995 10.1200/JCO.22.02770

[6] Karapetis CS, Khambata-Ford S, Jonker DJ, O’Callaghan CJ, Tu D, Tebbutt NC, et al. K-ras mutations and benefit from cetuximab in advanced colorectal cancer. N Engl J Med. 2008;359:1757–65. 18946061 10.1056/NEJMoa0804385

[7] Douillard JY, Oliner KS, Siena S, Tabernero J, Burkes R, Barugel M, et al. Panitumumab-FOLFOX4 treatment and RAS mutations in colorectal cancer. N Engl J Med. 2013;369:1023–34. 24024839 10.1056/NEJMoa1305275

[8] Riely GJ, Ladanyi M. KRAS mutations: an old oncogene becomes a new predictive biomarker. J Mol Diagn. 2008;10:493–5. 18832458 10.2353/jmoldx.2008.080105 PMC2570631

[9] Reck M, van Zandwijk N, Gridelli C, Baliko Z, Rischin D, Allan S, et al. Erlotinib in advanced non-small cell lung cancer: efficacy and safety findings of the global phase IV Tarceva Lung Cancer Survival Treatment study. J Thorac Oncol. 2010;5:1616–22. 20736854 10.1097/JTO.0b013e3181f1c7b0

[10] Canon J, Rex K, Saiki AY, Mohr C, Cooke K, Bagal D, et al. The clinical KRAS(G12C) inhibitor AMG 510 drives anti-tumour immunity. Nature. 2019;575:217–23. 31666701 10.1038/s41586-019-1694-1

[11] Ou SI, Janne PA, Leal TA, Rybkin II, Sabari JK, Barve MA, et al. First-in-human phase I/IB dose-finding study of Adagrasib (MRTX849) in patients with advanced KRAS(G12C) solid tumors (KRYSTAL-1). J Clin Oncol. 2022;40:2530–8. 35167329 10.1200/JCO.21.02752 PMC9362872

[12] Massarelli E, Varella-Garcia M, Tang X, Xavier AC, Ozburn NC, Liu DD, et al. KRAS mutation is an important predictor of resistance to therapy with epidermal growth factor receptor tyrosine kinase inhibitors in non-small-cell lung cancer. Clin Cancer Res. 2007;13:2890–6. 17504988 10.1158/1078-0432.CCR-06-3043

[13] Ostrem JM, Peters U, Sos ML, Wells JA, Shokat KM. K-Ras(G12C) inhibitors allosterically control GTP affinity and effector interactions. Nature. 2013;503:548–51. 24256730 10.1038/nature12796 PMC4274051

[14] Janakiraman M, Vakiani E, Zeng Z, Pratilas CA, Taylor BS, Chitale D, et al. Genomic and biological characterization of exon 4 KRAS mutations in human cancer. Cancer Res. 2010;70:5901–11. 20570890 10.1158/0008-5472.CAN-10-0192 PMC2943514

[15] Bahar ME, Kim HJ, Kim DR. Targeting the RAS/RAF/MAPK pathway for cancer therapy: from mechanism to clinical studies. Signal Transduct Target Ther. 2023;8:455. 38105263 10.1038/s41392-023-01705-z PMC10725898

[16] Huang L, Guo Z, Wang F, Fu L. KRAS mutation: from undruggable to druggable in cancer. Signal Transduct Target Ther. 2021;6:386. 34776511 10.1038/s41392-021-00780-4 PMC8591115

[17] Peng TR, Wu TW, Yi TY, Wu AJ. Comparative efficacy of Adagrasib and Sotorasib in KRAS G12C-mutant NSCLC: insights from pivotal trials. Cancers. 2024;16:3676. 39518114 10.3390/cancers16213676 PMC11545475

[18] Casacuberta-Serra S, Gonzalez-Larreategui I, Capitan-Leo D, Soucek L. MYC and KRAS cooperation: from historical challenges to therapeutic opportunities in cancer. Signal Transduct Target Ther. 2024;9:205. 39164274 10.1038/s41392-024-01907-z PMC11336233

[19] Punekar SR, Velcheti V, Neel BG, Wong KK. The current state of the art and future trends in RAS-targeted cancer therapies. Nat Rev Clin Oncol. 2022;19:637–55. 36028717 10.1038/s41571-022-00671-9 PMC9412785

[20] Quinlan MP, Settleman J. Isoform-specific Ras functions in development and cancer. Future Oncol. 2009;5:105–16. 19243303 10.2217/14796694.5.1.105

[21] Papke B, Der CJ. Drugging RAS: know the enemy. Science. 2017;355:1158–63. 28302824 10.1126/science.aam7622

[22] Moore AR, Rosenberg SC, McCormick F, Malek S. RAS-targeted therapies: is the undruggable drugged? Nat Rev Drug Discov. 2020;19:533–52. 32528145 10.1038/s41573-020-0068-6 PMC7809886

[23] Singh S, Smith MJ. RAS GTPase signalling to alternative effector pathways. Biochem Soc Trans. 2020;48:2241–52. 33125484 10.1042/BST20200506

[24] Bandaru P, Kondo Y, Kuriyan J. The interdependent activation of son-of-sevenless and Ras. Cold Spring Harb Perspect Med. 2019;9:a031534. 29610148 10.1101/cshperspect.a031534 PMC6360870

[25] Bar-Sagi D, Hall A. Ras and Rho GTPases: a family reunion. Cell. 2000;103:227–38. 11057896 10.1016/s0092-8674(00)00115-x

[26] Chen T, Tang X, Wang Z, Feng F, Xu C, Zhao Q, et al. Inhibition of son of sevenless homologue 1 (SOS1): promising therapeutic treatment for KRAS-mutant cancers. Eur J Med Chem. 2023;261:115828. 37778239 10.1016/j.ejmech.2023.115828

[27] Downward J. Targeting RAS signalling pathways in cancer therapy. Nat Rev Cancer. 2003;3:11–22. 12509763 10.1038/nrc969

[28] McCormick F. KRAS as a therapeutic target. Clin Cancer Res. 2015;21:1797–801. 25878360 10.1158/1078-0432.CCR-14-2662 PMC4407814

[29] Song Y, Bi Z, Liu Y, Qin F, Wei Y, Wei X. Targeting RAS-RAF-MEK-ERK signaling pathway in human cancer: current status in clinical trials. Genes Dis. 2023;10:76–88. 37013062 10.1016/j.gendis.2022.05.006 PMC10066287

[30] Xia Y, Sun M, Huang H, Jin WL. Drug repurposing for cancer therapy. Signal Transduct Target Ther. 2024;9:92. 38637540 10.1038/s41392-024-01808-1 PMC11026526

[31] Santarpia M, Ciappina G, Spagnolo CC, Squeri A, Passalacqua MI, Aguilar A, et al. Targeted therapies for KRAS-mutant non-small cell lung cancer: from preclinical studies to clinical development-a narrative review. Transl Lung Cancer Res. 2023;12:346–68. 36895930 10.21037/tlcr-22-639 PMC9989806

[32] Lito P, Solomon M, Li LS, Hansen R, Rosen N. Allele-specific inhibitors inactivate mutant KRAS G12C by a trapping mechanism. Science. 2016;351:604–8. 26841430 10.1126/science.aad6204 PMC4955282

[33] Hallin J, Engstrom LD, Hargis L, Calinisan A, Aranda R, Briere DM, et al. The KRAS(G12C) inhibitor MRTX849 provides insight toward therapeutic susceptibility of KRAS-mutant cancers in mouse models and patients. Cancer Discov. 2020;10:54–71. 31658955 10.1158/2159-8290.CD-19-1167 PMC6954325

[34] Ryan MB, Corcoran RB. Therapeutic strategies to target RAS-mutant cancers. Nat Rev Clin Oncol. 2018;15:709–20. 30275515 10.1038/s41571-018-0105-0

[35] Hammond DE, Mageean CJ, Rusilowicz EV, Wickenden JA, Clague MJ, Prior IA. Differential reprogramming of isogenic colorectal cancer cells by distinct activating KRAS mutations. J Proteome Res. 2015;14:1535–46. 25599653 10.1021/pr501191a PMC4356034

[36] Vatansever S, Erman B, Gumus ZH. Comparative effects of oncogenic mutations G12C, G12V, G13D, and Q61H on local conformations and dynamics of K-Ras. Comput Struct Biotechnol J. 2020;18:1000–11. 32373288 10.1016/j.csbj.2020.04.003 PMC7191603

[37] Ternet C, Junk P, Sevrin T, Catozzi S, Wahlen E, Heldin J, et al. Analysis of context-specific KRAS-effector (sub)complexes in Caco-2 cells. Life Sci Alliance. 2023;6:e202201670. 36894174 10.26508/lsa.202201670 PMC9998658

[38] Zhou BY, Wang WB, Wu XL, Zhang WJ, Zhou GD, Gao Z, et al. Nintedanib inhibits keloid fibroblast functions by blocking the phosphorylation of multiple kinases and enhancing receptor internalization. Acta Pharmacol Sin. 2020;41:1234–45. 32327724 10.1038/s41401-020-0381-y PMC7608201

[39] Li Q, Zhou X, Yang Y, Zhang Q, Li Z, Han H, et al. Nintedanib abrogates patient vitreous-induced Akt activation and tube formation of human retinal microvascular endothelial cells. Tissue Cell. 2025;94:102817. 40043340 10.1016/j.tice.2025.102817

[40] Boichuk S, Gessel T. Repurposing the tyrosine kinase inhibitors targeting FGFR and VEGFR pathways for cancer therapy: a comprehensive review. Cancers. 2025;17:3354. 41154409 10.3390/cancers17203354 PMC12563661

[41] Schoffski P, Toulmonde M, Estival A, Marquina G, Dudzisz-Sledz M, Brahmi M, et al. Randomised phase 2 study comparing the efficacy and safety of the oral tyrosine kinase inhibitor nintedanib with single agent ifosfamide in patients with advanced, inoperable, metastatic soft tissue sarcoma after failure of first-line chemotherapy: EORTC-1506-STBSG “ANITA. Eur J Cancer. 2021;152:26–40. 34062484 10.1016/j.ejca.2021.04.015

[42] Raghu G, Remy-Jardin M, Richeldi L, Thomson CC, Inoue Y, Johkoh T, et al. Idiopathic pulmonary fibrosis (an Update) and progressive pulmonary fibrosis in adults: an official ATS/ERS/JRS/ALAT clinical practice guideline. Am J Respir Crit Care Med. 2022;205:e18–e47. 35486072 10.1164/rccm.202202-0399ST PMC9851481

[43] Yan S, Xue S, Wang T, Gao R, Zeng H, Wang Q, et al. Efficacy and safety of nintedanib in patients with non-small cell lung cancer, and novel insights in radiation-induced lung toxicity. Front Oncol. 2023;13:1086214. 37637045 10.3389/fonc.2023.1086214 PMC10449572

[44] Dai M, Chen S, Teng X, Chen K, Cheng W. KRAS as a key oncogene in the clinical precision diagnosis and treatment of pancreatic cancer. J Cancer. 2022;13:3209–20. 36118526 10.7150/jca.76695 PMC9475360

[45] Yuan XH, Yang J, Wang XY, Zhang XL, Qin TT, Li K. Association between EGFR/KRAS mutation and expression of VEGFA, VEGFR and VEGFR2 in lung adenocarcinoma. Oncol Lett. 2018;16:2105–12. 30008907 10.3892/ol.2018.8901 PMC6036498

[46] Grillo E, Corsini M, Ravelli C, Zammataro L, Bacci M, Morandi A, et al. Expression of activated VEGFR2 by R1051Q mutation alters the energy metabolism of Sk-Mel-31 melanoma cells by increasing glutamine dependence. Cancer Lett. 2021;507:80–88. 33744390 10.1016/j.canlet.2021.03.007

[47] Hosaka K, Andersson P, Wu J, He X, Du Q, Jing X, et al. KRAS mutation-driven angiopoietin 2 bestows anti-VEGF resistance in epithelial carcinomas. Proc Natl Acad Sci USA. 2023;120:e2303740120. 37428914 10.1073/pnas.2303740120 PMC10629547

[48] Serasinghe MN, Wieder SY, Renault TT, Elkholi R, Asciolla JJ, Yao JL, et al. Mitochondrial division is requisite to RAS-induced transformation and targeted by oncogenic MAPK pathway inhibitors. Mol Cell. 2015;57:521–36. 25658204 10.1016/j.molcel.2015.01.003 PMC4320323

[49] Nagdas S, Kashatus JA, Nascimento A, Hussain SS, Trainor RE, Pollock SR, et al. Drp1 promotes KRas-driven metabolic changes to drive pancreatic tumor growth. Cell Rep. 2019;28:1845–59.e5. 31412251 10.1016/j.celrep.2019.07.031 PMC6711191

[50] Kashatus JA, Nascimento A, Myers LJ, Sher A, Byrne FL, Hoehn KL, et al. Erk2 phosphorylation of Drp1 promotes mitochondrial fission and MAPK-driven tumor growth. Mol Cell. 2015;57:537–51. 25658205 10.1016/j.molcel.2015.01.002 PMC4393013

[51] Adhikary A, Mukherjee A, Banerjee R, Nagotu S. DRP1: At the crossroads of dysregulated mitochondrial dynamics and altered cell signaling in cancer cells. ACS Omega. 2023;8:45208–23. 38075775 10.1021/acsomega.3c06547 PMC10701729

[52] Ma Q, Zhang W, Wu K, Shi L. The roles of KRAS in cancer metabolism, tumor microenvironment and clinical therapy. Mol Cancer. 2025;24:14. 39806421 10.1186/s12943-024-02218-1 PMC11727292

[53] Hu Y, Lu W, Chen G, Wang P, Chen Z, Zhou Y, et al. K-ras(G12V) transformation leads to mitochondrial dysfunction and a metabolic switch from oxidative phosphorylation to glycolysis. Cell Res. 2012;22:399–412. 21876558 10.1038/cr.2011.145 PMC3257361

[54] Chen W, Zhao H, Li Y. Mitochondrial dynamics in health and disease: mechanisms and potential targets. Signal Transduct Target Ther. 2023;8:333. 37669960 10.1038/s41392-023-01547-9 PMC10480456

[55] Falcomata C, Barthel S, Widholz SA, Schneeweis C, Montero JJ, Toska A, et al. Selective multi-kinase inhibition sensitizes mesenchymal pancreatic cancer to immune checkpoint blockade by remodeling the tumor microenvironment. Nat Cancer. 2022;3:318–36. 35122074 10.1038/s43018-021-00326-1 PMC7612546

[56] Navarro P, Bueno MJ, Zagorac I, Mondejar T, Sanchez J, Mouron S, et al. Targeting tumor mitochondrial metabolism overcomes resistance to antiangiogenics. Cell Rep. 2016;15:2705–18. 27292634 10.1016/j.celrep.2016.05.052

[57] Elias R, Tcheuyap VT, Kaushik AK, Singla N, Gao M, Reig Torras O, et al. A renal cell carcinoma tumorgraft platform to advance precision medicine. Cell Rep. 2021;37:110055. 34818533 10.1016/j.celrep.2021.110055 PMC8762721

[58] Pavia-Jimenez A, Tcheuyap VT, Brugarolas J. Establishing a human renal cell carcinoma tumorgraft platform for preclinical drug testing. Nat Protoc. 2014;9:1848–59. 25010905 10.1038/nprot.2014.108 PMC4314943

[59] Chen L, Park JE, Paa P, Rajakumar PD, Prekop HT, Chew YT, et al. Programmable C:G to G:C genome editing with CRISPR-Cas9-directed base excision repair proteins. Nat Commun. 2021;12:1384. 33654077 10.1038/s41467-021-21559-9 PMC7925527

[60] Hasan MN, Hyodo T, Biswas M, Rahman ML, Mihara Y, Karnan S, et al. Flow cytometry-based quantification of genome editing efficiency in human cell lines using the L1CAM gene. PLoS One. 2023;18:e0294146. 37943774 10.1371/journal.pone.0294146 PMC10635454

[61] Ran FA, Hsu PD, Wright J, Agarwala V, Scott DA, Zhang F. Genome engineering using the CRISPR-Cas9 system. Nat Protoc. 2013;8:2281–308. 24157548 10.1038/nprot.2013.143 PMC3969860

[62] Karnan S, Ota A, Murakami H, Rahman ML, Wahiduzzaman M, Hasan MN, et al. CAMK2D: a novel molecular target for BAP1-deficient malignant mesothelioma. Cell Death Discov. 2023;9:257. 37479714 10.1038/s41420-023-01552-5 PMC10362017

[63] Wahiduzzaman M, Karnan S, Ota A, Hanamura I, Murakami H, Inoko A, et al. Establishment and characterization of CRISPR/Cas9-mediated NF2(-/-) human mesothelial cell line: Molecular insight into fibroblast growth factor receptor 2 in malignant pleural mesothelioma. Cancer Sci. 2019;110:180–93. 30417500 10.1111/cas.13871 PMC6317947

[64] Karnan S, Ota A, Murakami H, Rahman ML, Hasan MN, Wahiduzzaman M, et al. Identification of CD24 as a potential diagnostic and therapeutic target for malignant pleural mesothelioma. Cell Death Discov. 2020;6:127. 33298865 10.1038/s41420-020-00364-1 PMC7674463

[65] Karnan S, Hanamura I, Ota A, Takasugi S, Nakamura A, Takahashi M, et al. CD52 is a novel target for the treatment of FLT3-ITD-mutated myeloid leukemia. Cell Death Discov. 2021;7:121. 34035227 10.1038/s41420-021-00446-8 PMC8149417

[66] Karnan S, Hanamura I, Ota A, Vu LQ, Uchino K, Horio T, et al. ARK5 enhances cell survival associated with mitochondrial morphological dynamics from fusion to fission in human multiple myeloma cells. Cell Death Discov. 2024;10:56. 38282096 10.1038/s41420-024-01814-w PMC10822851

[67] Karnan S, Ota A, Hasan MN, Murakami H, Rahman ML, Wahiduzzaman M, et al. Targeting fatty acid synthase suppresses tumor development in NF2/CDKN2A-deficient pleural mesothelioma. Cell Death Dis. 2026;17:287. 10.1038/s41419-026-08481-y. Feb 28. 41764192 PMC13031323

[68] Jahan N, Islam S, Sivasundaram K, Ota A, Naito M, Kuroda J, et al. Role of versican in extracellular matrix formation: analysis in 3D culture. Am J Physiol Cell Physiol. 2025;328:C245–C257. 39656505 10.1152/ajpcell.00495.2024

