# Supplementary material for: Nintedanib inhibits the VEGFR–ERK signaling pathway in human KRAS-mutated cancer cells
Source: Cell Death Dis. 2026 Jun 23;17(1):780. doi: 10.1038/s41419-026-09015-2 (PMC13547290; doi:10.1038/s41419-026-09015-2)
Supplement: Supplementary file 1 [file 41419_2026_9015_MOESM1_ESM.pdf]

Supplementary Figure

Manuscript Title:
Nintedanib inhibits the VEGFR-ERK signaling pathway in human *KRAS*-mutated cancer cells

•Supplementary information includes 7 supplementary figures

Fig. S1

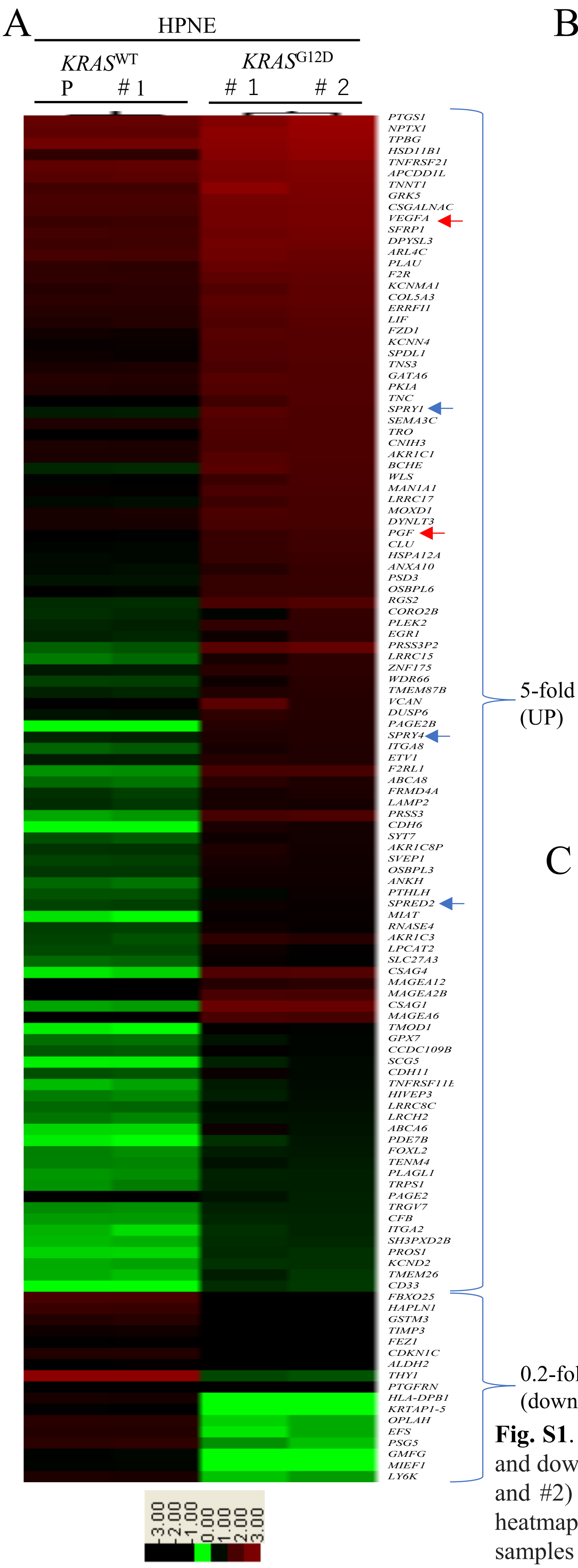

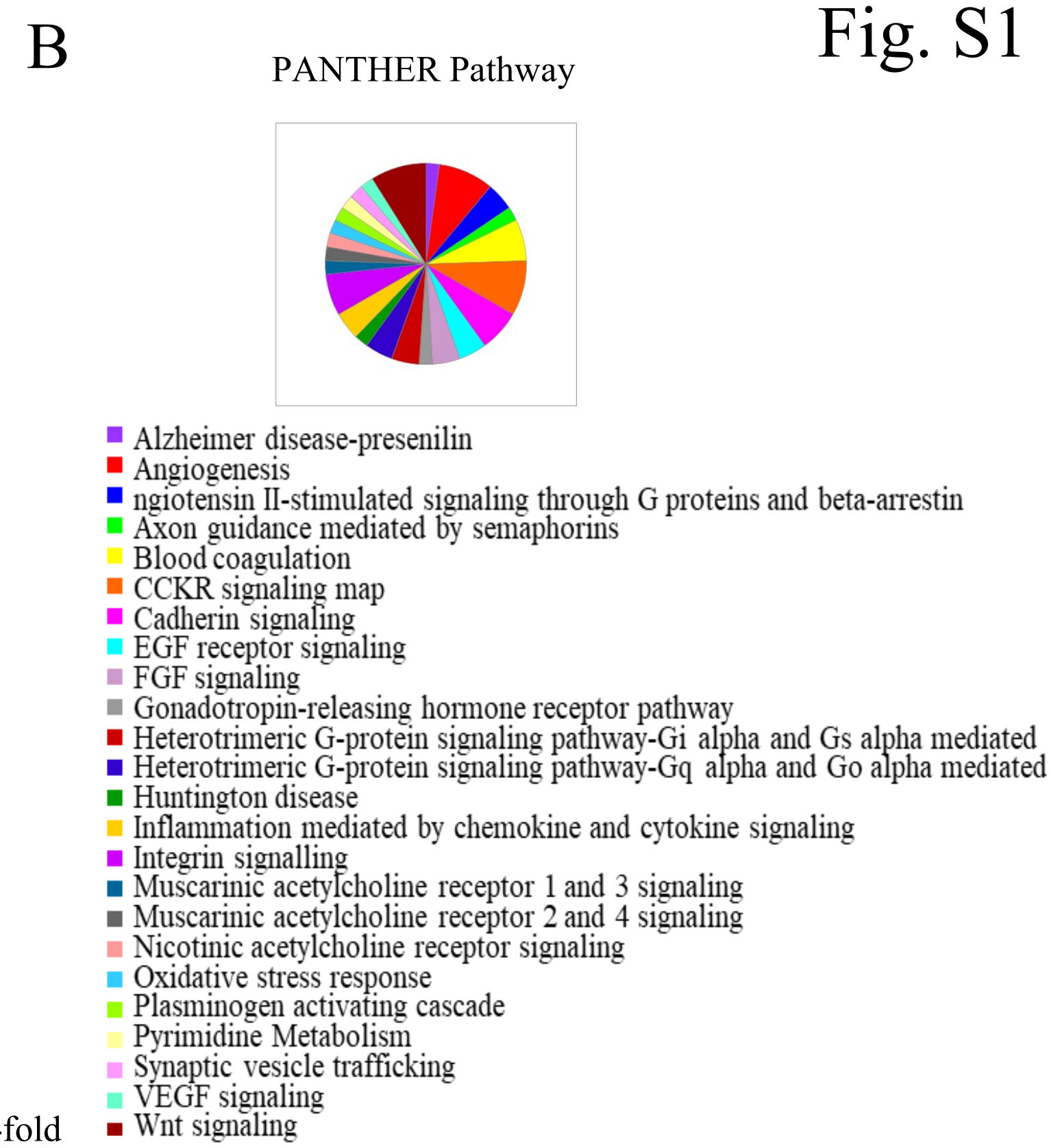

Fig. S1B. Pathway analysis of upregulated 113 genes by using Panther Classification Analysis.

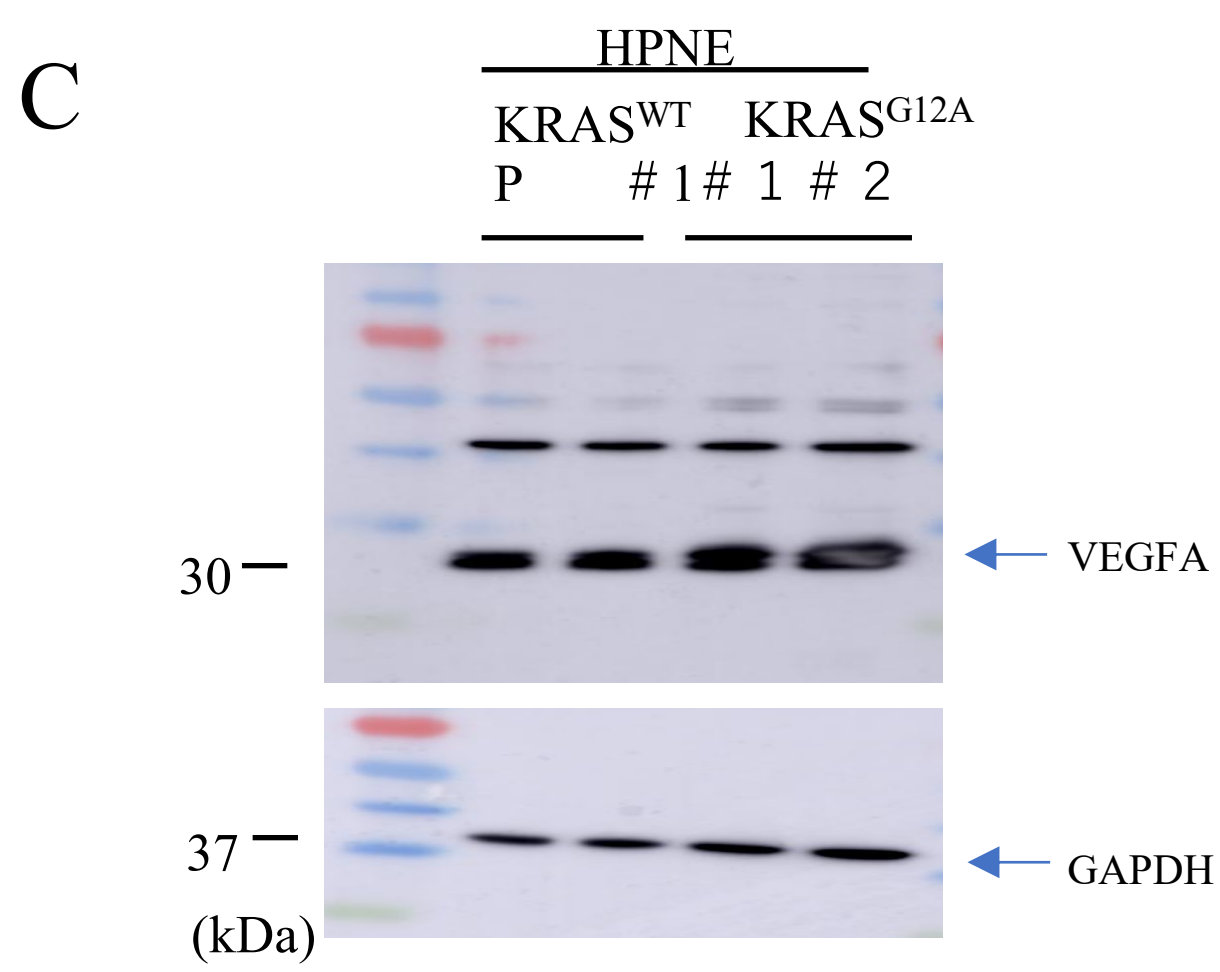

Fig. S1C. The expression of the VEGFA protein was assessed via Western blot analysis, with GAPDH used as the internal control in KRAS^{G12D} cell clones derived from HPNE.

Fig. S1. A. Heat map analysis with upregulated (113 genes; FC >5) and downregulated (13 genes, FC <0.2) genes in *KRAS*^{G12D} cells (#1 and #2) compared with parental (P) and *KRAS*^{WT} (Ctrl) cells. The heatmap was constructed based on the normalized values of all samples using TreeView (Cluster 3.0) http://jtreeview (http://jtreeview.sourceforge.net). The corresponding upregulated or downregulated genes in the heatmap are shown on the right side. FC, fold change. Red arrow indicates genes increase VEGF signaling; Blue arrow indicates genes which are classic negative feedback regulators hyperactive RAS-MAPK signaling.

Fig. S2

A

GSEA analysis with Gene ontology gene sets (C6 Oncogenic Signature)

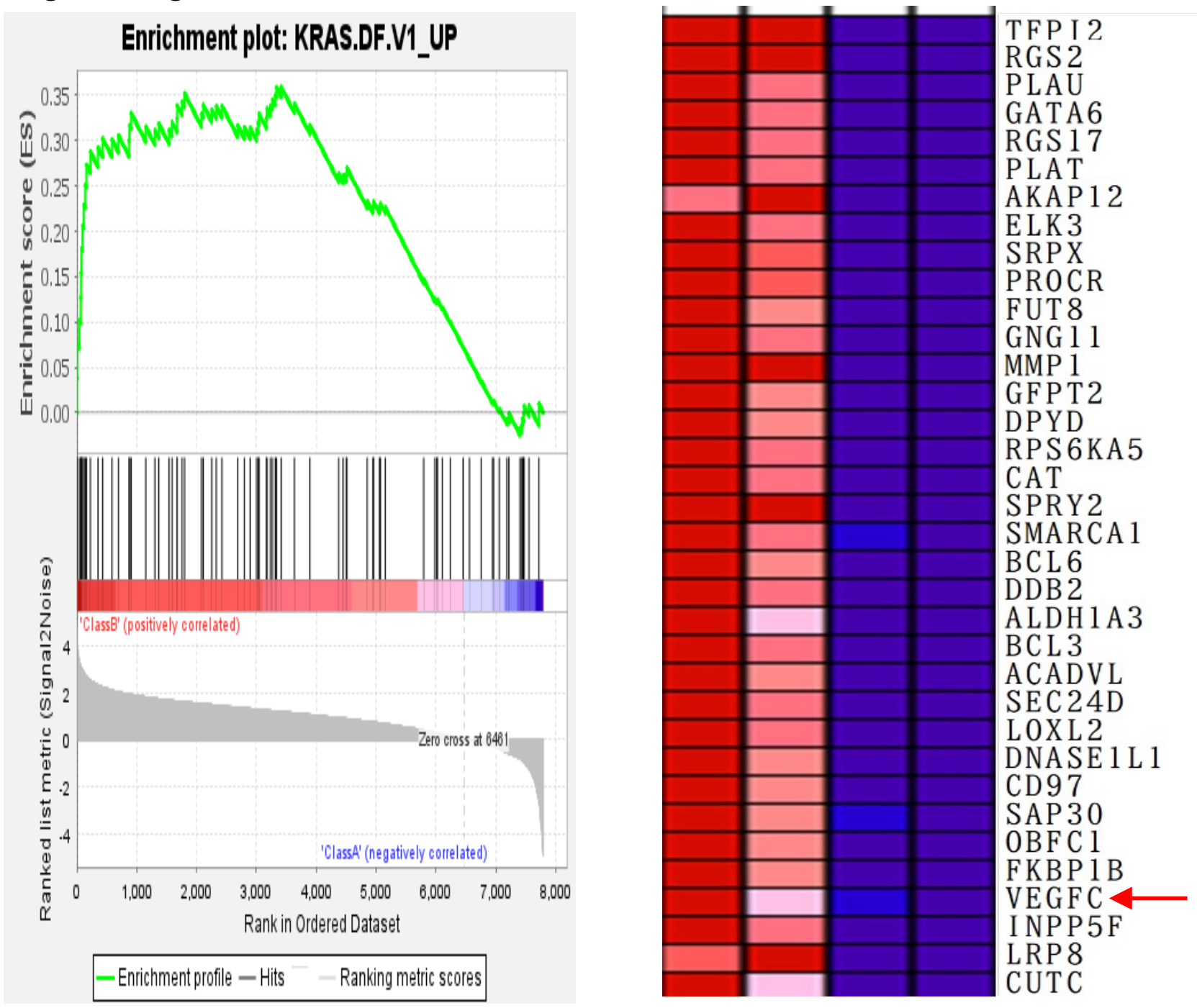

B

GSEA analysis with Gene ontology gene sets (C6 Oncogenic Signature)

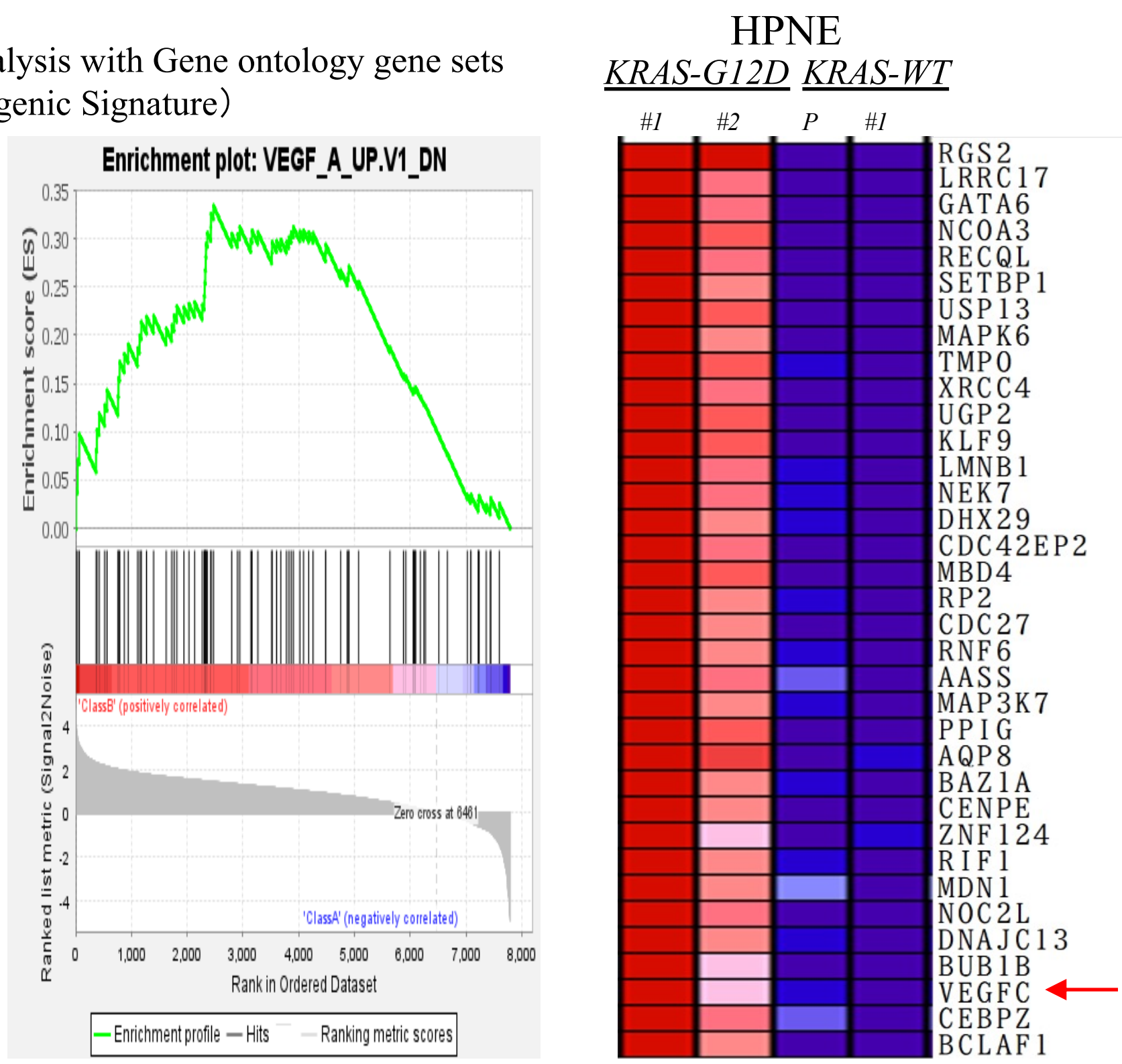

Fig. S2. GSEA in *KRAS*^{G12D} HPNE cells. GSEA were conducted using GSEA v4.33 software and the Molecular Signatures Database (Broad Institute). All the raw data were formatted and applied to **A.** Gene ontology gene sets (c6, Oncogenic Signature). **B.** Curated gene sets (C6 , Oncogenic Signature). Representative enrichment plots and corresponding heatmap image of the indicated gene sets in the KRAS-G12D and KRAS-WT HPNE cells are shown. Genes contributing to enrichment are shown in rows. Expression level is represented as a gradient from high (red) to low (blue). normalized enrichment score.

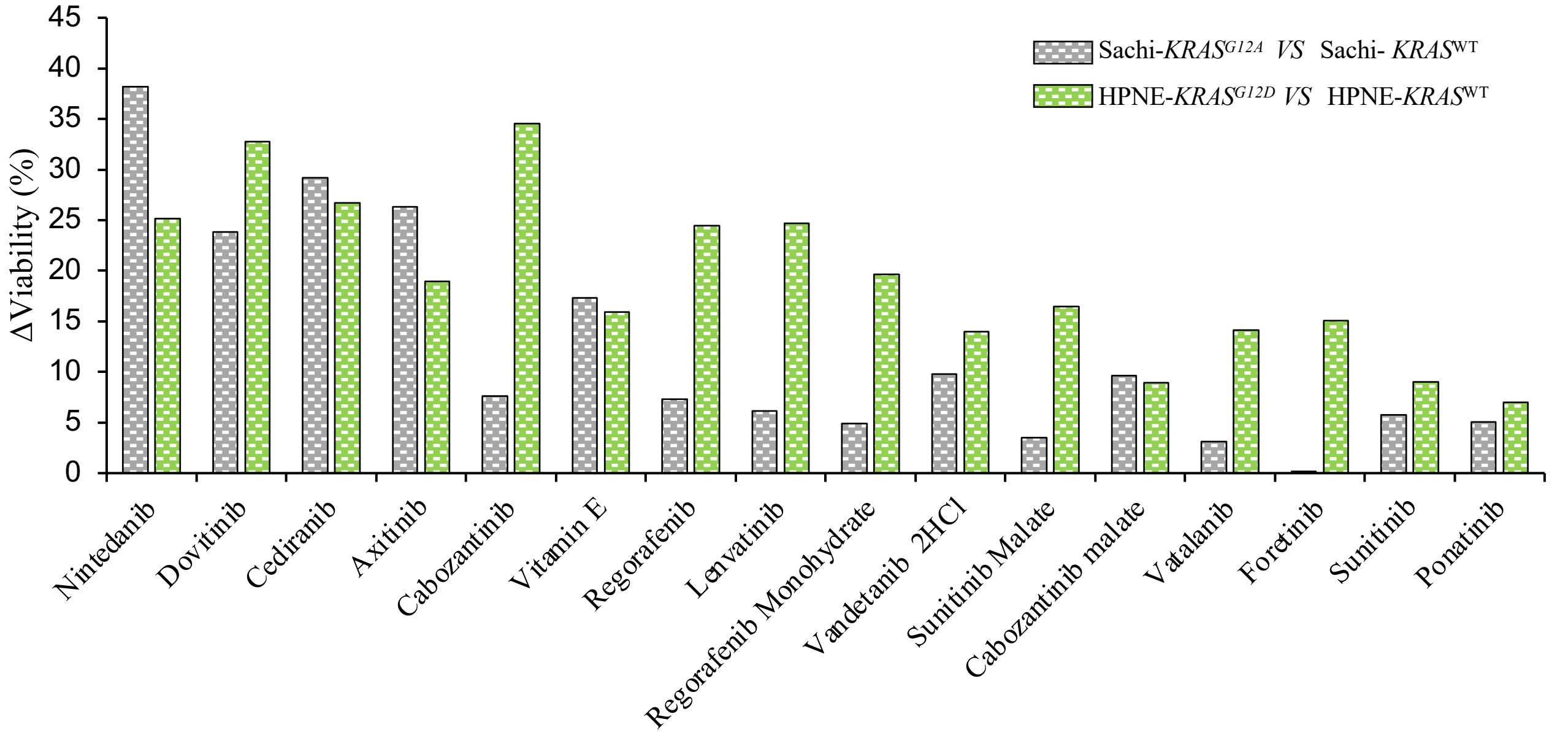

Fig. S3. Identification of VEGFR inhibitors that target KRAS-mutant cell lines. The impact of 16 VEGFR inhibitors in reduction of viability in KRAS mutant cells compared with KRAS wild type cells.

Fig. S4

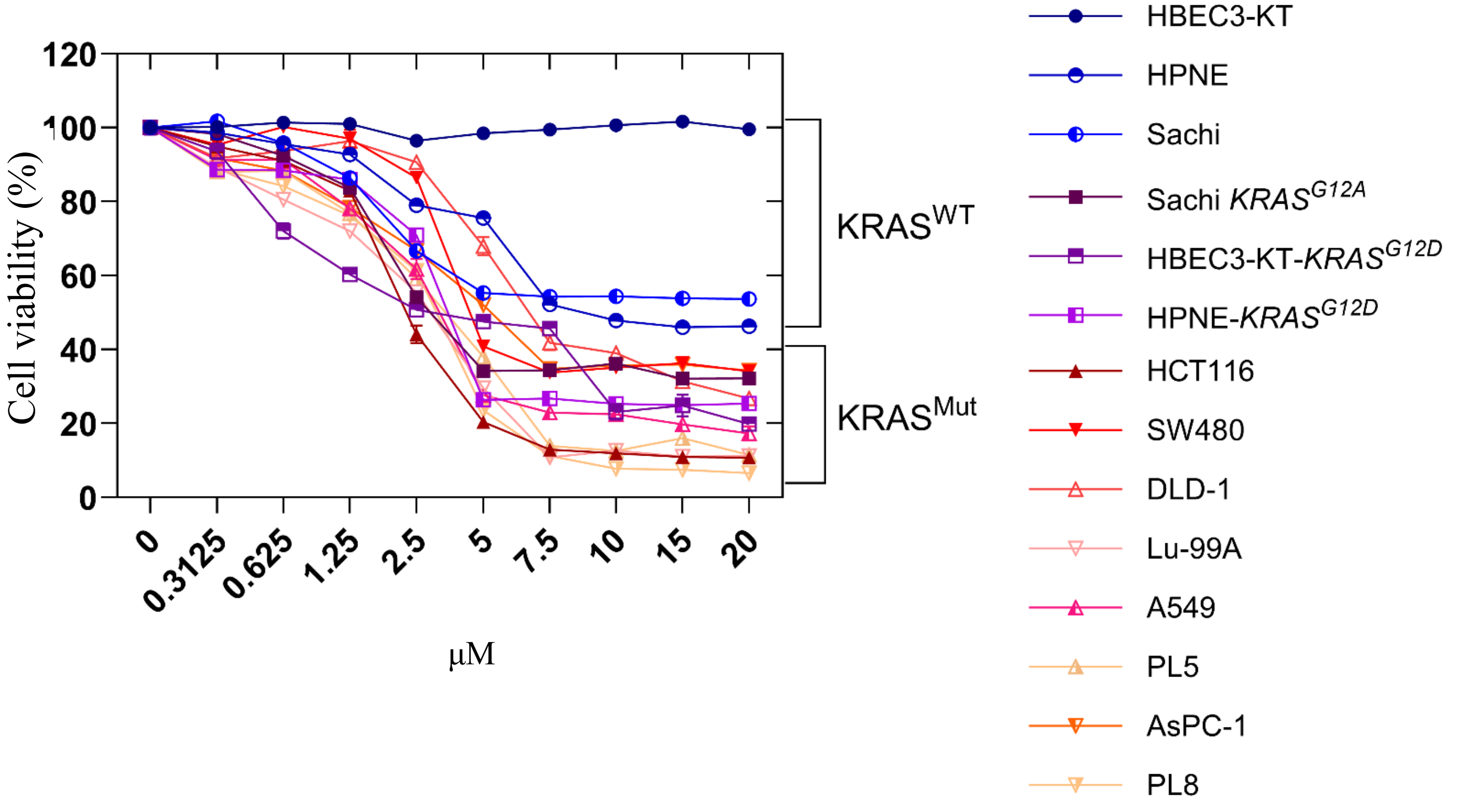

Fig. S4. Nintedanib Selectively Suppresses the Proliferation of KRAS-Mutant Cells. To evaluate the impact of Nintedanib on the proliferation of *KRAS* mutant cell lines, we conducted MTT assays using various *KRAS* mutant cell lines. These included HBEC3-KT, HPNE, and Sachi (*KRAS* wild-type cell lines; black lines) as well as Sachi-*KRAS*^{G12D}, HBEC3-KT-*KRAS*^{G12D}, HCT116, SW480, DLD1, Lu-99A, A549, PL5, AsPC-1, and PL8 (KRAS mutant cell lines; red lines). Interestingly, the average IC50 values in the *KRAS* mutant cell lines ranged from 2μM to 7.5 μM, which is lower than the IC_{50} value in the *KRAS* wild-type cell lines (approximately 20 μM).

Fig. S5

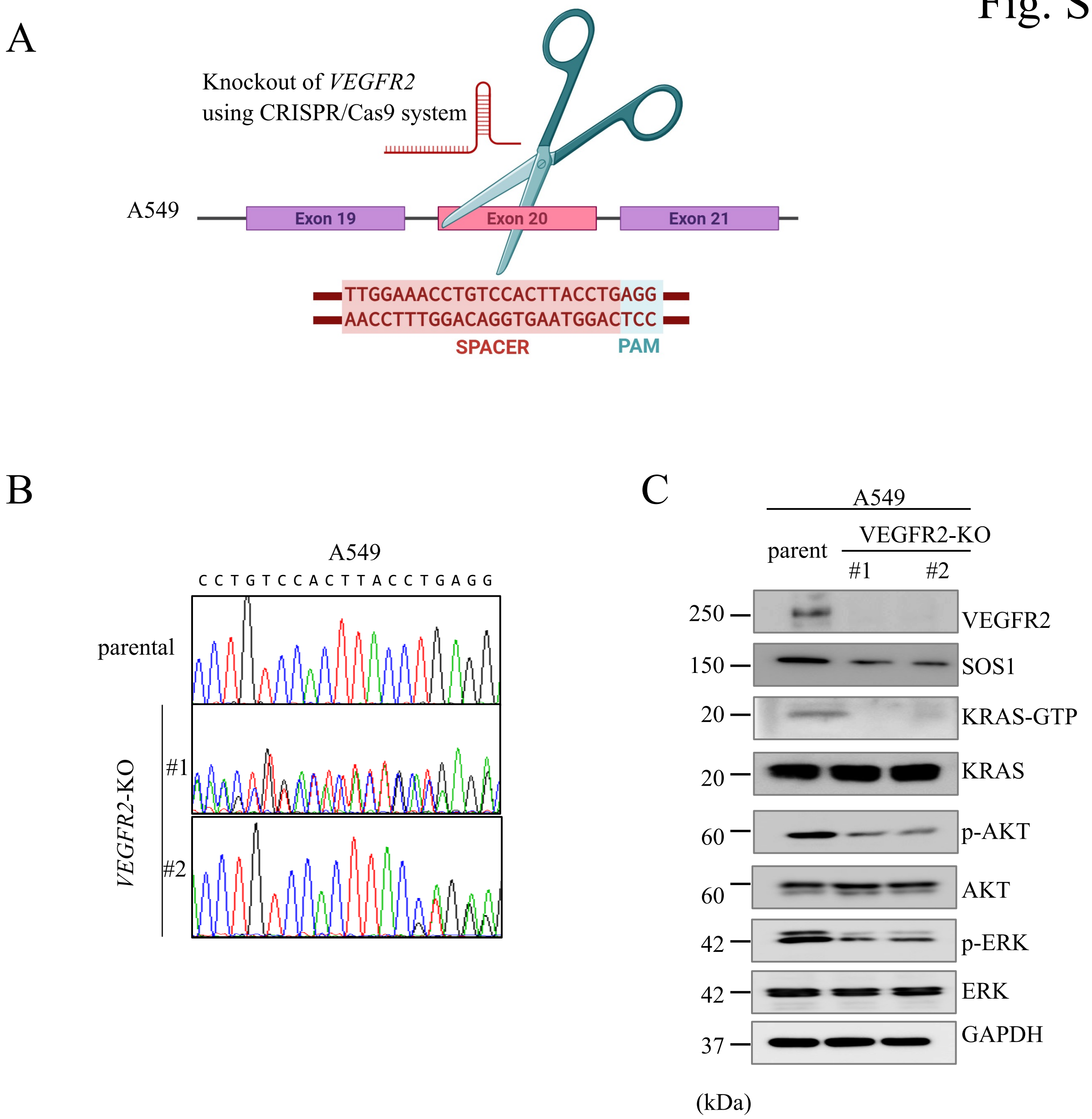

Fig. S5. Effect of KRAS-GTP on A549-VEGFR2-KO cells.
Generation of VEGFR2-knockout (VEGFR2-KO) cell clones using the CRISPR/Cas9 system in the human A549 cell line.**(A)** A single-guide RNA (sgRNA) sequence was designed against exon 20 of the VEGFR2 locus.**(B)** Genomic sequence analysis of VEGFR2 in A549 cells was compared with that of the parental cells.**(C)** Protein expression levels of SOS1, KRas-GTP, total KRAS, pAKT, AKT, pERK, ERK, and GAPDH were analyzed by western blotting in A549 cells.

Fig. S6

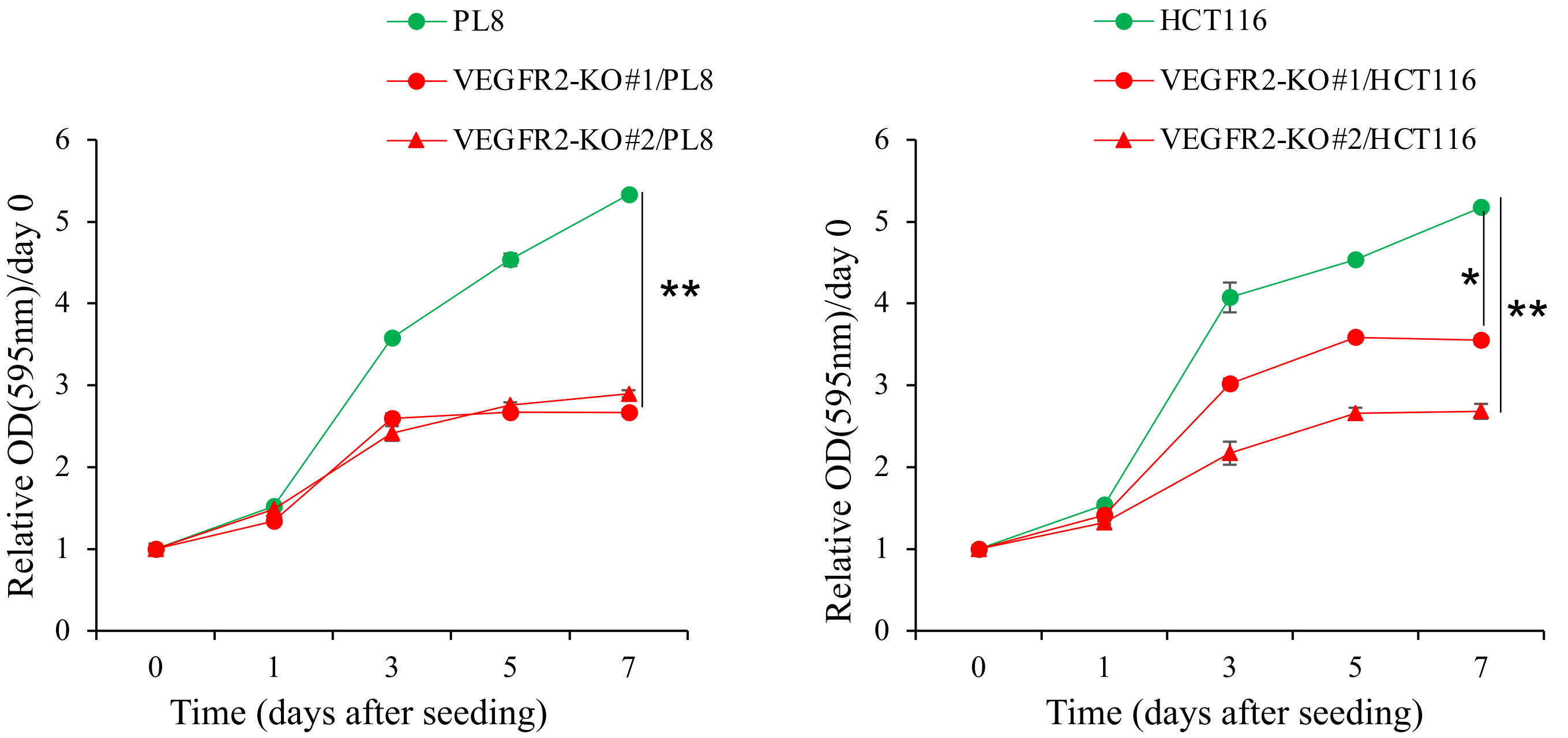

Fig. S6 Cell growth assay of VEGFR2-KO inPL8 and HCT16 cells.. MTT-based proliferation assay of parental PL8 and HCT116 cells, VEGFR2-KO/PL8 cell (#1 and #2), and VEGFR2-KO/HCT116 cell (#1 and #2), The relative OD at 595 nm was calculated by dividing the OD of day 0 at each time point (0, 1, 3, 5, and 7) and is expressed as mean ± SEM (n = 3). 、*$p < 0.05$, **$p < 0.001$

Fig. S7

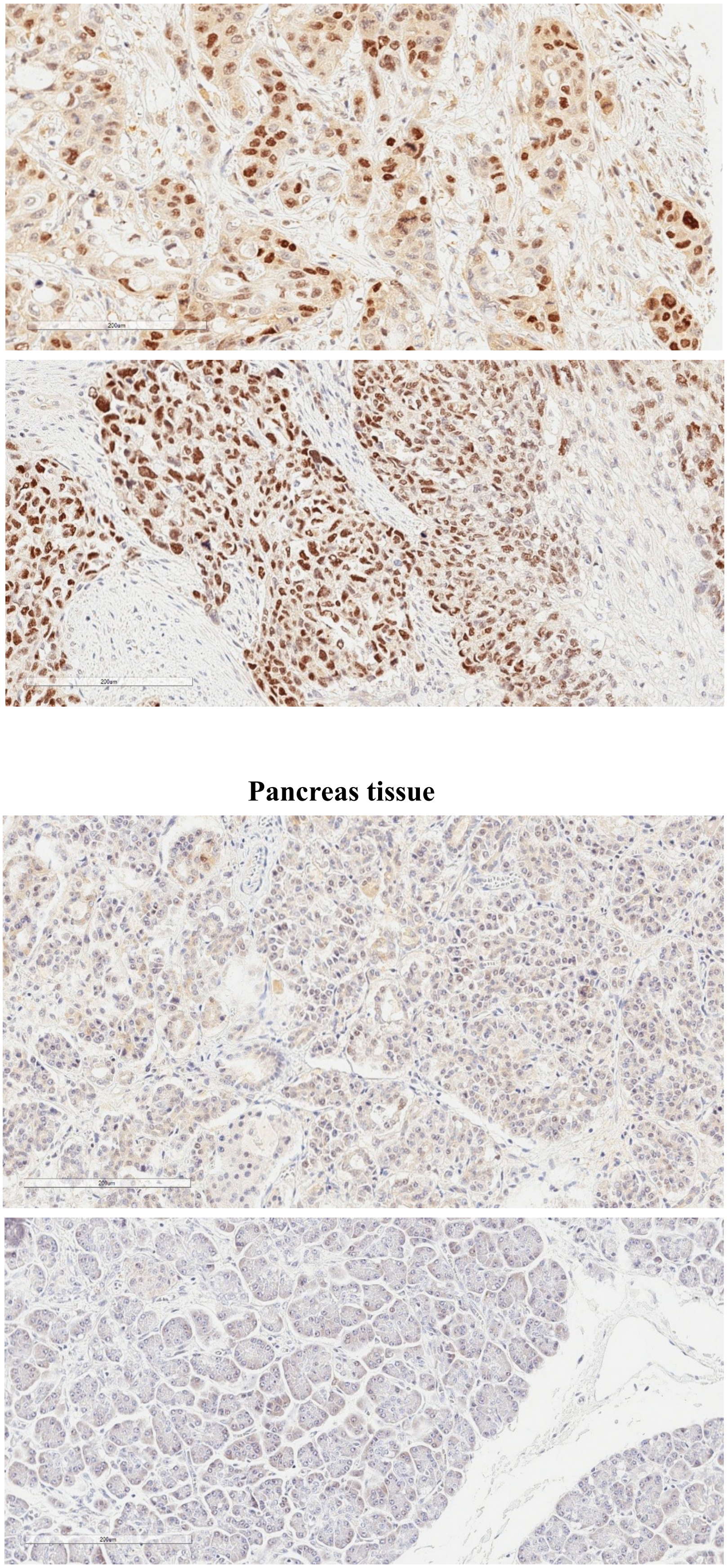

Fig. S7. To investigate the mutation status of p53 in human pancreatic cancer tissues, we conducted immunohistochemical analysis on 80 human pancreatic cancer tissues and 20 normal pancreatic tissues. In microscopic analysis, p53 positive signals were detected in 22 out of 80 pancreatic cancer tissues, while no signals were observed in the 20 normal pancreatic tissues (Table 1).
