# Supplementary material for: Nintedanib inhibits the VEGFR–ERK signaling pathway in human KRAS-mutated cancer cells
Source: Cell Death Dis. 2026 Jun 23;17(1):780. doi: 10.1038/s41419-026-09015-2 (PMC13547290; doi:10.1038/s41419-026-09015-2)
Supplement: Supplementary file 2 [file 41419_2026_9015_MOESM2_ESM.pdf]

Table S1. Upregulated genes in HPNE *KRAS*^{G12D} cells

**Gene Name**	**Description**	**Fold Change *KRAS*^{G12D} VS parental**
MAGEA2B	MAGE family member A2B	420.5
MAGEA6	MAGE family member A6	357.5
PAGE1	PAGE family member 1	149.8
MAGEA12	MAGE family member A12	98.5
MAGEA1	MAGE family member A1	74.5
SAG1	chondrosarcoma associated gene 1	57.7
CSAG4	CSAG family member 4 (pseudogene)	46.2
PECAM1	platelet and endothelial cell adhesion molecule 1	38.6
ST8SIA4	ST8 alpha-N-acetyl-neuraminide alpha-2,8-sialyltransferase 4	36.6
PRSS3	serine protease 3	28.2
FSTL5	follistatin like 5	27.0
PRSS3P2	PRSS3 pseudogene 2	26.8
PAGE2B	PAGE family member 2B	24.6
F2RL1	F2R like trypsin receptor 1	23.8
TMEM158	transmembrane protein 158	23.4
SLC16A8	solute carrier family 16 member 8	23.2
CDH6	cadherin 6	21.6
PLXDC2	plexin domain containing 2	21.4
PAGE2	PAGE family member 2	20.4
CDH9	cadherin 9	19.2
SLC6A15	solute carrier family 6 member 15	17.0
HSD11B1	hydroxysteroid 11-beta dehydrogenase 1	14.6
TMOD1	tropomodulin 1	13.4
MIAT	myocardial infarction associated transcript	13.1
TFPI2	tissue factor pathway inhibitor 2	12.9
SEMA3D	semaphorin 3D	12.8
BCHE	butyrylcholinesterase	12.4
PHACTR1	phosphatase and actin regulator 1	12.2
SEMA3A	semaphorin 3A	12.1
RGS2	regulator of G protein signaling 2	11.1

BRINP3	BMP/retinoic acid inducible neural specific 3	10.8
HS3ST1	heparan sulfate-glucosamine 3-sulfotransferase 1	10.8
SPRY1	sprouty RTK signaling antagonist 1	10.8
ABCA6	ATP binding cassette subfamily A member 6	10.4
TNNT1	troponin T1, slow skeletal type	9.7
ABCA8	ATP binding cassette subfamily A member 8	9.6
LRRC15	leucine rich repeat containing 15	9.4
VSNL1	visinin like 1	8.9
KCNJ6	potassium inwardly rectifying channel subfamily J member 6	8.7
SCG5	secretogranin V	8.4
AF132204	None	8.3
PCDH10	protocadherin 10	8.3
AKR1C3	aldo-keto reductase family 1 member C3	7.9
ACOX2	acyl-CoA oxidase 2	7.5
SERPINE2	serpin family E member 2	7.5
PDE7B	phosphodiesterase 7B	7.5
ITGA8	integrin subunit alpha 8	7.4
ARG2	arginase 2	7.2
STC1	stanniocalcin 1	7.1
NPTX1	neuronal pentraxin 1	7.0
TEX29	testis expressed 29	6.9
C12orf56	chromosome 12 open reading frame 56	6.8
CD33	CD33 molecule	6.6
ANKH	ANKH inorganic pyrophosphate transport regulator	6.6
STK38L	serine/threonine kinase 38 like	6.5
VEGFA	vascular endothelial growth factor A	6.5
KCNN4	potassium calcium-activated channel subfamily N member 4	6.4
TRO	trophinin	6.4
FPR3	formyl peptide receptor 3	6.4
FZD1	frizzled class receptor 1	6.4
VCAN	versican	6.4

TNFRSF11B	TNF receptor superfamily member 11b	6.3
SPDL1	spindle apparatus coiled-coil protein 1	6.2
SOD3	superoxide dismutase 3	6.2
SGCA	sarcoglycan alpha	6.2
ACSM5	acyl-CoA synthetase medium chain family member 5	6.1
ANTXR2	ANTXR cell adhesion molecule 2	6.0
AHNAK2	AHNAK nucleoprotein 2	6.0
HS3ST3A1	heparan sulfate-glucosamine 3-sulfotransferase 3A1	5.9
CEP170	centrosomal protein 170	5.9
PLEK2	pleckstrin 2	5.9
PRUNE2	prune homolog 2 with BCH domain	5.8
TRIML2	tripartite motif family like 2	5.8
TNC	tenascin C	5.8
JUP	junction plakoglobin	5.8
ANK3	ankyrin 3	5.7
PROS1	protein S	5.6
PTGS1	prostaglandin-endoperoxide synthase 1	5.6
DIO2	iodothyronine deiodinase 2	5.5
MAN1A1	mannosidase alpha class 1A member 1	5.5
EGR2	early growth response 2	5.5
SYT7	synaptotagmin 7	5.5
PLAU	plasminogen activator, urokinase	5.4
DPYSL3	dihydropyrimidinase like 3	5.4
SPOCK3	SPARC (osteonectin), cwcv and kazal like domains proteoglycan 3	5.4
NOX4	NADPH oxidase 4	5.4
H2AFV	H2A.Z variant histone 2	5.4
ATL1	atlastin GTPase 1	5.4
LRRC17	leucine rich repeat containing 17	5.4
WLS	Wnt ligand secretion mediator	5.4
RGS17	regulator of G protein signaling 17	5.4
CSGALNACT1	chondroitin sulfate N-acetylgalactosaminyltransferase 1	5.4
WDR66	cilia and flagella associated protein 251	5.3

ITGA2	integrin subunit alpha 2	5.3
AKAP12	A-kinase anchoring protein 12	5.3
SLC27A3	solute carrier family 27-member 3	5.3
GRK5	G protein-coupled receptor kinase 5	5.3
PSD3	pleckstrin and Sec7 domain containing 3	5.2
ZNF608	zinc finger protein 608	5.2
SPOCK1	SPARC (osteonectin), cwcv and kazal like domains proteoglycan 1	5.2
TMEM65	transmembrane protein 65	5.1
SFRP1	secreted frizzled related protein 1	5.1
FOXL2NB	FOXL2 neighbor	5.1
TMEM26	transmembrane protein 26	5.1
SVEP1	sushi, von Willebrand factor type A, EGF and pentraxin domain containing 1	5.0

Upregulated genes by KRAS^{G12D} indicated based on the differential expression (fold change) in *KRAS*^{G12D} cells versus parent cells (fold change>5)
