# Supplementary material for: Nintedanib inhibits the VEGFR–ERK signaling pathway in human KRAS-mutated cancer cells
Source: Cell Death Dis. 2026 Jun 23;17(1):780. doi: 10.1038/s41419-026-09015-2 (PMC13547290; doi:10.1038/s41419-026-09015-2)
Supplement: Supplementary file 3 [file 41419_2026_9015_MOESM3_ESM.pdf]

Table S2. Downregulated genes in HPNE *KRAS*^{G12D} cells

**Gene Name**	**Description**	**Fold change *KRAS*^{G12D} VS parental**
FBXO25	F-box protein 25	0.01
HAPLN1	hyaluronan and proteoglycan link protein 1	0.01
GSTM3	glutathione S-transferase mu 3	0.01
TIMP3	TIMP metallopeptidase inhibitor 3	0.01
FEZ1	fasciculation and elongation protein zeta 1	0.02
CDKN1C	cyclin dependent kinase inhibitor 1C	0.03
ALDH2	aldehyde dehydrogenase 2 family member	0.03
THY1	Thy-1 cell surface antigen	0.04
PTGFRN	prostaglandin F2 receptor inhibitor	0.05
KEAP1	kelch like ECH associated protein 1	0.07
HLA-DPB1	major histocompatibility complex, class II, DP beta 1	0.09
GOLGA5	golgin A5	0.1
KRTAP1-5	keratin associated protein 1-5	0.13
OPLAH	5-oxoprolinase, ATP-hydrolysing	0.14
EFS	embryonal Fyn-associated substrate	0.15
IFI27	interferon alpha inducible protein 27	0.15
PSG5	pregnancy specific beta-1-glycoprotein 5	0.16
GMFG	glia maturation factor gamma	0.16
CAPZB	capping actin protein of muscle Z-line subunit beta	0.17
MIEF1	mitochondrial elongation factor 1	0.18
LY6K	lymphocyte antigen 6 family member K	0.19

Downregulated genes by KRAS^{G12D} indicated based on the differential expression (fold change) in *KRAS*^{G12D} cells versus parent cells (fold change<0.2)
