# Supplementary material for: Nintedanib inhibits the VEGFR–ERK signaling pathway in human KRAS-mutated cancer cells
Source: Cell Death Dis. 2026 Jun 23;17(1):780. doi: 10.1038/s41419-026-09015-2 (PMC13547290; doi:10.1038/s41419-026-09015-2)
Supplement: Supplementary file 4 [file 41419_2026_9015_MOESM4_ESM.pdf]

Table S3. Effect of 1374 inhibitors on the proliferation of *KRAS* mutants and $KRAS^{WT}$ cells

No	Cat.No	Product Name	Target	Sachi (% cells)	Sachi-$KRAS^{G12A}$ (% cells)	HPNE (%cells)	HPNE-$KRAS^{G12D}$ (% cells)
1	S1003	Linifanib (ABT-869)	CSF-1R, PDGFR, VEGFR	95.2	101.9	99.4	86.8
2	S1415	Clopidogrel	P2 Receptor	88.2	92.5	108.4	96.6
3	S1013	Bortezomib (PS-341)	Proteasome	12.9	9.6	84.8	47.7
4	S1438	Topiramate	Carbonic Anhydrase	73.1	93.5	114.2	95.3
5	S1033	Nilotinib (AMN-107)	Bcr-Abl	89.7	92.8	106.8	76.8
6	S1456	Zibotentan (ZD4054)	Endothelin Receptor	88.2	82.7	116.9	88.4
7	S1053	Entinostat (MS-275)	HDAC	26.3	20.7	113.3	81.0
8	S1475	Pimasertib (AS-703026)	MEK	54.2	70.2	99.7	98.5
9	S1087	Iniparib (BSI-201)	PARP	26.2	14.9	110.8	108.6
10	S1497	Pralatrexate	DHFR	28.2	19.3	149.5	87.1
11	S1119	Cabozantinib (XL184, BMS-907351)	c-Met, VEGFR	26.6	19.0	126.0	91.4
12	S1514	Cyclosporine	phosphatase, Immunology & Inflammation related	13.3	9.7	45.6	34.9
13	S1147	Barasertib (AZD1152-HQPA)	Aurora Kinase	24.0	18.1	89.0	61.2
14	S1549	Nebivolol HCl	Adrenergic Receptor	15.1	10.9	34.8	21.9
15	S1164	Lenvatinib (E7080)	VEGFR	24.6	18.5	104.9	80.2
16	S1578	Candesartan	RAAS	29.0	20.2	127.7	92.0
17	S1194	CUDC-101	EGFR, HDAC, HER2	27.7	20.2	96.6	66.2
18	S1607	Rizatriptan Benzoate	5-HT Receptor	108.6	105.0	107.6	93.5
19	S1204	Melatonin	MT Receptor	103.8	104.0	110.2	96.6
20	S1620	Darunavir Ethanolate	HIV Protease	30.1	19.5	108.8	74.2
21	S1218	Clofarabine	DNA/RNA Synthesis	19.5	12.5	95.2	137.0
22	S1630	Allopurinol	ROS	92.0	99.4	114.5	83.1
23	S1230	Flavopiridol (Alvocidib)	CDK	23.0	15.0	101.5	89.8
24	S1641	Chlorothiazide	Others	98.2	95.5	110.1	84.6
25	S1252	Entecavir Hydrate	Reverse Transcriptase	58.4	42.3	99.0	74.7
26	S1652	Monobenzone	Tyrosinase	44.8	38.8	114.8	92.0

27	S1262	Avagacestat (BMS-708163)	Beta Amyloid, Gamma-secretase	88.9	99.9	110.9	97.4
28	S1666	Flucytosine	Anti-infection	93.0	100.0	113.6	86.0
29	S1283	Asenapine maleate	5-HT Receptor, Adrenergic Receptor	50.7	68.5	89.4	65.6
30	S1681	Mesalamine	IκB/IKK, Immunology & Inflammation related	30.1	23.3	109.7	84.3
31	S1299	Floxuridine	DNA/RNA Synthesis	15.5	10.9	88.6	59.4
32	S1693	Carbamazepine	Sodium Channel,Autophagy	27.2	19.0	109.4	86.5
33	S1326	Edaravone	Others	24.0	16.6	110.5	87.4
34	S1709	Estradiol	Estrogen/progestogen Receptor	44.8	32.2	117.2	83.1
35	S1336	Fluvoxamine maleate	5-HT Receptor	36.7	23.4	104.9	66.7
36	S1721	Azathioprine	Rho	36.2	25.8	91.3	65.8
37	S1358	Loratadine	Histamine Receptor	95.3	80.4	93.5	80.1
38	S1734	Meloxicam	COX	116.7	106.3	115.4	81.0
39	S1374	Doripenem Hydrate	Anti-infection	115.8	107.3	112.1	77.4
40	S1742	Nevirapine	Reverse Transcriptase	82.8	88.4	115.6	83.6
41	S1386	Nafamostat Mesylate	Serine Protease	106.5	110.1	43.4	34.3
42	S1760	Rifapentine	DNA/RNA Synthesis	80.7	94.7	137.7	90.9
43	S1398	Stavudine (d4T)	Reverse Transcriptase	90.2	77.1	102.8	72.0
44	S1773	Oxytetracycline (Terramycin)	Anti-infection	44.8	97.1	88.5	56.9
45	S1004	Veliparib (ABT-888)	PARP	92.4	89.8	102.7	83.9
46	S1425	Ranolazine 2HCl	Calcium Channel	88.8	108.3	103.2	77.6
47	S1017	Cediranib (AZD2171)	VEGFR	45.6	16.4	73.5	46.8
48	S1439	Tranilast	Immunology & Inflammation related	62.9	73.7	108.7	77.3
49	S1035	Pazopanib HCl (GW786034 HCl)	c-Kit,PDGFR,VEGFR	71.9	90.4	85.7	60.0
50	S1457	Atazanavir Sulfate	HIV Protease	100.6	101.3	107.1	77.2
51	S1055	Enzastaurin (LY317615)	PKC	94.9	103.3	106.7	80.5
52	S1480	Lomibuvir (VX-222, VCH-222)	HCV Protease	11.6	17.7	101.0	80.5
53	S1090	Abexinostat (PCI-24781)	HDAC	26.5	20.9	90.9	64.1

54	S1500	Betamethasone	Glucocorticoid Receptor	45.5	77.8	104.4	93.8
55	S1120	Everolimus (RAD001)	mTOR	24.6	30.4	80.7	75.5
56	S1515	Pracinostat (SB939)	HDAC	26.0	19.7	93.4	63.8
57	S1148	Docetaxel	Microtubule Associated	31.8	20.3	94.8	67.9
58	S1550	Pimobendan	PDE	88.5	98.8	97.1	72.9
59	S1168	Valproic acid sodium salt (Sodium valproate)	GABA Receptor,HDAC,Autophagy	109.3	108.2	90.5	74.5
60	S1593	Apixaban	Factor Xa	107.7	105.9	103.7	74.3
61	S1195	TAK-700 (Orteronel)	P450 (e.g. CYP17)	105.2	107.8	98.9	74.2
62	S1608	Pyridostigmine Bromide	AChR	106.9	106.8	103.0	156.6
63	S1206	Bisoprolol fumarate	Adrenergic Receptor	104.2	105.0	105.5	79.7
64	S1622	Prednisone	Glucocorticoid Receptor,Immunology & Inflammation related	111.1	113.6	114.5	78.4
65	S1221	Dacarbazine	DNA/RNA Synthesis	51.3	59.0	73.9	80.6
66	S1631	Allopurinol Sodium	ROS	92.9	97.0	99.9	73.0
67	S1231	Topotecan HCl	Topoisomerase	23.8	16.4	112.7	77.3
68	S1643	Ursodiol	Others	94.1	100.0	103.3	76.0
69	S1255	Nepafenac	COX	97.9	91.7	103.3	88.4
70	S1653	Tretinoin	Retinoid Receptor	34.4	69.3	99.1	91.9
71	S1267	Vemurafenib (PLX4032, RG7204)	Raf	38.2	52.2	101.5	71.1
72	S1667	Trichlormethiazide	Others	81.6	91.8	106.4	87.7
73	S1284	Benazepril HCl	RAAS	106.7	106.5	99.8	85.5
74	S1683	Ipratropium Bromide	AChR	66.3	87.4	97.9	74.0
75	S1300	Tegafur (FT-207, NSC 148958)	DNA/RNA Synthesis	111.5	98.2	112.0	77.4
76	S1696	Hydrocortisone	Glucocorticoid Receptor	55.0	75.3	115.5	88.2
77	S1328	Etodolac	COX	35.6	44.9	110.6	79.2
78	S1712	Deferasirox	P450 (e.g. CYP17)	23.5	15.7	114.8	80.3
79	S1340	Gatifloxacin	Topoisomerase	12.7	9.1	65.5	40.1
80	S1723	Indomethacin	COX	95.1	104.2	105.8	76.8
81	S1359	Losartan Potassium (DuP 753)	RAAS	113.1	106.0	96.2	77.1
82	S1735	Mesna	Others	108.7	103.5	105.7	77.2

83	S1376	Gestodene	Estrogen/progestogen Receptor	107.7	100.6	100.7	75.9
84	S1743	NEXIUM (esomeprazole magnesium)	Proton Pump	99.6	104.7	92.8	82.7
85	S1387	Naftopidil DiHCl	Adrenergic Receptor	76.7	82.6	100.3	64.4
86	S1761	Suprofen	COX	108.8	111.1	115.3	73.4
87	S1400	Tenofovir Disoproxil Fumarate	Reverse Transcriptase	61.2	56.3	107.9	69.4
88	S1774	Thioguanine	DNA Methyltransferase	26.9	17.3	69.2	44.4
89	S1005	Axitinib	c-Kit, PDGFR, VEGFR	55.0	28.7	91.0	72.1
90	S1426	Repaglinide	Potassium Channel	105.6	107.5	104.3	95.3
91	S1018	Dovitinib (TKI-258, CHIR-258)	cKit, FGFR, FLT3, PDGFR, VEGFR	42.1	18.3	56.8	24.0
92	S1441	Venlafaxine HCl	5-HT Receptor	75.7	79.2	177.4	92.8
93	S1039	Rapamycin (Sirolimus)	Autophagy, Tor	29.7	38.9	85.3	90.8
94	S1465	Moxifloxacin HCl	Topoisomerase	59.0	74.3	56.2	52.5
95	S1060	Olaparib (AZD2281, Ku-0059436)	PARP	68.9	59.5	108.7	89.8
96	S1481	Zosuquidar (LY335979) 3HCl	P-gp	12.0	8.7	30.6	19.5
97	S1091	Linsitinib (OSI-906)	IGF-1R	24.1	22.6	79.8	58.2
98	S1501	Mycophenolate Mofetil	Dehydrogenase	21.7	16.9	107.4	73.9
99	S1122	Mocetinostat (MGCD0103)	HDAC	23.4	16.9	113.5	81.2
100	S1517	Natamycin	Anti-infection	87.7	95.6	105.8	79.2
101	S1150	Paclitaxel	Autophagy, Microtubule Associated	31.7	21.4	92.3	71.5
102	S1565	VX-809 (Lumacaftor)	CFTR	105.8	100.7	112.6	72.1
103	S1178	Regorafenib (BAY 73-4506)	c-RET, VEGFR	27.1	19.8	91.4	67.0
104	S1594	Semagacestat (LY450139)	Gamma-secretase	110.6	105.2	102.0	79.9
105	S1196	Exemestane	Aromatase	109.9	75.6	98.8	95.6
106	S1610	Metolazone	Others	94.3	108.1	102.6	103.5
107	S1207	Tivozanib (AV-951)	c-Kit,PDGFR,VEGFR	36.0	58.1	76.1	70.9
108	S1623	Acetylcysteine	TNF-alpha,ROS	102.5	108.6	107.4	75.3

109	S1222	Dexrazoxane HCl (ICRF-187, ADR-529)	Topoisomerase	90.0	102.3	104.0	89.1
110	S1633	Zafirlukast	LTR	90.7	96.4	106.6	76.9
111	S1237	Temozolomide	DNA/RNA Synthesis,Autophagy	59.6	60.9	109.9	76.7
112	S1644	Nitrofural	Anti-infection	82.3	80.2	96.4	86.6
113	S1256	Rufinamide	Sodium Channel	87.0	74.4	107.4	87.7
114	S1654	Phenylbutazone	Immunology & Inflammation related	33.4	25.6	110.3	85.3
115	S1271	Acarbose	Others	93.5	105.2	99.6	82.5
116	S1669	Loteprednol etabonate	Glucocorticoid Receptor	63.1	86.5	119.3	92.7
117	S1286	Budesonide	Glucocorticoid Receptor	50.8	90.5	126.5	105.7
118	S1685	Sulfanilamide	Anti-infection	60.5	71.9	103.0	88.4
119	S1302	Ifosfamide	DNA/RNA Synthesis	26.0	19.9	114.8	82.1
120	S1701	Desonide	Glucocorticoid Receptor	45.3	72.4	114.6	81.9
121	S1329	Etomidate	GABA Receptor	15.6	12.8	102.9	79.3
122	S1713	Piroxicam	COX	86.1	98.4	117.2	77.7
123	S1342	Genistein	EGFR,Topoisomerase	39.1	42.4	93.5	78.0
124	S1725	Terbinafine	Anti-infection	88.0	87.1	105.3	77.1
125	S1362	Rigosertib (ON-01910)	PLK	22.7	16.7	87.2	70.5
126	S1736	Methocarbamol	Carbonic Anhydrase	105.0	108.9	111.7	77.3
127	S1377	Drospirenone	Estrogen/progestogen Receptor	75.9	87.1	96.8	74.6
128	S1744	Nicotinic Acid	Vitamin	104.4	113.8	102.8	94.3
129	S1389	Omeprazole	Proton Pump,Autophagy	111.7	108.4	93.7	69.8
130	S1762	Pyrazinamide	Anti-infection	105.2	103.1	108.2	73.1
131	S1401	Tenofovir	Reverse Transcriptase	111.4	113.6	102.4	73.5
132	S1775	Thiotepa	Others	50.0	30.7	113.9	75.2
133	S1006	Saracatinib (AZD0530)	Src	61.3	84.2	77.7	56.2
134	S1430	Rolipram	PDE	101.8	102.3	99.3	78.9
135	S1021	Dasatinib	Bcr-Abl,c-Kit,Src	32.4	16.9	85.5	58.5
136	S1442	Voriconazole	P450 (e.g. CYP17)	55.6	62.1	99.8	74.9
137	S1040	Sorafenib Tosylate	PDGFR,Raf,VEGFR	105.8	108.8	107.5	80.5
138	S1467	Doxercalciferol	Vitamin	18.5	14.5	103.9	85.6
139	S1064	Masitinib (AB1010)	c-Kit,PDGFR	30.8	29.5	65.2	49.9
140	S1482	Daclatasvir (BMS-790052)	HCV Protease	17.5	13.3	35.5	20.5

141	S1096	Quisinostat (JNJ-26481585) 2HCl	HDAC	23.0	16.5	95.7	63.3
142	S1504	Dyphylline	PDE	64.3	86.0	109.3	84.1
143	S1130	YM155 (Sepantronium Bromide)	Survivin	13.3	10.4	32.2	19.6
144	S1523	Voxtalisib (SAR245409, XL765) Analogue	mTOR, PI3K	47.7	26.5	127.9	97.0
145	S1153	Roscovitine (Seliciclib, CYC202)	CDK	23.8	14.4	95.5	72.9
146	S1567	Pomalidomide	TNF-alpha	72.9	86.9	109.6	77.7
147	S1185	Ritonavir	HIV Protease, P450 (e.g. CYP17)	29.7	19.4	69.2	68.8
148	S1601	Reserpine	Others	88.7	71.4	104.8	66.0
149	S1197	Finasteride	5-alpha Reductase	99.9	102.2	100.6	78.4
150	S1611	Cefoperazone	Anti-infection	121.5	118.2	108.9	86.0
151	S1208	Doxorubicin (Adriamycin) HCl	Topoisomerase	14.4	10.9	52.3	35.0
152	S1625	Ethinyl Estradiol	Ethinyl Estradiol	23.2	27.6	108.8	71.4
153	S1223	Epirubicin HCl	Topoisomerase	15.0	11.1	51.3	36.4
154	S1635	Erythromycin	Anti-infection	96.8	109.7	114.2	76.7
155	S1238	Tamoxifen	Estrogen/progestogen Receptor,Autophagy	57.2	37.9	48.7	28.7
156	S1645	Ketoprofen	COX	96.7	102.4	106.0	80.9
157	S1257	Posaconazole	P450 (e.g. CYP17)	101.6	97.5	107.1	86.7
158	S1655	Ezetimibe	LDL	80.8	95.8	106.8	89.0
159	S1276	Adapalene	Retinoid Receptor	47.7	32.9	91.5	69.2
160	S1672	Aminoglutethimide	Aromatase	112.0	110.3	108.8	81.5
161	S1287	Bumetanide	Others	101.5	103.1	104.1	89.0
162	S1688	Betamethasone Dipropionate	Glucocorticoid Receptor	48.5	73.4	115.6	93.5
163	S1304	Megestrol Acetate	Androgen Receptor,Estrogen/progestogen Receptor	27.8	20.6	104.7	84.2
164	S1702	Didanosine	Reverse Transcriptase	55.6	75.1	109.7	81.4
165	S1330	Felbamate	NMDAR	15.9	11.8	107.4	82.8

166	S1714	Gemcitabine	Autophagy,DNA/RNA Synthesis	13.1	9.1	84.6	64.0
167	S1344	Glimepiride	Potassium Channel	74.7	78.6	90.7	57.3
168	S1727	Levonorgestrel	Estrogen/progestogen Receptor	100.5	107.9	101.3	76.7
169	S1364	Patupilone (EPO906, Epothilone B)	Microtubule Associated	31.2	20.7	85.0	62.1
170	S1737	Prednisolone	Immunology & Inflammation related,Glucocorticoid Receptor	65.2	82.9	103.4	73.3
171	S1379	Isotretinoin	Hydroxylase	77.2	86.7	103.0	75.0
172	S1747	Nimodipine	Autophagy,Calcium Channel	89.3	102.7	92.0	76.3
173	S1390	Ondansetron HCl	5-HT Receptor	107.1	102.7	98.6	79.2
174	S1763	Quetiapine Fumarate	Dopamine Receptor	59.0	77.0	90.3	62.0
175	S1403	Tigecycline	Anti-infection	50.3	69.1	95.3	65.7
176	S1776	Toremifene Citrate	Estrogen/progestogen Receptor	12.4	9.5	38.7	25.1
177	S1007	Roxadustat (FG-4592)	HIF	33.9	21.0	99.6	71.4
178	S1431	Sildenafil Citrate	PDE	92.3	98.9	98.3	75.8
179	S1025	Gefitinib (ZD1839)	EGFR	87.9	52.2	83.5	57.4
180	S1443	Zileuton	Lipoxygenase	87.4	84.8	99.8	83.7
181	S1042	Sunitinib Malate	c-Kit,PDGFR,VEGFR	14.5	11.0	53.9	37.5
182	S1468	Alfacalcidol	Vitamin	18.0	14.1	104.3	101.7
183	S1065	Pictilisib (GDC-0941)	PI3K	41.7	50.7	99.3	86.5
184	S1483	Iloperidone	5-HT Receptor	35.2	59.2	86.1	72.4
185	S1098	Rucaparib (AG-014699, PF-01367338) phosphate	PARP	22.4	20.2	105.3	75.6
186	S1505	Aztreonam	Anti-infection	71.0	90.8	103.1	73.4
187	S1133	Alisertib (MLN8237)	Aurora Kinase	37.1	20.3	100.5	76.6
188	S1526	Quizartinib (AC220)	FLT3	18.5	12.2	112.1	74.9
189	S1156	Capecitabine	DNA/RNA Synthesis	95.8	105.6	103.5	74.8
190	S1569	Tazarotene	Retinoid Receptor	64.8	81.5	87.9	80.3
191	S1189	Aprepitant	Substance P	27.4	19.1	89.6	67.3
192	S1603	Furosemide	Sodium Channel	111.3	108.8	99.9	79.7
193	S1199	Cladribine	DNA/RNA Synthesis	27.2	20.3	68.6	53.7
194	S1613	Silodosin	Adrenergic Receptor	96.4	114.7	104.9	75.1

195	S1209	Fluorouracil (5-Fluoracil, 5-FU)	DNA/RNA Synthesis	54.5	36.7	79.0	62.7
196	S1626	Naproxen Sodium	COX	113.5	108.5	103.6	72.9
197	S1225	Etoposide	Topoisomerase	20.4	13.6	101.0	81.8
198	S1636	Amphotericin B	Anti-infection	90.0	107.3	95.0	75.3
199	S1241	Vincristine sulfate	Autophagy,Microtubule Associated	24.1	14.8	99.9	71.7
200	S1646	Ketorolac	COX	96.1	92.4	116.4	74.1
201	S1258	Prasugrel	P2 Receptor	108.6	91.8	109.3	79.7
202	S1657	Enalaprilat Dihydrate	RAAS	95.3	112.7	100.1	96.4
203	S1278	Altretamine	DNA alkylator	44.3	62.3	105.8	78.2
204	S1676	Amorolfine HCl	Anti-infection	51.5	54.8	101.6	87.1
205	S1289	Carmofur	DNA/RNA Synthesis	50.3	33.8	82.5	60.9
206	S1689	Meprednisone	Glucocorticoid Receptor	39.6	48.3	96.7	83.6
207	S1305	Mercaptopurine (6-MP)	DNA/RNA Synthesis	30.5	23.8	108.1	88.8
208	S1703	Divalproex Sodium	HDAC	58.4	59.0	114.4	88.1
209	S1331	Fluconazole	P450 (e.g. CYP17)	95.4	83.7	99.6	83.2
210	S1715	Glipizide	Others	94.0	90.4	114.6	80.0
211	S1351	Ivermectin	Chloride Channel,Anti-infection	11.8	8.7	29.5	18.9
212	S1729	Gemfibrozil	PPAR	90.0	99.6	94.6	78.0
213	S1367	Amonafide	Topoisomerase	15.7	14.7	73.8	57.5
214	S1738	Telmisartan	RAAS	105.5	102.3	115.9	83.9
215	S1380	Lopinavir	HIV Protease	83.7	78.8	96.6	80.0
216	S1748	Nisoldipine	Calcium Channel	24.0	15.0	93.3	72.7
217	S1391	Oxcarbazepine	Sodium Channel	109.0	93.7	104.7	83.2
218	S1764	Rifampin	DNA/RNA Synthesis	111.7	116.6	124.1	90.0
219	S1405	Vecuronium Bromide	AChR	101.9	109.2	99.0	73.3
220	S1777	Ethionamide	Anti-infection	103.6	107.2	113.2	74.8
221	S1008	Selumetinib (AZD6244)	MEK	35.9	23.0	101.0	84.4
222	S1432	Sumatriptan Succinate	5-HT Receptor	99.7	99.2	99.6	74.8
223	S1026	Imatinib Mesylate (STI571)	Bcr-Abl,c-Kit,PDGFR	59.7	30.1	93.7	63.8
224	S1444	Ziprasidone HCl	5-HT Receptor,Dopamine Receptor	66.3	78.0	96.2	94.1

225	S1044	Temsirolimus (CCI-779, NSC 683864)	mTOR	33.6	48.0	58.8	35.4
226	S1469	Calcifediol	Vitamin	24.4	17.5	102.8	79.2
227	S1068	Crizotinib (PF-02341066)	ALK,c-Met	20.9	15.1	50.7	37.6
228	S1488	Naratriptan HCl	5-HT Receptor	74.4	96.0	101.5	75.8
229	S1101	Vatalanib (PTK787) 2HCl	VEGFR	32.8	29.7	75.2	61.0
230	S1508	Alprostadil	Immunology & Inflammation related	26.8	22.8	111.1	88.9
231	S1137	Malotilate	Others	67.1	82.5	110.7	89.4
232	S1538	Telaprevir (VX-950)	HCV Protease	35.6	34.2	99.7	102.1
233	S1157	Delanzomib (CEP-18770)	Proteasome	11.6	9.1	76.7	50.0
234	S1573	Fasudil (HA-1077) HCl	Autophagy,ROCK	80.1	73.5	107.0	92.5
235	S1191	Fulvestrant	Estrogen/progestogen Receptor	81.4	89.6	93.8	77.6
236	S1604	Olmesartan Medoxomil	RAAS	103.3	100.1	107.2	76.4
237	S1200	Decitabine	DNA Methyltransferase	51.2	35.4	103.8	92.4
238	S1614	Riluzole	GluR,Sodium Channel	43.6	43.7	100.5	75.7
239	S1210	Methotrexate	DHFR	25.1	17.7	105.3	81.2
240	S1627	Nitazoxanide	Others	77.6	92.7	134.2	122.0
241	S1227	Raloxifene HCl	Estrogen/progestogen Receptor	37.4	73.8	85.8	80.1
242	S1638	Ibuprofen	COX	103.9	111.8	106.4	80.8
243	S1243	Agomelatine	5-HT Receptor	90.6	99.7	101.6	76.9
244	S1647	Adenosine	Adenosine Receptor	84.4	82.5	102.2	77.7
245	S1259	Ramelteon	MT Receptor	97.5	97.1	99.5	75.5
246	S1658	Dofetilide	Potassium Channel	77.6	94.3	110.2	151.8
247	S1280	Amisulpride	Dopamine Receptor	92.7	100.7	101.7	76.3
248	S1677	Chloramphenicol	Anti-infection	106.3	103.3	71.2	59.4
249	S1291	Cetirizine DiHCl	Histamine Receptor	102.8	101.0	105.0	84.4
250	S1690	Betamethasone Valerate	Glucocorticoid Receptor	29.6	65.2	111.9	79.7
251	S1312	Streptozotocin (STZ)	DNA alkylator	91.9	98.1	110.8	82.7
252	S1704	Emtricitabine	Reverse Transcriptase	52.5	53.6	117.6	81.8
253	S1332	Flumazenil	GABA Receptor	52.3	42.7	105.6	84.9
254	S1716	Glyburide (Glibenclamide)	Potassium Channel	92.0	103.2	107.4	91.7
255	S1354	Lansoprazole	Proton Pump	69.8	86.5	89.9	66.6
256	S1730	Indapamide	Others	96.3	93.4	104.8	81.8
257	S1368	Acitretin	Retinoid Receptor	88.7	95.8	92.2	79.7

258	S1739	Thiabendazole	P450 (e.g. CYP17)	97.7	98.0	111.0	79.9
259	S1381	Meropenem	Anti-infection	112.2	104.7	104.2	87.1
260	S1754	Oxybutynin	AChR	55.1	72.5	99.0	67.8
261	S1394	Pizotifen Malate	5-HT Receptor	45.7	61.3	80.6	65.0
262	S1768	Cefditoren Pivoxil	Anti-infection	111.4	108.6	108.5	72.6
263	S1407	Bimatoprost	Immunology & Inflammation related	99.2	102.2	107.6	84.5
264	S1778	Trifluridine	DNA/RNA Synthesis	22.5	13.9	107.2	68.8
265	S1010	Nintedanib (BIBF 1120)	FGFR, PDGFR, VEGFR	91.4	53.2	46.4	21.3
266	S1436	Tianeptine sodium	5-HT Receptor	98.5	95.9	95.2	68.4
267	S1028	Lapatinib (GW-572016) Ditosylate	EGFR, HER2	34.8	11.1	81.9	57.5
268	S1445	Zonisamide	Sodium Channel	72.9	85.0	104.5	121.6
269	S1047	Vorinostat (SAHA, MK0683)	Autophagy, HDAC	26.6	19.5	99.6	62.7
270	S1470	Orantinib (TSU-68, SU6668)	PDGFR	99.5	103.8	90.1	64.6
271	S1082	Vismodegib (GDC-0449)	Hedgehog/Smoothened	100.4	94.5	100.7	109.1
272	S1490	Ponatinib (AP24534)	Bcr-Abl,FGFR,PDGFR,VEGFR	20.3	15.2	44.5	37.6
273	S1111	Foretinib (GSK1363089)	c-Met,VEGFR	23.7	23.5	33.9	18.8
274	S1511	Lactulose	Others	48.9	57.4	104.5	75.2
275	S1141	Tanespimycin (17-AAG)	HSP (e.g. HSP90)	23.0	18.7	117.6	99.7
276	S1541	Selisistat (EX 527)	Sirtuin	54.0	68.6	112.4	75.5
277	S1159	Ganetespib (STA-9090)	HSP (e.g. HSP90)	20.9	17.2	99.9	84.7
278	S1574	Doramapimod (BIRB 796)	p38 MAPK	69.4	78.3	93.2	90.2
279	S1192	Raltitrexed	DNA/RNA Synthesis	24.1	19.0	100.7	72.0
280	S1605	Cefdinir	Anti-infection	105.6	96.3	109.4	89.0
281	S1201	Dimesna	Others	106.5	102.4	97.6	103.3
282	S1618	Sulfameter	DHFR	102.3	111.5	103.4	76.4
283	S1212	Bendamustine HCl	DNA/RNA Synthesis	112.0	105.5	101.0	81.9
284	S1628	Triamcinolone Acetonide	Glucocorticoid Receptor	58.6	91.0	115.3	84.1
285	S1228	Idarubicin HCl	Topoisomerase	12.4	8.8	43.7	24.5
286	S1639	Amprenavir	HIV Protease	60.9	68.1	107.1	71.0
287	S1244	Amuvatinib (MP-470)	c-Kit, FLT3, PDGFR	77.7	93.3	108.8	78.7

288	S1649	Zolmitriptan	5-HT Receptor	78.3	94.3	116.0	70.8
289	S1260	Cinacalcet HCl	CaSR	15.5	9.4	66.6	23.9
290	S1662	Isradipine	Calcium Channel	61.5	67.0	101.2	155.5
291	S1281	Aniracetam	AMPA Receptor-kainate Receptor-NMDA Receptor	100.6	107.2	105.6	79.8
292	S1679	Flurbiprofen	Immunology & Inflammation related	93.9	95.5	107.0	74.4
293	S1293	Cilnidipine	Calcium Channel	14.5	22.5	101.9	82.7
294	S1691	Praziquantel	Anti-infection	55.7	86.2	103.8	86.2
295	S1322	Dexamethasone (DHAP)	Autophagy,IL Receptor	53.7	90.8	113.4	83.0
296	S1705	Progesterone	Estrogen/progestogen Receptor	34.1	61.7	105.0	81.6
297	S1333	Fluoxetine HCl	5-HT Receptor	21.6	24.4	72.9	71.5
298	S1718	Adefovir Dipivoxil	Reverse Transcriptase	22.6	17.7	108.7	80.8
299	S1356	Levetiracetam	Calcium Channel	65.2	75.7	100.2	81.1
300	S1732	Mitotane	Others	92.6	78.4	105.1	78.7
301	S1369	Bafetinib (INNO-406)	Bcr-Abl	28.6	21.1	86.7	68.3
302	S1740	Guaifenesin	Others	101.3	93.1	106.3	85.6
303	S1382	Mianserin HCl	Histamine Receptor	56.3	60.1	82.4	76.7
304	S1756	Enoxacin	Topoisomerase	103.6	96.3	104.3	74.6
305	S1396	Resveratrol	Autophagy	60.2	41.5	86.8	73.2
306	S1770	Sulfadiazine	Anti-infection	91.1	100.4	109.6	80.7
307	S1408	Linezolid	Anti-infection	58.3	91.9	60.1	43.2
308	S1782	Azacitidine	DNA Methyltransferase	20.0	12.0	99.8	58.2
309	S1011	Afatinib (BIBW2992)	EGFR,HER2	19.1	13.3	56.9	44.0
310	S1437	Tizanidine HCl	Adrenergic Receptor	99.0	96.1	116.1	77.1
311	S1029	Lenalidomide (CC-5013)	TNF-alpha	89.1	95.3	110.6	79.0
312	S1452	Ispinesib (SB-715992)	Kinesin	11.9	8.8	33.2	20.5
313	S1048	Tozasertib (VX-680, MK-0457)	Aurora Kinase	21.6	16.5	81.3	77.3
314	S1472	Safinamide Mesylate	MAO	90.9	102.2	111.9	82.3
315	S1085	Belinostat (PXD101)	HDAC	24.1	19.9	97.0	83.5
316	S1491	Fludarabine	DNA/RNA Synthesis,STAT	12.3	10.9	35.1	22.0
317	S1117	Triciribine	Akt	33.0	37.4	105.9	77.1
318	S1512	Tadalafil	PDE	47.8	68.6	101.8	85.4
319	S1144	Ivacaftor (VX-770)	CFTR	103.8	100.2	94.0	71.7

320	S1548	Dapagliflozin	CFTR	55.7	57.7	103.2	79.0
321	S1163	Onalespib (AT13387)	SGLT	22.0	17.0	91.4	95.9
322	S1576	Sulfasalazine	Immunology & Inflammation related	96.6	103.3	101.4	90.7
323	S1193	Thalidomide	E3 Ligase, TNF-alpha	106.8	109.0	63.6	82.6
324	S1606	Clotrimazole	Anti-infection	72.9	56.4	112.4	63.2
325	S1202	Dutasteride	5-alpha Reductase	102.2	95.3	89.9	97.8
326	S1619	Prilocaine	Sodium Channel	107.7	103.3	104.6	84.3
327	S1213	Nelarabine	DNA/RNA Synthesis	94.6	105.4	100.2	83.7
328	S1629	Orlistat	Lipase,Fatty Acid Synthase	20.7	40.7	115.7	79.5
329	S1229	Fludarabine Phosphate	DNA/RNA Synthesis	17.2	12.9	36.9	27.4
330	S1640	Albendazole	Microtubule Associated	18.5	12.8	102.6	67.0
331	S1247	Leflunomide	Dehydrogenase	25.6	20.1	139.5	94.5
332	S1651	Telbivudine	Reverse Transcriptase	102.1	94.0	121.6	80.7
333	S1261	Celecoxib	COX	114.2	87.7	97.0	58.1
334	S1665	Estrone	Estrogen/progestogen Receptor	57.6	37.2	113.8	121.1
335	S1282	Artemisinin	Anti-infection	31.7	22.0	111.0	99.5
336	S1680	Disulfiram	Dehydrogenase	67.1	60.2	55.1	48.6
337	S1294	Cilostazol	PDE	88.0	109.4	127.5	95.1
338	S1692	Busulfan	DNA alkylator	43.9	78.1	97.2	82.9
339	S1324	Doxazosin Mesylate	Adrenergic Receptor	43.6	53.8	107.4	77.1
340	S1706	Lamivudine	Reverse Transcriptase	84.2	92.4	116.1	83.7
341	S1334	Flupirtine maleate	DNA/RNA Synthesis	82.4	79.4	111.6	79.9
342	S1719	Zalcitabine	Reverse Transcriptase	105.2	82.8	119.4	93.3
343	S1357	Lidocaine	Histamine Receptor	73.3	77.6	103.6	90.6
344	S1733	Methylprednisolone	Glucocorticoid Receptor,Immunology & Inflammation related	51.5	79.9	109.9	99.2
345	S1373	Daptomycin	Anti-infection,DNA/RNA Synthesis	91.4	98.7	44.7	38.7
346	S1741	Rifabutin	Anti-infection	107.3	108.8	127.4	76.6
347	S1385	Mosapride Citrate	5-HT Receptor	108.4	106.8	96.6	100.4
348	S1759	Pitavastatin Calcium	HMG-CoA Reductase	32.4	25.6	107.7	111.7
349	S1397	Rocuronium Bromide	AChR	113.9	116.4	106.9	98.5
350	S1771	Chlorprothixene	Dopamine Receptor	57.1	106.3	83.8	78.0

351	S1409	Alfuzosin HCl	Adrenergic Receptor	111.0	106.5	104.1	97.7
352	S1784	Vidarabine	DNA/RNA Synthesis	70.8	69.5	88.6	95.3
353	S1786	Verteporfin	VDA,Hippo pathway	18.4	13.2	60.4	39.9
354	S2126	Naftopidil	Adrenergic Receptor	87.5	82.6	103.1	82.9
355	S1801	Ranitidine Hydrochloride	Histamine Receptor	99.2	93.3	93.7	84.5
356	S2167	Bazedoxifene Acetate	Estrogen/progestogen Receptor	14.0	48.2	53.5	35.4
357	S1823	Fenoprofen Calcium	Immunology & Inflammation related	80.0	84.6	101.6	93.8
358	S2216	Mubritinib (TAK 165)	HER2	71.9	82.2	90.9	77.1
359	S1833	Butoconazole nitrate	Anti-infection	12.5	9.1	79.0	85.1
360	S2248	Silmitasertib (CX-4945)	Casein Kinase	48.2	39.7	79.9	67.8
361	S1848	Curcumin	NF-κB,HDAC,Histone Acetyltransferase,Nrf2	90.2	48.0	87.2	89.0
362	S2328	Nalidixic acid	Topoisomerase	88.9	96.7	94.5	90.6
363	S1876	Valaciclovir HCl	Anti-infection	104.8	97.3	92.1	84.7
364	S2376	Ammonium Glycyrrhizinate	Dehydrogenase	101.8	100.9	95.8	88.6
365	S1890	Nizatidine	Histamine Receptor	106.7	101.3	98.5	92.1
366	S2451	Amantadine HCl	Dopamine Receptor	106.7	97.5	87.9	80.7
367	S1902	Vitamin B12	Vitamin	95.7	100.2	89.7	92.2
368	S2459	Clozapine	5-HT Receptor	82.1	93.7	83.9	76.9
369	S1913	Tropicamide	AChR	87.1	109.9	91.4	98.4
370	S2475	Imatinib (STI571)	PDGFR	31.4	60.0	77.6	71.2
371	S1933	Triamcinolone	Glucocorticoid Receptor	63.0	93.5	104.2	102.4
372	S2489	Nateglinide	Potassium Channel	67.8	97.5	88.3	93.1
373	S1956	Miconazole Nitrate	Anti-infection	61.4	92.3	62.2	68.8
374	S2500	Propafenone HCl	Sodium Channel	21.2	14.5	85.5	78.5
375	S1969	Nefiracetam	GABA Receptor	89.7	88.3	90.7	85.1
376	S2509	Sotalol HCl	Adrenergic Receptor	101.3	101.7	85.7	85.9
377	S1987	Mometasone furoate	Glucocorticoid Receptor	57.5	89.0	83.4	70.7
378	S2521	Epinephrine bitartrate	Adrenergic Receptor	63.0	48.3	89.4	82.2
379	S2006	Pyrimethamine	DHFR	50.7	45.4	90.0	87.1
380	S2535	Econazole nitrate	Anti-infection,Calcium Channel	33.0	48.2	61.9	46.9
381	S2025	Urapidil HCl	5-HT Receptor	72.5	97.0	102.5	87.1

382	S2550	Tolterodine tartrate	AChR	54.6	69.8	82.8	81.7
383	S2040	Nimesulide	COX	83.6	91.5	97.4	88.5
384	S2561	Clomifene citrate	Estrogen/progestogen Receptor	11.8	8.7	62.6	41.4
385	S2052	Oxytetracycline Dihydrate	Anti-infection	73.9	95.7	75.3	54.9
386	S2574	Tetracycline HCl	Anti-infection	51.5	74.4	76.2	76.0
387	S2062	Tiopronin	ROS	108.4	105.3	93.6	86.2
388	S2589	Miglitol	Others	114.0	99.2	86.4	87.8
389	S2077	Atorvastatin Calcium	HMG-CoA Reductase	43.7	55.3	87.2	85.6
390	S2605	Idebenone	ROS	86.7	80.0	127.0	114.6
391	S2087	Rivastigmine Tartrate	AChR	83.7	81.8	103.6	88.6
392	S2625	Fostamatinib (R788)	Syk	24.1	20.8	76.0	81.8
393	S2101	Gabexate Mesylate	Serine Protease	94.6	97.2	65.6	71.0
394	S2727	Dacomitinib (PF299804, PF299)	EGFR	14.5	10.8	33.4	28.1
395	S2113	Cisatracurium Besylate	Adrenergic Receptor	21.0	13.7	89.8	74.6
396	S2768	Dinaciclib (SCH727965)	CDK	21.7	15.0	91.5	82.8
397	S1787	Teniposide	Topoisomerase	13.4	9.1	94.0	97.9
398	S2127	S- (+)-Rolipram	PDE	100.2	91.0	92.9	79.1
399	S1802	AICAR (Acadesine)	AMPK	99.9	100.5	65.8	86.6
400	S2169	Rosuvastatin Calcium	HMG-CoA Reductase	98.9	94.6	92.1	88.7
401	S1825	Erdosteine	Others	96.1	90.1	90.1	84.4
402	S2217	Irinotecan HCl Trihydrate	Topoisomerase	29.3	16.4	75.4	64.1
403	S1835	Azithromycin	Anti-infection,Autophagy	67.0	86.4	82.7	77.8
404	S2264	Artemether	Anti-infection	42.2	43.6	86.2	83.0
405	S1854	Bifonazole	Anti-infection	58.4	59.9	85.0	73.2
406	S2336	Orotic acid (6-Carboxyuracil)	Others	86.1	101.4	90.5	87.5
407	S1878	Ganciclovir	Anti-infection	107.7	96.4	91.7	91.6
408	S2378	Butylscopolamine Bromide	Others	86.8	86.1	85.7	86.0
409	S1891	Carbidopa	Decarboxylase	25.9	58.1	109.8	92.7
410	S2452	Amfebutamone (Bupropion) HCl	AChR,Dopamine Receptor	112.9	98.8	83.9	83.9
411	S1903	Diclofenac Sodium	COX	108.3	99.6	92.7	84.6
412	S2460	Pramipexole	Dopamine Receptor	112.9	103.2	86.9	81.8

413	S1914	Pregnenolone	Estrogen/progestogen Receptor	97.8	97.8	85.8	90.5
414	S2479	Lincomycin HCl	Anti-infection	59.7	70.7	88.0	88.5
415	S1934	Nystatin (Fungicidin)	Anti-infection	88.9	96.7	80.2	71.8
416	S2491	Nitrendipine	Calcium Channel,Autophagy	89.9	97.8	87.8	79.7
417	S1957	Sulfamethizole	Anti-infection	24.3	16.6	86.2	87.5
418	S2501	Pyrantel Pamoate	Anti-infection	22.3	15.5	79.5	80.2
419	S1971	Nicorandil	Potassium Channel	75.8	81.3	88.3	92.0
420	S2510	Spectinomycin 2HCl	Anti-infection	100.7	92.8	46.4	42.2
421	S1988	Propylthiouracil	Others	89.0	91.7	103.4	85.6
422	S2522	L-Adrenaline	Adrenergic Receptor	24.2	58.4	87.6	84.6
423	S2007	Sulindac	COX	96.6	88.8	87.1	83.9
424	S2536	Miconazole	Anti-infection	74.8	75.8	60.1	66.8
425	S2029	Uridine	DNA/RNA Synthesis	70.4	87.4	94.7	87.9
426	S2551	Sulbactam sodium	Anti-infection	72.2	80.5	92.4	84.1
427	S2041	Dyclonine HCl	Sodium Channel	14.7	10.3	77.8	85.5
428	S2564	Cloxacillin Sodium	Anti-infection	88.2	100.3	90.6	82.7
429	S2054	Orphenadrine Citrate	AChR	95.6	99.3	84.0	79.9
430	S2576	Xylometazoline HCl	Adrenergic Receptor	76.3	82.8	82.4	83.3
431	S2064	Balofloxacin	Topoisomerase	89.4	98.6	65.0	54.1
432	S2593	Tolvaptan	Vasopressin Receptor	114.1	101.8	90.8	82.0
433	S2078	Famotidine	Histamine Receptor	85.5	81.6	90.0	91.4
434	S2606	Mifepristone	Estrogen/progestogen Receptor	103.7	67.0	71.6	74.4
435	S2090	Dexmedetomidine HCl	Adrenergic Receptor	45.3	35.8	100.3	73.0
436	S2658	Omipalisib (GSK2126458, GSK458)	mTOR, PI3K	19.0	16.0	81.0	77.7
437	S2102	Rasagiline Mesylate	MAO	88.7	99.4	98.6	80.4
438	S2730	Crenolanib (CP-868596)	PDGFR	16.3	11.9	54.7	63.7
439	S2114	Dronedarone HCl	Potassium Channel,Sodium Channel,Calcium Channel	11.8	9.1	26.5	18.8
440	S2787	Laquinimod	Immunology & Inflammation related	96.3	81.0	92.4	87.3
441	S1789	Tetrabenazine (Xenazine)	Dopamine Receptor	101.3	89.9	93.3	82.0
442	S2128	Bazedoxifene HCl	Estrogen/progestogen Receptor	104.5	89.7	28.9	18.8
443	S1806	Acipimox	Others	100.4	100.9	97.0	85.0

444	S2173	Telotristat Etiprate (LX 1606 Hippurate)	Hydroxylase	39.1	45.0	54.8	76.7
445	S1827	Betaxolol HCl	Adrenergic Receptor	82.1	80.1	90.2	83.9
446	S2226	Idelalisib (CAL-101, GS-1101)	PI3K	71.2	68.7	85.9	77.3
447	S1836	Albendazole Oxide	Microtubule Associated	33.6	20.2	91.4	84.8
448	S2265	Artesunate	STAT	30.4	18.3	83.6	79.3
449	S1855	Pefloxacin Mesylate	Anti-infection	13.0	9.0	83.2	77.6
450	S2344	Piperine	P450 (e.g. CYP17)	31.2	22.3	83.4	71.7
451	S1880	Roxatidine Acetate HCl	Histamine Receptor	109.3	105.6	85.5	86.1
452	S2381	D-Mannitol	Others	87.8	94.8	81.4	79.7
453	S1894	Valsartan	RAAS	114.9	107.3	94.0	80.1
454	S2453	Benserazide HCl	Decarboxylase	37.7	47.3	96.4	87.3
455	S1904	Avobenzone	Others	65.8	68.9	90.3	83.6
456	S2461	Domperidone	Dopamine Receptor	94.6	66.7	70.8	68.5
457	S1915	Sulfamethoxazole	Anti-infection	42.3	34.2	89.4	76.0
458	S2480	Loperamide HCl	Opioid Receptor,Autophagy	16.4	17.5	70.6	68.1
459	S1937	Isoniazid	Fatty Acid Synthase	72.9	68.1	86.2	82.3
460	S2492	Novobiocin Sodium	Topoisomerase	77.0	93.6	56.9	44.0
461	S1958	Sulbactam	Anti-infection	109.9	112.0	98.8	85.1
462	S2502	Quinine HCl Dihydrate	Potassium Channel	70.9	94.8	79.4	80.6
463	S1972	Tamoxifen Citrate	Estrogen/progestogen Receptor,Autophagy	79.4	76.4	34.2	24.6
464	S2511	Sulfadoxine	Anti-infection	102.6	99.5	89.0	87.2
465	S1990	Capsaicin(Vanilloid)	Others	71.7	91.2	85.8	82.5
466	S2524	Phenytoin Sodium	Sodium Channel	80.0	86.3	86.2	85.4
467	S2011	Pramipexole 2HCl Monohydrate	Dopamine Receptor	91.6	84.1	88.9	83.4
468	S2537	Secnidazole	Anti-infection	112.3	94.9	91.4	80.2
469	S2030	Flunarizine 2HCl	Calcium Channel	17.3	12.9	97.2	81.7
470	S2552	Azelastine HCl	Histamine Receptor	20.3	16.0	66.5	69.3
471	S2042	Cyproterone Acetate	Androgen Receptor	24.4	16.8	93.2	80.6
472	S2565	Amoxicillin Sodium	Anti-infection	99.1	109.9	89.5	89.1
473	S2055	Gimeracil	Dehydrogenase	110.7	91.5	95.5	83.8
474	S2577	Phenacetin	COX	110.3	100.5	89.8	83.1

475	S2065	Lafutidine	Histamine Receptor	103.7	112.8	97.7	80.1
476	S2594	Pramiracetam	Others	115.0	90.3	89.4	78.7
477	S2079	Moexipril HCl	RAAS	98.6	88.6	94.2	75.9
478	S2607	Buflomedil HCl	Adrenergic Receptor	64.0	69.2	88.6	75.7
479	S2091	Betaxolol	Adrenergic Receptor	75.7	70.2	86.0	72.6
480	S2664	Clinofibrate	HMG-CoA Reductase	104.6	112.9	90.2	80.3
481	S2103	Naltrexone HCl	Opioid Receptor	84.6	91.9	87.8	77.0
482	S2736	Fedratinib (SAR302503, TG101348)	JAK	11.4	8.0	44.3	37.2
483	S2116	Conivaptan HCl	Vasopressin Receptor	105.7	113.5	84.2	75.0
484	S2790	Istradefylline	Adenosine Receptor	109.2	103.5	78.2	65.0
485	S1790	Rifaximin	DNA/RNA Synthesis	101.3	87.2	86.2	88.9
486	S2130	Atropine sulfate monohydrate	AChR	106.0	91.5	91.1	82.3
487	S1807	Aciclovir	Anti-infection	80.4	87.2	86.9	83.2
488	S2179	Gandotinib (LY2784544)	JAK	39.0	60.0	86.5	76.2
489	S1828	Proparacaine HCl	Sodium Channel	99.5	93.3	88.6	87.9
490	S2229	Eltrombopag Olamine	Thrombin	37.7	31.4	83.7	96.6
491	S1839	Chloroxine	Anti-infection	61.7	97.2	50.2	77.6
492	S2284	Colchicine	Microtubule Associated	22.7	15.5	80.3	80.5
493	S1856	Metoprolol Tartrate	Adrenergic Receptor	99.1	96.0	87.8	84.1
494	S2350	Rutin	Immunology & Inflammation related	103.1	95.0	92.7	82.7
495	S1881	Protionamide	Anti-infection	105.9	93.7	93.1	85.9
496	S2388	L-carnitine	Others	114.9	113.8	87.9	94.1
497	S1895	Dipyridamole	PDE	69.5	103.0	69.5	60.9
498	S2454	Bupivacaine HCl	Sodium Channel	102.4	90.0	89.4	80.7
499	S1905	Amlodipine	Calcium Channel	30.8	45.2	57.6	68.6
500	S2466	Estriol	Estrogen/progestogen Receptor	92.2	78.2	91.9	76.5
501	S1916	Sulfisoxazole	Anti-infection	77.6	70.8	95.5	82.7
502	S2481	Manidipine	Calcium Channel	66.0	109.9	64.2	60.9
503	S1940	Levofloxacin	Topoisomerase	106.9	103.8	54.6	48.1
504	S2493	Olanzapine	5-HT Receptor,Dopamine Receptor	94.3	84.5	90.5	77.3
505	S1959	Tolfenamic Acid	COX	100.2	105.9	86.2	70.4

506	S2503	Racecadotril	Opioid Receptor	112.0	112.6	83.4	78.0
507	S1974	Meglumine	Others	104.8	84.2	95.9	84.2
508	S2512	Tenoxicam	Immunology & Inflammation related	99.4	83.5	85.2	84.1
509	S1992	Fluticasone propionate	Glucocorticoid Receptor	46.1	50.5	80.1	70.4
510	S2525	Phenytoin	Sodium Channel	94.2	72.1	93.1	84.2
511	S2016	Mirtazapine	5-HT Receptor	59.7	62.6	89.8	87.6
512	S2541	Clomipramine HCl	5-HT Receptor	56.1	68.1	54.0	64.0
513	S2031	Fenticonazole Nitrate	Anti-infection	79.5	103.7	33.1	24.5
514	S2553	5-Aminolevulinic acid HCl	Others	47.8	59.5	89.9	81.3
515	S2043	Memantine HCl	P450 (e.g. CYP17),AMPA Receptor-kainate Receptor-NMDA Receptor	101.1	87.3	84.6	81.3
516	S2566	Isoprenaline HCl	Adrenergic Receptor	61.8	49.6	113.3	95.3
517	S2057	Cyclophosphamide Monohydrate	DNA alkylator	111.9	95.7	97.5	82.2
518	S2579	Zidovudine	Reverse Transcriptase	79.3	43.5	79.0	72.5
519	S2066	Moxonidine	Others	108.6	106.3	92.5	85.6
520	S2596	Clindamycin palmitate HCl	Others	113.3	96.0	78.2	76.1
521	S2080	Clevidipine Butyrate	Calcium Channel	90.0	71.2	87.4	84.1
522	S2608	Fluocinonide	Glucocorticoid Receptor	72.1	87.7	76.2	66.4
523	S2092	Detomidine HCl	Adrenergic Receptor	103.4	101.1	90.3	74.3
524	S2665	Ciprofibrate	PPAR	85.7	102.8	91.5	83.6
525	S2104	Levosulpiride	Dopamine Receptor	104.2	108.5	94.1	81.4
526	S2741	Niraparib (MK-4827)	PARP	29.3	21.4	75.4	69.0
527	S2118	Ibutilide Fumarate	Sodium Channel	86.1	101.5	81.5	75.2
528	S2791	Sotrastaurin	PKC	96.3	94.3	80.4	89.6
529	S1792	Simvastatin	HMG-CoA Reductase	111.2	79.1	53.6	58.3
530	S2131	Roflumilast	PDE	104.1	83.1	86.8	75.3
531	S1808	Nifedipine	Calcium Channel	47.9	61.2	85.2	84.5
532	S2180	Ixazomib (MLN2238)	Proteasome	11.7	10.3	79.0	75.4
533	S1829	Pranlukast	Immunology & Inflammation related	59.8	79.9	94.8	87.0
534	S2230	Galunisertib (LY2157299)	TGF-beta/Smad	89.7	71.9	88.5	80.9

535	S1840	Lomustine	DNA/RNA Synthesis	97.2	98.8	90.9	84.8
536	S2286	Cyclosporin A	Immunology & Inflammation related	15.9	10.6	38.8	35.0
537	S1859	Diethylstilbestrol	Estrogen/progestogen Receptor	74.0	69.5	88.8	79.5
538	S2357	Silibinin	Others	109.1	104.5	83.8	73.1
539	S1883	Idoxuridine	Anti-infection	33.8	21.4	87.3	89.6
540	S2393	Sorbitol	Others	117.4	88.7	90.9	79.6
541	S1896	Hydroxyurea	DNA/RNA Synthesis	104.2	81.5	97.1	77.9
542	S2455	Bethanechol chloride	AChR	119.3	88.1	90.6	82.5
543	S1907	Metronidazole	DNA/RNA Synthesis	71.4	69.4	86.6	74.4
544	S2467	Famciclovir	Anti-infection	104.0	87.6	82.2	75.2
545	S1917	Crystal Violet	Others	47.1	33.4	189.1	131.0
546	S2482	Manidipine 2HCl	Calcium Channel	14.4	10.1	43.7	44.0
547	S1941	Enalapril Maleate	RAAS	112.5	100.7	88.7	86.5
548	S2494	Olopatadine HCl	Histamine Receptor	112.0	98.1	90.0	82.9
549	S1960	Pranoprofen	COX	104.4	107.2	92.6	76.5
550	S2504	Ribavirin	Anti-infection	76.5	66.7	57.3	69.9
551	S1975	Aripiprazole	5-HT Receptor	116.1	91.5	93.5	90.9
552	S2515	Vardenafil HCl Trihydrate	PDE	100.1	85.8	77.5	77.8
553	S1994	Lacidipine	Calcium Channel	67.8	63.7	81.9	76.3
554	S2528	Ciclopirox	ATPase,Anti-infection	22.1	10.0	72.1	88.0
555	S2017	Benidipine HCl	Calcium Channel	12.5	10.1	81.7	88.1
556	S2542	Phenformin HCl	AMPK	114.9	96.2	88.5	76.9
557	S2032	Rebamipide	Others	101.8	102.2	84.3	84.1
558	S2555	Clarithromycin	P450 (e.g. CYP17)	107.0	100.0	82.6	81.3
559	S2044	Cyproheptadine HCl	Histamine Receptor	35.4	24.6	76.1	71.8
560	S2567	Medroxyprogesterone acetate	Estrogen/progestogen Receptor	86.9	102.6	110.2	84.0
561	S2058	Tolnaftate	Anti-infection	66.1	53.1	91.3	80.5
562	S2581	Quinapril HCl	RAAS	104.2	105.9	89.5	80.2
563	S2067	Ozagrel HCl	P450 (e.g. CYP17)	116.0	103.5	92.2	81.1
564	S2599	L-Thyroxine	Others	97.1	84.7	86.9	74.0
565	S2082	Adiphenine HCl	AChR	70.5	70.5	93.2	71.7
566	S2610	Lonidamine	Others	71.4	97.4	78.7	75.0
567	S2096	Almotriptan Malate	5-HT Receptor	104.0	101.7	96.9	83.8

568	S2667	Dolutegravir (GSK1349572)	Integrase	72.3	111.7	82.4	74.8
569	S2105	Pantoprazole	Proton Pump	78.0	69.2	85.5	79.9
570	S2753	Tivantinib (ARQ 197)	c-Met	23.1	15.2	75.4	77.7
571	S2119	Probucol	LDL	98.7	102.1	92.2	79.8
572	S2792	Torcetrapib	CETP	54.7	61.2	90.5	77.4
573	S1793	Ramipril	RAAS	96.2	86.6	94.2	86.9
574	S2151	Sonidegib (Erismodegib, NVP-LDE225)	Hedgehog/Smoothened	104.4	95.3	93.5	80.4
575	S1811	Amiloride HCl	Sodium Channel,Calcium Channel	101.2	91.7	74.8	82.5
576	S2181	Ixazomib Citrate (MLN9708)	Proteasome	12.0	10.7	60.5	40.2
577	S1830	Oxfendazole	Anti-infection	29.8	67.6	88.8	78.0
578	S2233	Esomeprazole sodium	ATPase	112.9	96.7	84.3	83.2
579	S1843	Chenodeoxycholic Acid	FXR	99.7	96.2	89.7	81.9
580	S2290	Dihydroartemisinin (DHA)	Others	30.6	29.6	88.4	93.7
581	S1865	Diltiazem HCl	Calcium Channel	90.7	86.2	87.6	82.2
582	S2369	Troxerutin	Others	99.9	98.6	97.8	83.8
583	S1884	Sparfloxacin	Anti-infection	104.6	99.1	48.8	48.8
584	S2422	Ipriflavone (Osteofix)	Others	83.1	87.2	84.8	81.2
585	S1897	Potassium Iodide	Others	112.5	107.3	93.7	98.3
586	S2456	Chlorpromazine HCl	Potassium Channel,Dopamine Receptor	75.8	64.4	61.5	59.7
587	S1908	Flutamide	Androgen Receptor	58.6	73.8	78.9	78.9
588	S2470	Fluocinolone Acetonide	Glucocorticoid Receptor	66.7	88.4	101.8	80.4
589	S1920	Haloperidol	Dopamine Receptor	95.4	95.3	65.3	68.7
590	S2485	Mitoxantrone 2HCl	Topoisomerase	40.9	27.0	56.4	40.9
591	S1949	Menadione	DNA/RNA Synthesis,phosphatase	13.7	9.0	95.0	86.7
592	S2496	Ozagrel	P450 (e.g. CYP17)	100.8	92.3	90.0	80.4
593	S1962	Sulphadimethoxine	Anti-infection	115.1	113.9	86.3	80.5
594	S2505	Rosiglitazone maleate	PPAR	99.4	109.2	89.9	84.9
595	S1978	Methscopolamine	AChR	101.3	81.8	94.4	90.7
596	S2516	Xylazine HCl	Adrenergic Receptor	98.6	85.2	80.8	75.2

597	S2001	Elvitegravir (GS-9137, JTK-303)	Integrase	20.0	15.0	73.1	66.5
598	S2529	Dopamine HCl	Dopamine Receptor	50.1	66.6	102.8	103.9
599	S2020	Formoterol Hemifumarate	Adrenergic Receptor	79.9	69.2	94.1	82.3
600	S2543	Ceftiofur HCl	Anti-infection	106.8	88.7	84.4	73.5
601	S2036	Aspartame	Others	108.9	97.3	94.1	86.1
602	S2556	Rosiglitazone	PPAR	103.2	93.0	83.4	81.0
603	S2045	Doxifluridine	Phosphorylase	107.7	92.4	79.5	81.2
604	S2569	Phenylephrine HCl	Adrenergic Receptor	100.7	95.2	88.5	82.4
605	S2059	Terazosin HCl Dihydrate	Adrenergic Receptor	90.4	95.9	85.1	82.9
606	S2583	Thiamphenicol	Anti-infection	97.6	92.8	58.4	61.5
607	S2069	Argatroban	Thrombin	110.0	104.6	92.7	78.9
608	S2601	Gliclazide	Potassium Channel	108.6	80.7	93.3	77.6
609	S2084	Duloxetine HCl	5-HT Receptor	54.4	65.9	45.7	47.4
610	S2613	Clorsulon	Anti-infection	69.6	82.2	88.6	76.5
611	S2097	Ambrisentan	Endothelin Receptor	99.1	89.3	86.7	79.1
612	S2673	Trametinib (GSK1120212)	MEK	88.7	92.0	92.5	97.4
613	S2108	Flunixin Meglumin	COX	36.7	27.8	91.0	84.2
614	S2755	Varlitinib	EGFR	98.5	96.4	77.7	76.2
615	S2123	Dextrose	Others	119.9	107.3	87.5	83.7
616	S2794	Sofosbuvir (PSI-7977, GS-7977)	DNA/RNA Synthesis	92.8	96.0	92.1	85.1
617	S1794	Fenofibrate	PPAR	97.7	85.8	91.7	98.8
618	S2154	Dabigatran Etexilate	Thrombin	100.3	88.7	92.1	78.9
619	S1813	Amlodipine Besylate	Calcium Channel	24.6	19.4	62.4	76.8
620	S2199	Aliskiren Hemifumarate	RAAS	103.7	69.2	94.2	70.3
621	S1831	Carvedilol	Adrenergic Receptor	26.9	30.1	69.1	64.4
622	S2235	Volasertib (BI 6727)	PLK	20.7	15.3	59.4	59.1
623	S1845	Cimetidine	Histamine Receptor	111.5	95.4	89.8	86.3
624	S2293	DL-Carnitine HCl	Others	112.1	87.5	86.1	83.6
625	S1866	Diphenhydramine HCl	Histamine Receptor	98.4	92.0	79.6	82.8
626	S2373	Yohimbine HCl	Adrenergic Receptor	81.4	94.8	83.1	75.6
627	S1885	Felodipine	Calcium Channel	66.5	58.9	86.2	77.4
628	S2442	Inosine	Others	86.3	69.0	93.4	86.4
629	S1898	Tropisetron HCl	5-HT Receptor	81.8	99.6	89.2	78.7

630	S2457	Clindamycin HCl	Anti-infection	85.0	82.3	90.1	83.0
631	S1909	Fluvastatin Sodium	HMG-CoA Reductase	62.0	62.1	87.5	88.3
632	S2471	Gallamine Triethiodide	AChR	69.6	80.3	87.5	86.6
633	S1921	Phenindione	Vitamin	75.1	82.5	98.8	90.6
634	S2486	Moroxydine HCl	Anti-infection	98.4	92.8	84.0	82.9
635	S1950	Metformin HCl	Autophagy	101.3	103.4	92.2	80.5
636	S2497	Pancuronium dibromide	AChR	106.1	105.7	87.6	82.7
637	S1964	Rimantadine	Anti-infection	90.3	101.9	81.8	79.5
638	S2506	Roxithromycin	Anti-infection	101.2	101.0	96.6	83.1
639	S1979	Amiodarone HCl	Autophagy,Potassium Channel	89.8	88.2	77.6	102.2
640	S2517	Maprotiline HCl	Adrenergic Receptor	87.0	79.9	74.4	77.0
641	S2003	Maraviroc	CCR	93.4	96.0	88.4	80.7
642	S2533	Ritodrine HCl	Adrenergic Receptor	101.8	92.7	92.7	91.4
643	S2021	Chlormezanone	Others	94.2	83.0	89.0	87.4
644	S2547	Tiotropium Bromide hydrate	AChR	77.0	48.8	87.7	86.2
645	S2037	Candesartan Cilexetil	RAAS	93.3	97.3	55.1	53.0
646	S2557	Terbinafine HCl	Anti-infection	76.3	78.3	90.8	82.8
647	S2046	Pioglitazone HCl	P450 (e.g. CYP17)	102.2	87.3	89.4	86.8
648	S2570	Prednisolone Acetate	Glucocorticoid Receptor	68.5	96.2	104.2	85.5
649	S2060	Bromhexine HCl	Others	84.8	75.5	91.0	83.2
650	S2584	Clobetasol propionate	Glucocorticoid Receptor	49.2	71.2	100.9	91.2
651	S2074	Mecarbinate	Others	57.2	82.1	76.2	69.5
652	S2602	Acemetacin	COX	61.0	73.1	92.9	79.4
653	S2085	Trimebutine	Opioid Receptor	53.3	77.6	88.1	83.5
654	S2614	Arecoline HBr	AChR	50.1	70.4	87.0	82.0
655	S2098	Bexarotene	Retinoid Receptor	58.3	81.5	85.4	80.3
656	S2680	Ibrutinib (PCI-32765)	BTK	79.6	93.0	82.4	64.0
657	S2109	Imidapril HCl	RAAS	102.3	99.3	93.0	81.7
658	S2757	Evofosfamide (TH-302)	Others	29.3	10.7	75.7	81.6
659	S2124	Xylose	Others	71.0	88.3	89.3	83.4
660	S2797	Lonafarnib	Transferase	32.7	21.7	79.2	57.2
661	S1799	Ranolazine	Calcium Channel	98.7	90.4	88.9	103.9
662	S2159	Tebipenem Pivoxil	Anti-infection	106.8	92.8	107.0	95.4
663	S1816	Chlorpheniramine Maleate	Histamine Receptor	102.2	92.8	86.8	87.1

664	S2208	Formestane	Aromatase	107.2	55.0	89.7	90.2
665	S1832	Atracurium Besylate	AChR	29.8	27.7	83.8	85.5
666	S2240	Fesoterodine Fumarate	AChR	102.9	84.7	88.2	83.7
667	S1847	Clemastine Fumarate	Histamine Receptor	43.2	74.7	61.7	63.5
668	S2296	Enoxolone	Dehydrogenase	103.7	92.7	92.5	84.2
669	S1869	Dapoxetine HCl	5-HT Receptor	47.0	48.4	70.2	78.1
670	S2374	5-hydroxytryptophan (5-HTP)	Others	89.5	94.2	88.9	84.7
671	S1888	Deflazacort	Glucocorticoid Receptor	75.5	97.3	105.6	96.8
672	S2443	Tolbutamide	Potassium Channel	77.6	83.7	94.1	90.2
673	S1899	Nicotinamide (Vitamin B3)	Sirtuin	111.2	103.6	90.2	86.9
674	S2458	Clonidine HCl	Adrenergic Receptor,Autophagy	78.1	78.1	93.0	94.9
675	S1910	Tioconazole	Anti-infection	60.0	77.6	50.9	33.7
676	S2473	Hexestrol	Estrogen/progestogen Receptor	78.6	100.7	83.4	84.4
677	S1928	Alibendol	Others	68.6	82.3	87.8	84.8
678	S2487	Mycophenolic acid	Dehydrogenase	24.8	17.1	86.3	79.1
679	S1952	Methoxsalen	P450 (e.g. CYP17)	94.2	92.5	90.0	84.5
680	S2499	Phenoxybenzamine HCl	Adrenergic Receptor	102.9	98.4	87.9	82.5
681	S1965	Primidone	Sodium Channel	101.9	99.4	90.1	85.8
682	S2508	Scopolamine HBr	AChR	104.0	108.4	98.7	87.3
683	S1983	Adenine HCl	DNA/RNA Synthesis	106.9	90.3	93.0	107.1
684	S2519	Naphazoline HCl	Adrenergic Receptor	106.3	92.1	96.3	104.6
685	S2005	Raltegravir (MK-0518)	Integrase	111.7	101.0	96.4	96.4
686	S2534	Isoconazole nitrate	Anti-infection	71.7	74.6	80.2	87.4
687	S2024	Ketotifen Fumarate	Histamine Receptor	92.7	91.2	96.8	90.4
688	S2549	Trospium chloride	AChR	114.2	93.3	91.2	90.3
689	S2038	Phentolamine Mesylate	Adrenergic Receptor	98.2	94.3	96.7	94.1
690	S2559	Cortisone acetate	Glucocorticoid Receptor	107.4	89.9	98.0	97.7
691	S2051	Captopril	RAAS	109.6	91.0	99.0	97.0
692	S2573	Tetracaine HCl	Calcium Channel	111.4	102.7	102.7	90.2
693	S2061	Lovastatin	HMG-CoA Reductase	93.7	50.4	95.3	88.2
694	S2585	Brompheniramine hydrogen maleate	HMG-CoA Reductase	96.0	98.4	91.5	86.5

695	S2075	Rosiglitazone HCl	PPAR	86.5	94.8	96.0	90.6
696	S2603	Tioxolone	Carbonic Anhydrase	113.8	88.6	105.9	92.0
697	S2086	Ivabradine HCl	Adrenergic Receptor	76.7	89.3	101.0	97.4
698	S2615	Noradrenaline bitartrate monohydrate	Adrenergic Receptor	104.2	86.6	131.5	136.3
699	S2099	Temocapril HCl	RAAS	127.8	94.0	95.1	99.3
700	S2693	Resminostat	HDAC	28.6	23.1	94.5	72.8
701	S2111	Lapatinib	EGFR, HER2	82.5	90.3	52.1	42.5
702	S2760	Canagliflozin	SGLT	49.6	47.1	98.4	72.1
703	S2125	Mestranol	Estrogen/progestogen Receptor	95.6	100.3	91.5	86.4
704	S2803	Galeterone	Androgen Receptor, P450 (e.g. CYP17)	79.9	76.2	105.6	93.8
705	S2807	Dabrafenib (GSK2118436)	Raf	62.7	61.5	110.9	90.5
706	S4025	Homatropine Bromide	AChR	105.2	97.0	110.4	105.6
707	S2907	Pirfenidone	TGF-beta/Smad	97.0	90.6	107.5	99.4
708	S4040	Norethindrone	Others	91.2	98.4	107.1	103.4
709	S3012	Pazopanib	c-Kit,PDGFR,VEGFR	107.2	104.3	87.3	95.2
710	S4048	Carbimazole	Others	110.9	105.8	112.5	100.6
711	S3027	Fenoprofen calcium hydrate	Immunology & Inflammation related	110.5	108.6	111.3	109.5
712	S4058	Ropivacaine HCl	Sodium Channel	104.5	108.7	113.3	105.6
713	S3045	Cinepazide maleate	Calcium Channel	94.4	106.1	99.9	101.3
714	S4072	Decamethonium Bromide	AChR	99.0	114.0	105.0	116.8
715	S3060	Medetomidine HCl	Adrenergic Receptor	104.2	101.1	108.1	121.6
716	S4082	Spiramycin	Anti-infection	90.5	118.3	80.3	85.4
717	S3075	Dexmedetomidine	Adrenergic Receptor	88.2	100.6	106.9	100.1
718	S4091	Ifenprodil Tartrate	NMDAR	52.8	91.4	146.0	95.8
719	S3105	Nadifloxacin	Anti-infection	79.8	108.9	203.9	43.9
720	S4102	Eprosartan Mesylate	RAAS	79.2	99.3	105.3	105.0
721	S3124	Dexamethasone Acetate	Autophagy,IL Receptor	66.7	107.3	141.8	106.0
722	S4119	Pefloxacin Mesylate Dihydrate	Topoisomerase	95.7	116.2	98.4	90.3
723	S3144	Darifenacin HBr	AChR	78.1	92.9	95.5	101.9
724	S4135	Clorprenaline HCl	Adrenergic Receptor	107.8	111.0	110.2	104.3
725	S3162	Tylosin tartrate	Anti-infection	103.4	113.3	118.2	113.5

726	S4151	Penfluridol	Dopamine Receptor	18.2	13.0	36.1	22.7
727	S3178	Brinzolamide	Carbonic Anhydrase	93.8	95.2	99.7	97.9
728	S4166	Chlorpropamide	Others	80.7	81.5	103.9	96.1
729	S3189	Ropinirole HCl	Dopamine Receptor	96.6	88.6	102.7	102.6
730	S4179	Mezlocillin Sodium	Anti-infection	64.9	60.0	102.0	101.2
731	S3207	Iopromide	Others	109.5	105.6	105.6	97.4
732	S4191	Betamipron	Anti-infection	108.5	107.1	110.1	91.1
733	S3639	Tacrine hydrochloride hydrate	AChR	107.8	110.8	98.3	94.4
734	S4203	Furaltadone HCl	Anti-infection	99.8	107.8	63.5	66.1
735	S3671	Quinestrol	Estrogen/progestogen Receptor	50.7	110.9	101.6	107.0
736	S4216	Valnemulin HCl	Anti-infection	40.6	59.0	62.4	54.6
737	S3701	Benactyzine hydrochloride	AChR	113.6	113.4	102.1	111.8
738	S4228	Fluorometholone Acetate	Glucocorticoid Receptor	63.2	104.3	110.4	104.6
739	S3724	Velpatasvir	HCV Protease	53.7	88.3	71.7	75.6
740	S4242	Cetrimonium Bromide (CTAB)	Anti-infection	11.0	9.2	28.2	19.0
741	S3733	Boceprevir	HCV Protease	94.0	111.0	91.8	78.3
742	S4252	Mechlorethamine HCl	DNA/RNA Synthesis	17.5	11.8	106.2	100.1
743	S3747	Levothyroxine sodium	Others	110.7	122.9	99.4	93.8
744	S4267	Diacerein	IL Receptor	85.7	97.2	92.0	81.7
745	S4002	Sitagliptin phosphate monohydrate	DPP-4	74.4	92.5	102.2	103.7
746	S4282	Nelfinavir Mesylate	HIV Protease	74.8	83.1	42.9	38.7
747	S4014	Hyoscyamine	AChR	104.6	121.2	94.5	113.1
748	S4291	Labetalol HCl	Adrenergic Receptor	88.3	114.3	102.0	102.0
749	S2814	Alpelisib (BYL719)	PI3K	34.5	26.4	90.3	90.8
750	S4026	Hydroxyzine 2HCl	Histamine Receptor	67.8	85.4	88.6	86.6
751	S2922	Icotinib	EGFR	85.8	76.7	94.0	87.6
752	S4041	Olsalazine Sodium	Anti-infection	67.6	65.9	104.6	89.1
753	S3015	Amoxicillin	Anti-infection	98.7	109.3	98.3	100.3
754	S4049	Valdecoxib	COX	52.1	106.5	97.1	90.8
755	S3031	Linagliptin	DPP-4	102.0	103.8	92.1	88.0
756	S4060	Erythromycin Ethylsuccinate	Anti-infection	106.2	111.0	96.6	91.4

757	S3046	Azilsartan	RAAS	104.9	111.8	103.0	88.6
758	S4073	Sodium 4-Aminosalicylate	NF-κB	112.2	121.9	98.4	92.6
759	S3061	Epinephrine HCl	Adrenergic Receptor	60.1	57.4	101.8	140.6
760	S4083	Vitamin A Acetate	Vitamin	64.9	91.0	98.0	96.5
761	S3076	Foscarnet Sodium	Reverse Transcriptase	78.6	101.7	100.1	95.6
762	S4092	Pramoxine HCl	Others	94.3	109.6	103.5	94.8
763	S3106	Pidotimod	Immunology & Inflammation related	104.9	112.0	94.7	89.6
764	S4107	Clofazimine	Phospholipase (e.g. PLA)	13.4	11.0	35.7	42.2
765	S3129	Trimethoprim	Anti-infection	103.8	108.2	103.4	92.9
766	S4120	Sulconazole Nitrate	Anti-infection	34.3	87.6	48.3	52.5
767	S3149	Estradiol valerate	Estrogen/progestogen Receptor	72.1	48.1	99.7	88.0
768	S4136	Carprofen	COX	100.4	112.3	111.3	92.7
769	S3163	Benztropine mesylate	Dopamine Receptor	80.7	113.3	96.4	87.1
770	S4152	Ethamsylate	Immunology & Inflammation related	136.6	135.0	122.2	107.7
771	S3179	Carbenicillin disodium	Anti-infection	84.3	87.1	100.6	106.7
772	S4167	Cyromazine	Anti-infection	102.7	105.9	103.9	96.6
773	S3193	Ticarcillin sodium	Anti-infection	94.0	87.5	106.8	95.4
774	S4181	Nicardipine HCl	Calcium Channel	15.7	18.4	104.3	88.1
775	S3208	Fexofenadine HCl	Histamine Receptor	96.6	107.7	106.2	92.2
776	S4192	Chlorquinaldol	Anti-infection	20.3	17.3	44.4	58.3
777	S3640	Methoxyphenamine Hydrochloride	Adrenergic Receptor	101.2	115.5	101.4	94.7
778	S4204	Isosorbide	Others	97.6	97.5	102.2	88.3
779	S3672	Cefonicid sodium	Anti-infection	116.6	116.2	100.0	94.0
780	S4217	Liothyronine Sodium	Others	82.6	108.3	64.6	73.1
781	S3706	Sarpogrelate hydrochloride	5-HT Receptor	80.6	96.9	89.8	110.1
782	S4229	Oxybuprocaine HCl	Sodium Channel	75.9	102.3	109.1	84.1
783	S3726	Selexipag	Immunology & Inflammation related	75.3	100.0	97.3	94.5
784	S4243	Deoxycorticosterone acetate	Adrenergic Receptor	85.1	105.1	94.6	91.0
785	S3735	Umeclidinium bromide	AChR	82.3	101.9	75.8	77.2
786	S4253	Epinastine HCl	Histamine Receptor	90.5	105.1	86.1	86.8

787	S3751	Quinidine sulfate	Sodium Channel	51.1	83.0	86.9	77.7
788	S4268	Flufenamic acid	Immunology & Inflammation related	21.5	37.3	96.9	93.7
789	S4003	Lithocholic acid	FXR	84.6	103.5	102.4	88.9
790	S4283	Cyclobenzaprine HCl	5-HT Receptor	14.5	11.1	93.5	88.8
791	S4016	Ouabain	Sodium Channel	15.5	13.2	102.6	70.8
792	S4292	Diphenidol HCl	AChR	101.0	118.5	101.0	85.5
793	S2823	Tideglusib	GSK-3	102.4	96.1	102.4	90.2
794	S4031	Aclidinium Bromide	AChR	97.3	98.4	89.4	91.4
795	S2925	Evacetrapib (LY2484595)	CETP	81.8	33.9	99.4	91.5
796	S4042	Nafcillin Sodium	Anti-infection	77.8	84.7	97.9	90.1
797	S3017	Aspirin	COX	110.2	113.4	100.5	87.2
798	S4050	Valganciclovir HCl	Anti-infection	105.7	107.7	105.9	87.1
799	S3032	Bindarit	Immunology & Inflammation related	102.8	104.9	106.8	89.0
800	S4061	Levobupivacaine HCl	Sodium Channel	95.8	100.9	106.2	85.5
801	S3047	Otilonium Bromide	AChR	14.2	32.5	84.3	49.4
802	S4075	Zinc Pyrithione	Proton Pump,Anti-infection	14.5	27.8	30.1	21.5
803	S3062	Diclofenac Potassium	Immunology & Inflammation related	115.9	104.2	96.9	105.2
804	S4084	Lomerizine 2HCl	Calcium Channel	15.3	11.8	84.7	116.3
805	S3077	Tazobactam	Anti-infection	82.3	109.5	102.3	98.9
806	S4095	Difluprednate	Others	67.6	91.4	107.8	84.5
807	S3113	Pyridoxine HCl	Vitamin	48.6	43.6	102.1	89.8
808	S4110	Estradiol Benzoate	Estrogen/progestogen Receptor	71.7	85.3	99.0	93.9
809	S3130	Biotin (Vitamin B7)	Vitamin	17.5	13.0	103.6	86.7
810	S4122	Tilmicosin	Anti-infection	12.2	9.4	104.9	87.1
811	S3150	Articaine HCl	Others	16.0	10.4	98.9	90.1
812	S4138	Dropropizine	Others	13.1	10.5	105.3	90.8
813	S3167	Altrenogest	Estrogen/progestogen Receptor	36.5	48.2	103.4	89.5
814	S4155	Chlorzoxazone	P450 (e.g. CYP17)	130.5	120.2	109.6	93.8
815	S3180	Eletriptan HBr	5-HT Receptor	41.4	23.2	96.7	97.5
816	S4170	Coumarin	Immunology & Inflammation related	100.4	97.0	108.8	96.6
817	S3195	Azlocillin sodium salt	Anti-infection	94.5	45.5	100.8	87.6

818	S4182	Nifuroxazide	STAT	12.9	37.0	46.0	57.8
819	S3212	Moclobemide (Ro 111163)	MAO	108.1	112.8	104.4	91.3
820	S4195	Broxyquinoline	Anti-infection	105.6	105.2	97.1	86.1
821	S3647	Mafenide Acetate	Carbonic Anhydrase	110.4	117.0	102.1	89.9
822	S4207	Clofibric Acid	PPAR	110.2	109.4	100.7	86.3
823	S3673	Sulfaphenazole	P450 (e.g. CYP17)	111.2	121.6	104.9	102.4
824	S4219	Azaperone	Dopamine Receptor	103.0	116.4	102.4	97.5
825	S3711	Carbasalate Calcium	Others	116.8	99.5	98.7	106.9
826	S4230	Oxaprozin	COX	64.0	57.9	82.2	88.7
827	S3727	Vilanterol Trifenate	Adrenergic Receptor	47.2	59.3	86.3	82.9
828	S4244	Serotonin HCl	5-HT Receptor	60.9	75.7	99.7	98.8
829	S3739	Calcipotriene	Others	41.6	28.9	102.4	95.8
830	S4256	Buspirone HCl	5-HT Receptor	31.5	22.0	101.9	86.3
831	S3945	L-Cycloserine	Transferase	33.0	22.8	106.2	85.3
832	S4269	Vinorelbine Tartrate	Microtubule Associated	12.4	9.7	83.5	82.8
833	S4004	Ethambutol 2HCl	Anti-infection	15.7	13.5	96.2	92.3
834	S4284	Chloroprocaine HCl	Sodium Channel	48.7	40.6	107.9	97.4
835	S4019	Avanafil	PDE	101.9	104.5	96.8	89.1
836	S4293	Promethazine HCl	Histamine Receptor	59.4	68.0	94.7	71.2
837	S2830	Clindamycin	Others	64.5	76.4	94.2	96.1
838	S4034	Diphemanil Methylsulfate	AChR	103.7	115.0	98.3	94.9
839	S3000	Carbazochrome sodium sulfonate (AC-17)	Others	102.5	82.1	108.2	90.8
840	S4043	Tetrahydrozoline HCl	Adrenergic Receptor	88.2	102.5	105.8	88.2
841	S3018	Niflumic acid	COX,GABA Receptor	44.4	44.7	101.5	90.9
842	S4051	Nabumetone	COX	90.1	93.2	65.5	80.4
843	S3035	Daunorubicin HCl	Topoisomerase	12.9	9.7	40.8	26.0
844	S4062	Ronidazole	Anti-infection	101.1	109.1	107.5	94.7
845	S3051	Bosentan Hydrate	Endothelin Receptor	86.3	89.9	100.1	87.3
846	S4076	Propranolol HCl	Adrenergic Receptor	96.9	115.6	100.2	80.4
847	S3063	Diclofenac Diethylamine	COX	114.5	108.3	111.3	110.1
848	S4085	Levobetaxolol HCl	Adrenergic Receptor	82.7	125.8	99.1	90.0
849	S3078	Beclomethasone dipropionate	Glucocorticoid Receptor	66.0	89.9	101.3	90.4
850	S4096	Droperidol	Dopamine Receptor	82.7	108.4	97.7	84.1

851	S3114	Vitamin C	Vitamin	45.5	37.4	103.9	99.4
852	S4111	Dicloxacillin Sodium	Anti-infection	32.6	22.7	106.7	82.8
853	S3132	Sulfamerazine	Anti-infection	78.2	101.0	102.0	84.8
854	S4123	Timolol Maleate	Adrenergic Receptor	32.3	21.7	100.0	92.1
855	S3151	Gliquidone	Potassium Channel	29.9	19.2	103.6	90.7
856	S4139	Cyclizine 2HCl	Histamine Receptor	17.5	15.3	99.5	87.7
857	S3170	Ampicillin sodium	Anti-infection	108.1	118.5	96.7	90.1
858	S4159	Bezafibrate	PPAR	110.9	118.6	96.4	89.7
859	S3181	Flumequine	Topoisomerase	25.4	17.2	107.8	92.6
860	S4171	Choline Chloride	AChR	94.8	101.8	98.1	94.6
861	S3196	Azacyclonol	5-HT Receptor	94.4	36.4	107.5	83.3
862	S4184	Penciclovir	Anti-infection	106.7	110.0	104.7	93.1
863	S3621	Pazufloxacin mesylate	Anti-infection	96.9	110.1	60.1	49.3
864	S4196	Ethacridine lactate monohydrate	Anti-infection	13.7	13.6	29.8	29.4
865	S3648	Amlexanox	Immunology & Inflammation related	57.5	43.9	96.2	88.0
866	S4208	Chromocarb	Others	106.0	106.0	102.3	88.3
867	S3684	Methacholine chloride	AChR	108.5	112.8	110.0	87.7
868	S4221	Benzbromarone	P450 (e.g. CYP17)	72.9	113.6	104.6	110.7
869	S3714	Lifitegrast	Integrin	110.3	111.3	102.7	101.2
870	S4231	Pilocarpine HCl	AChR	77.5	107.9	103.4	99.6
871	S3728	Grazoprevir	HCV Protease	57.0	60.6	86.9	95.3
872	S4246	Tranylcypromine (2-PCPA) HCl	MAO	103.1	112.1	100.9	84.9
873	S3741	Benznidazole	Anti-infection	24.8	32.4	96.1	90.6
874	S4257	Alizapride HCl	Dopamine Receptor	50.7	44.7	96.9	84.6
875	S3968	Histamine	Histamine Receptor	55.1	58.1	114.6	92.9
876	S4270	Oxiracetam	GABA Receptor	31.4	21.5	105.0	94.6
877	S4007	Pentamidine isethionate	phosphatase	12.6	9.1	48.7	65.7
878	S4285	Ospemifene	Estrogen/progestogen Receptor	16.3	13.5	102.1	91.3
879	S4020	Sodium Picosulfate	Others	88.6	108.9	99.1	88.9
880	S4294	Procainamide HCl	DNA Methyltransferase,Sodium Channel	100.1	109.3	96.7	87.3

881	S2853	Carfilzomib (PR-171)	Proteasome	26.3	18.3	89.4	93.2
882	S4035	Vitamin D2	DNA/RNA Synthesis	23.5	16.9	92.2	95.1
883	S3001	Clevudine	DNA/RNA Synthesis	85.2	21.5	101.8	92.5
884	S4044	Toltrazuril	Anti-infection	76.6	114.4	101.4	92.4
885	S3021	Rimonabant	Cannabinoid Receptor	35.5	29.7	103.4	93.4
886	S4053	Sertraline HCl	5-HT Receptor	11.6	8.8	33.9	25.1
887	S3036	Pravastatin sodium	HMG-CoA Reductase	78.2	94.8	109.4	84.8
888	S4063	Cholecalciferol (Vitamin D3)	Vitamin	24.3	16.7	104.5	84.7
889	S3052	Rupatadine Fumarate	Histamine Receptor	25.3	20.3	83.8	89.8
890	S4078	Mefenamic Acid	COX	106.3	118.1	109.9	94.0
891	S3066	Naloxone HCl	Opioid Receptor	112.7	110.1	103.2	111.7
892	S4086	Loxapine Succinate	5-HT Receptor,Dopamine Receptor	36.8	47.3	89.5	91.1
893	S3079	Atovaquone	Anti-infection	41.3	21.5	93.9	83.3
894	S4098	Halcinonide	Immunology & Inflammation related	70.4	104.1	112.5	78.4
895	S3116	Sulfathiazole	Anti-infection	50.2	45.3	99.6	85.6
896	S4112	Desvenlafaxine Succinate	Adrenergic Receptor,5-HT Receptor	59.6	58.6	91.5	92.2
897	S3133	Sulfamethazine	Anti-infection	55.1	58.8	110.2	90.7
898	S4124	Tolazoline HCl	Adrenergic Receptor	30.5	21.2	106.1	94.4
899	S3154	Butenafine HCl	Anti-infection	31.6	19.0	98.1	95.0
900	S4141	Dinitolmide	Anti-infection	13.1	9.9	108.6	91.8
901	S3172	Anagrelide HCl	PDE	77.1	91.1	96.0	96.5
902	S4160	Penicillin G Sodium	Anti-infection	57.6	64.0	100.5	85.1
903	S3183	Amitriptyline HCl	5-HT Receptor	28.8	22.2	90.7	89.5
904	S4172	Cetylpyridinium Chloride	Anti-infection	12.9	9.4	27.3	19.1
905	S3199	Reboxetine mesylate	Others	106.3	106.4	96.8	94.1
906	S4185	Tiratricol	Others	38.9	41.9	91.3	70.7
907	S3635	Medroxyprogesterone	Estrogen/progestogen Receptor	60.3	33.1	103.0	86.9
908	S4197	Bemegride	GABA Receptor	99.5	106.9	109.0	90.0
909	S3655	Cefepime Dihydrochloride Monohydrate	Anti-infection	41.1	24.7	99.6	87.6
910	S4209	Chlorocresol	Calcium Channel	99.7	98.1	105.7	91.4

911	S3690	Pargyline hydrochloride	MAO	105.4	107.9	101.2	115.8
912	S4222	Piperacillin Sodium	Anti-infection	104.9	109.1	105.1	118.3
913	S3716	Flibanserin	5-HT Receptor	109.8	106.4	97.5	111.2
914	S4233	Zoxazolamine	Others	77.5	103.5	95.2	94.2
915	S3729	Iron sucrose	Others	62.9	90.9	84.7	82.2
916	S4247	Prucalopride Succinate	5-HT Receptor	90.0	94.0	99.2	84.1
917	S3742	Cholic acid	Others	50.0	69.5	95.9	92.7
918	S4258	Luliconazole	Anti-infection	30.6	20.0	102.3	83.1
919	S3980	Pyridoxine	Vitamin	96.7	102.3	108.8	88.2
920	S4274	Rotigotine	Dopamine Receptor	33.8	40.3	103.4	93.6
921	S4009	Mirabegron	Adrenergic Receptor	29.7	20.9	96.8	96.5
922	S4286	Anidulafungin (LY303366)	Anti-infection	16.8	15.5	68.0	67.6
923	S4021	Tolcapone	Transferase	90.2	79.3	104.9	98.9
924	S4295	Meclofenamate Sodium	COX	74.4	85.7	67.5	71.9
925	S2874	Camostat Mesilate	Sodium Channel	74.4	97.7	96.0	95.8
926	S4037	Doxapram HCl	Potassium Channel	104.9	115.1	89.1	93.0
927	S3002	Rivaroxaban	Factor Xa	107.7	96.4	87.3	81.1
928	S4045	Pheniramine Maleate	Histamine Receptor	76.5	84.2	89.8	84.8
929	S3022	Cabazitaxel	Microtubule Associated	31.7	21.7	88.8	82.2
930	S4054	Spironolactone	Androgen Receptor	41.5	34.4	103.0	90.4
931	S3037	Bepotastine Besilate	Histamine Receptor	36.1	22.7	105.1	94.0
932	S4065	Guanabenz Acetate	Adrenergic Receptor	68.5	87.1	103.9	84.6
933	S3053	Azelnidipine	Calcium Channel	133.4	125.5	97.4	83.1
934	S4079	Ticagrelor	P2 Receptor	111.8	118.9	98.8	92.1
935	S3067	Chlorhexidine?2HCl	Anti-infection	11.3	8.9	27.7	21.8
936	S4088	Flumethasone	Glucocorticoid Receptor	66.9	113.8	105.7	82.3
937	S3081	Ulipristal	Estrogen/progestogen Receptor	80.3	73.9	99.2	97.1
938	S4099	Dexlansoprazole	Proton Pump	77.8	98.3	92.5	84.1
939	S3117	Oxybutynin chloride	AChR	63.9	58.5	94.2	98.5
940	S4113	Desvenlafaxine	5-HT Receptor,Adrenergic Receptor	65.4	75.1	99.9	88.6
941	S3137	Sodium salicylate	NF-κB	56.4	68.3	115.2	96.8
942	S4125	Sodium Phenylbutyrate	HDAC	72.6	87.0	107.2	87.8
943	S3155	Mepivacaine HCl	Sodium Channel	29.1	20.8	105.0	96.8

944	S4147	Azithromycin Dihydrate	Anti-infection	12.6	9.6	101.0	90.1
945	S3173	Antipyrine	COX	25.6	19.9	111.8	93.3
946	S4162	Benzethonium Chloride	AChR	11.5	9.3	28.5	19.6
947	S3185	Adrenalone HCl	Adrenergic Receptor	96.6	96.3	110.8	104.6
948	S4175	Sulfaguanidine	Anti-infection	107.1	105.3	97.0	85.8
949	S3200	Triflusal	Thrombin	107.5	103.0	110.0	96.5
950	S4186	Domiphen Bromide	Others	12.0	8.9	27.9	19.5
951	S3636	Cefadroxil hydrate	Anti-infection	122.2	110.6	117.7	97.0
952	S4198	Aminothiazole	Anti-infection	106.2	107.5	99.1	96.3
953	S3659	Fludrocortisone acetate	Others	62.4	94.1	103.4	86.9
954	S4210	Benzocaine	Sodium Channel	105.3	100.6	102.6	88.8
955	S3694	Glucosamine hydrochloride	Others	113.9	114.5	102.9	89.0
956	S4223	Mevastatin	HMG-CoA Reductase	77.0	95.7	93.5	92.1
957	S3717	Testosterone Enanthate	Androgen Receptor	86.6	65.7	99.9	127.3
958	S4235	Phenazopyridine HCl	Sodium Channel	56.1	89.3	101.2	85.3
959	S3730	Metaxalone	Others	69.1	92.9	102.7	88.1
960	S4248	Bromfenac Sodium	COX	63.1	80.6	97.2	82.7
961	S3743	Oxiglutatione	Others	50.6	72.5	102.9	93.9
962	S4260	Tamibarotene	Retinoid Receptor	53.3	63.7	102.2	82.2
963	S3997	Oxaceprol	Immunology & Inflammation related	57.1	69.6	105.9	87.6
964	S4278	Carteolol HCl	Adrenergic Receptor	52.8	57.3	100.5	93.2
965	S4010	Acebutolol HCl	Adrenergic Receptor	30.9	20.2	95.0	92.4
966	S4288	Chloroambucil	DNA/RNA Synthesis	27.7	20.5	97.7	86.0
967	S4022	Probenecid	TRPV	77.0	86.6	110.8	92.2
968	S4296	Salmeterol Xinafoate	Adrenergic Receptor	44.4	57.6	87.5	85.2
969	S2875	Prucalopride	5-HT Receptor	102.3	102.9	97.1	89.5
970	S4038	Dibucaine HCl	Sodium Channel	75.1	88.4	88.5	81.8
971	S3005	Paroxetine HCl	AChR,5-HT Receptor	83.6	79.8	77.4	77.1
972	S4046	Estradiol Cypionate	Estrogen/progestogen Receptor	20.6	65.0	87.9	73.3
973	S3023	Bufexamac	COX	49.4	83.2	103.9	89.1
974	S4056	Retapamulin	Anti-infection	62.3	81.9	61.2	62.8
975	S3038	Fosaprepitant dimeglumine salt	Others	68.5	93.7	85.0	82.4

976	S4068	Tinidazole	Anti-infection	92.9	100.0	102.8	83.3
977	S3054	Alverine Citrate	OX Receptor	88.5	96.8	94.1	95.9
978	S4080	Triamterene	Sodium Channel	91.2	92.8	101.6	103.7
979	S3070	Piracetam	GluR	73.8	88.3	97.6	98.7
980	S4089	Halobetasol Propionate	Phospholipase (e.g. PLA)	61.3	104.3	117.3	91.2
981	S3083	Indacaterol Maleate	Adrenergic Receptor	39.5	58.9	94.2	89.8
982	S4100	Esmolol HCl	Adrenergic Receptor	69.2	86.7	111.4	84.6
983	S3120	Doxepin HCl	Histamine Receptor	60.6	83.7	99.5	84.3
984	S4114	Triclabendazole	Microtubule Associated	36.3	42.9	107.4	92.0
985	S3139	Methenamine	Anti-infection	50.4	62.2	106.0	93.7
986	S4128	Troxipide	Others	37.4	39.5	101.6	93.8
987	S3160	Ethynodiol diacetate	Estrogen/progestogen Receptor	45.8	27.3	103.8	92.3
988	S4148	Ampicillin Trihydrate	Anti-infection	27.5	20.3	110.6	89.9
989	S3175	Atomoxetine HCl	5-HT Receptor	22.4	18.7	90.1	84.7
990	S4164	Doxofylline	PDE	106.3	103.9	96.7	92.9
991	S3186	Azatadine dimaleate	Histamine Receptor	100.2	99.2	105.3	96.5
992	S4176	Trometamol	COX	105.9	99.8	102.3	92.1
993	S3201	Trifluoperazine 2HCl	Dopamine Receptor	82.8	78.2	30.3	20.1
994	S4189	Cyclandelate	Others	70.0	86.9	105.0	92.2
995	S3637	Cefpirome sulfate	Anti-infection	89.2	102.4	101.2	87.6
996	S4200	Tolperisone HCl	Sodium Channel	19.8	13.7	103.2	91.0
997	S3663	Afloqualone	GABA Receptor	62.8	55.1	95.1	84.6
998	S4211	Montelukast Sodium	LTR	84.3	82.0	97.7	92.1
999	S3697	Mafenide hydrochloride	Carbonic Anhydrase	111.7	107.1	102.3	108.4
1000	S4225	Mexiletine HCl	Sodium Channel	107.7	104.5	96.5	119.3
1001	S3718	Leuprolide Acetate	Estrogen/progestogen Receptor	63.4	71.7	104.7	110.6
1002	S4237	Primaquine Diphosphate	Anti-infection	27.6	30.3	99.6	79.7
1003	S3731	Tipiracil hydrochloride	Phosphorylase	60.3	93.9	95.2	89.0
1004	S4249	Flopropione	5-HT Receptor	64.6	86.7	105.3	105.8
1005	S3745	Balsalazide disodium	Immunology & Inflammation related	53.5	77.9	96.4	91.0
1006	S4264	Etofibrate	Others	37.0	49.9	97.7	91.1
1007	S4000	Pergolide Mesylate	Dopamine Receptor	28.5	22.1	99.9	84.5
1008	S4279	Demeclocycline HCl	Anti-infection	25.8	30.8	60.7	56.0
1009	S4011	Ampiroxicam	COX	15.0	9.6	104.9	90.9

1010	S4289	Metoclopramide HCl	Dopamine Receptor	12.6	9.2	107.1	96.0
1011	S4023	Procaine HCl	NMDAR,Sodium Channel,AChR	12.6	9.3	100.0	97.5
1012	S4297	Mupirocin	DNA/RNA Synthesis	69.9	93.2	57.4	47.7
1013	S2900	Cobicistat (GS-9350)	P450 (e.g. CYP17)	102.3	99.5	76.9	79.9
1014	S4039	Methazolamide	Carbonic Anhydrase	99.1	99.4	104.1	97.1
1015	S3008	Zaltoprofen	COX	103.2	89.9	102.5	102.4
1016	S4047	Bisacodyl	Others	87.8	99.9	106.5	98.7
1017	S3024	Lamotrigine	Sodium Channel,5-HT Receptor	96.9	98.9	103.9	95.1
1018	S4057	Methyclothiazide	Others	102.6	108.1	101.9	98.1
1019	S3043	Rofecoxib	COX	102.7	108.6	104.3	97.3
1020	S4071	Griseofulvin	Microtubule Associated	34.6	73.2	99.1	91.4
1021	S3057	Azilsartan Medoxomil	RAAS	115.1	117.4	97.8	91.5
1022	S4081	Sulfacetamide Sodium	Anti-infection,Autophagy	112.2	105.5	106.2	109.9
1023	S3074	Chlorthalidone	Others	83.3	96.4	101.3	119.2
1024	S4090	Fenspiride HCl	PDE	70.0	101.1	99.7	93.3
1025	S3104	Moguisteine	Others	69.0	100.7	105.8	92.9
1026	S4101	Voglibose	Others	66.7	87.7	105.6	90.5
1027	S3121	Ornidazole	Anti-infection	51.9	79.4	101.9	99.6
1028	S4118	Histamine 2HCl	Histamine Receptor	54.4	79.0	109.5	106.5
1029	S3140	Milnacipran HCl	5-HT Receptor	32.1	29.4	113.5	95.3
1030	S4131	Levodropropizine	Histamine Receptor	40.8	51.9	105.2	105.1
1031	S3161	Sertaconazole nitrate	Anti-infection	16.8	9.8	54.6	50.4
1032	S4149	Amfenac Sodium Monohydrate	COX	37.6	48.7	111.3	93.9
1033	S3176	Betahistine 2HCl	Histamine Receptor	22.1	11.8	114.0	104.4
1034	S4165	Benzydamine HCl	Immunology & Inflammation related	27.2	15.8	86.7	73.6
1035	S3188	(+,-)-Octopamine HCl	Dopamine Receptor	104.0	101.9	106.8	110.1
1036	S4178	Climbazole	Anti-infection	91.3	96.8	93.4	94.8
1037	S3204	Meptazinol HCl	Opioid Receptor	107.2	103.8	103.9	105.2
1038	S4190	Cinchophen	Others	58.1	84.1	106.4	99.9
1039	S3638	Cefamandole nafate	Anti-infection	109.4	99.5	109.4	103.4
1040	S4201	Florfenicol	Anti-infection	104.6	102.0	66.7	64.0

1041	S3664	Flupenthixol dihydrochloride	Dopamine Receptor,Adrenergic Receptor	44.5	44.7	35.1	28.4
1042	S4213	Dirithromycin	Anti-infection	98.6	101.8	99.0	100.5
1043	S3698	Nortriptyline hydrochloride	Others	44.7	33.2	75.2	91.9
1044	S4227	Fidaxomicin	DNA/RNA Synthesis	21.6	56.0	105.7	120.7
1045	S3723	Ramosetron Hydrochloride	5-HT Receptor	105.4	106.2	93.5	107.7
1046	S4240	Doxylamine Succinate	Histamine Receptor	104.5	106.1	103.2	118.3
1047	S3732	Avibactam sodium	Anti-infection	73.5	100.4	101.7	104.9
1048	S4250	Sulfamethoxypyridazine	Anti-infection	98.5	106.8	103.9	95.2
1049	S3746	Lumefantrine	Anti-infection	75.4	99.8	99.4	100.9
1050	S4266	Brimonidine Tartrate	Adrenergic Receptor	61.4	81.6	103.3	109.2
1051	S4001	Cabozantinib malate (XL184)	TAM Receptor, VEGFR	29.6	20.0	102.9	93.9
1052	S4280	Meclofenoxate (Centrophenoxine) HCl	Transferase	37.5	30.0	120.0	108.9
1053	S4012	Desloratadine	Histamine Receptor	27.6	19.4	92.2	94.6
1054	S4290	Digoxin	Sodium Channel	26.6	18.9	107.9	79.9
1055	S4024	Homatropine Methylbromide	AChR	33.1	22.7	115.3	106.6
1056	S4299	Dicoumarol	Others	95.2	100.1	109.1	116.4
1057	S4420	Mefloquine HCl	Anti-infection	13.1	10.1	29.9	28.6
1058	S5007	Delamanid	Anti-infection	82.5	74.5	105.3	96.2
1059	S4514	Acetylleucine	Others	116.9	110.9	114.4	102.1
1060	S5027	Mebeverine Hydrochloride	Others	101.5	99.4	101.8	93.1
1061	S4527	Fenofibric acid	PPAR	113.6	104.5	106.5	96.3
1062	S5059	Pixantrone Maleate	Topoisomerase	26.4	19.5	75.8	62.2
1063	S4539	Salicylic acid	COX	113.1	98.7	115.2	105.4
1064	S5084	Carbazochrome	Others	113.3	94.9	112.1	96.8
1065	S4553	Bronopol	Anti-infection	15.6	12.3	109.3	97.6
1066	S6003	Ataluren (PTC124)	CFTR	106.3	111.2	110.6	95.3
1067	S4564	Diethylcarbamazine citrate	Anti-infection	113.0	104.6	126.7	97.1
1068	S7157	Ilomastat (GM6001, Galardin)	MMP	61.6	81.7	206.7	123.4
1069	S4580	Hydroquinone	ROS	47.9	46.3	141.4	115.1

1070	S7280	Edoxaban	Factor Xa	103.9	95.4	113.8	109.5
1071	S4594	Cephalothin	Anti-infection	100.0	87.2	109.2	105.4
1072	S7440	Ribociclib (LEE011)	CDK	36.5	30.8	108.8	91.1
1073	S4607	2,2′-Dihydroxy-4-methoxybenzophenone	Others	107.0	97.0	112.9	102.3
1074	S7625	Niraparib (MK-4827) tosylate	PARP	40.3	23.6	99.6	94.0
1075	S4623	Methylbenactyzine Bromide	AChR	92.7	74.3	114.4	118.0
1076	S7765	Dovitinib (TKI258) Lactate	FLT3,c-Kit,FGFR,PDGFR,VEGFR	58.1	41.5	110.3	111.0
1077	S4633	Isosorbide Mononitrate	Others	76.8	68.5	111.5	99.8
1078	S7799	Pexmetinib (ARRY-614)	p38 MAPK,Tie-2	40.6	25.6	94.6	66.7
1079	S4641	Tedizolid Phosphate	Anti-infection	95.2	108.9	58.0	50.3
1080	S7864	Oltipraz	Nrf2	111.3	103.3	101.4	83.8
1081	S4655	Sulpiride	Dopamine Receptor	108.2	109.2	106.8	97.1
1082	S8021	Vortioxetine (Lu AA21004) HBr	5-HT Receptor	57.7	12.9	44.3	25.6
1083	S4664	Molsidomine	Others	112.8	106.5	110.4	93.1
1084	S8103	Sotagliflozin (LX4211)	SGLT	103.5	94.0	107.8	92.0
1085	S4678	Povidone iodine	Anti-infection	104.7	93.3	107.3	88.3
1086	S8219	Bitopertin	GlyT	98.6	103.5	130.7	96.1
1087	S4691	Oxybenzone	Others	103.2	102.2	114.9	93.3
1088	S4820	Diastase	Others	110.0	111.8	116.2	97.0
1089	S4731	Perphenazine	Dopamine Receptor	12.1	22.2	114.5	55.5
1090	S3944	Valproic acid	HDAC	111.8	107.7	69.1	94.4
1091	S4815	L-Cysteine HCl	Others	118.2	95.8	115.5	103.3
1092	S4687	Rivastigmine	AChR	76.1	89.1	114.7	96.3
1093	S4835	Aceclofenac	Immunology & Inflammation related	36.4	29.8	99.1	94.6
1094	S4848	Dalbavancin	Anti-infection	105.8	107.4	101.6	111.0
1095	S4896	Elagolix Sodium	Estrogen/progestogen Receptor	70.8	65.2	101.3	88.7
1096	S4935	Asunaprevir	HCV Protease	73.1	79.4	59.5	105.9
1097	S4502	Eltrombopag	Thrombin	42.9	27.0	83.6	87.1
1098	S5009	Brivudine	Anti-infection	108.2	101.6	95.8	80.3

1099	S4516	(+)-Camphor	TRPV	120.0	107.1	118.9	92.3
1100	S5028	4-Aminopyridine	Potassium Channel	110.2	89.4	107.3	89.8
1101	S4528	Furazolidone	Anti-infection	101.1	84.2	65.4	66.8
1102	S5062	Daclatasvir Digydrochloride	HCV Protease	61.1	57.6	74.7	80.7
1103	S4541	Triclosan	Anti-infection	117.2	102.6	109.7	100.0
1104	S5100	Pralidoxime Iodide	AChR	111.5	102.8	112.0	92.6
1105	S4554	Bucetin	Others	115.7	102.6	106.1	85.5
1106	S7007	Binimetinib (MEK162, ARRY-162, ARRY-438162)	MEK	99.5	100.2	117.1	96.1
1107	S4565	Diiodohydroxyquinoline	Anti-infection	27.4	38.0	112.9	78.0
1108	S7158	abemaciclib (LY2835219)	CDK	17.2	12.8	57.5	58.1
1109	S4583	Butamben	Others	58.8	78.2	103.2	90.2
1110	S7284	Rociletinib (CO-1686, AVL-301)	EGFR	26.5	24.3	98.5	85.5
1111	S4595	Cefazolin Sodium	Anti-infection	100.3	90.0	98.5	96.2
1112	S7492	Uprosertib (GSK2141795)	Akt	18.0	13.9	54.5	56.7
1113	S4609	Diflunisal	COX	23.0	20.0	105.1	118.8
1114	S7633	Lomitapide Mesylate	Others	11.3	8.2	28.6	21.4
1115	S4625	Alcaftadine	Histamine Receptor	74.3	76.1	103.3	106.7
1116	S7772	Elacridar (GF120918)	P-gp	89.0	94.7	86.8	97.7
1117	S4634	Sodium sulfadiazine	Anti-infection	84.1	82.1	103.5	99.0
1118	S7810	Afatinib (BIBW2992) Dimaleate	EGFR, HER2	19.5	13.3	82.6	73.7
1119	S4646	Ciclesonide	Others	19.7	16.2	66.6	37.5
1120	S7910	Epacadostat (INCB024360)	IDO	93.7	106.1	103.1	77.1
1121	S4656	Parecoxib	COX	95.7	101.3	112.5	86.6
1122	S8022	Empagliflozin (BI 10773)	SGLT	114.5	101.7	102.1	84.9
1123	S4665	Rebeprazole sodium	Proton Pump	102.1	98.1	105.0	79.0
1124	S8116	Acalabrutinib (ACP-196)	BTK	98.8	95.0	100.7	79.8
1125	S4679	Terazosin HCl	Adrenergic Receptor	115.5	106.5	105.3	80.0
1126	S8241	Ponesimod	S1P Receptor	104.6	97.5	153.4	80.8
1127	S4693	Guanfacine Hydrochloride	Adrenergic Receptor	95.8	95.8	111.1	88.8

1128	S1717	Fomepizole	Dehydrogenase	108.0	106.9	115.4	93.1
1129	S4733	Retigabine	Potassium Channel	104.6	96.5	116.3	101.4
1130	S4521	DEET	Anti-infection	98.6	104.3	97.6	96.3
1131	S4816	Diatrizoate sodium	Others	86.6	92.7	108.3	91.0
1132	S4695	D panthenol	Vitamin	101.3	95.0	104.8	110.6
1133	S4836	Nilutamide	Androgen Receptor	59.6	58.8	102.9	87.9
1134	S4849	Levocetirizine Dihydrochloride	Histamine Receptor	101.5	102.5	99.2	98.2
1135	S4897	Nortropine Hydrochloride	Others	105.1	99.7	110.5	97.0
1136	S4975	Fimasartan	RAAS	93.3	85.3	100.8	89.3
1137	S4503	Calcium D-Panthotenate	Vitamin	84.3	94.9	65.0	60.7
1138	S5012	Octenidine Dihydrochloride	Anti-infection	11.8	9.2	26.9	20.2
1139	S4517	Cefotaxime sodium	Anti-infection	115.1	111.0	108.0	86.6
1140	S5030	Dihydralazine sulphate	Others	115.8	89.9	110.5	93.5
1141	S4529	Idramantone	Immunology & Inflammation related	115.1	106.3	108.9	87.2
1142	S5066	Pramipexole dihydrochloride	Dopamine Receptor	111.5	97.9	106.8	84.9
1143	S4542	Trihexyphenidyl hydrochloride	AChR	105.1	96.7	95.5	80.2
1144	S5206	Benzylpenicillin potassium	Anti-infection	118.3	105.6	112.3	88.6
1145	S4555	Carsalam	Anti-infection	118.9	104.8	109.7	89.5
1146	S7015	Birinapant	IAP	112.3	113.5	107.5	70.4
1147	S4566	DL-Panthenol	Others	121.2	110.9	116.6	100.4
1148	S7161	Motolimod (VTX-2337)	TLR	31.1	20.1	93.8	92.1
1149	S4584	Butylparaben	Anti-infection	98.0	88.3	111.4	98.8
1150	S7297	Osimertinib (AZD9291)	EGFR	11.1	8.9	28.6	21.9
1151	S4596	Cefixime	Anti-infection	99.2	96.5	104.4	111.8
1152	S7505	(S)-crizotinib	MTH1	21.2	14.8	71.1	56.1
1153	S4610	Mebendazole	Anti-infection	28.4	21.2	88.1	85.1
1154	S7634	Cerdulatinib (PRT062070, PRT2070)	JAK	20.6	14.5	84.1	58.3
1155	S4626	Ethosuximide	Calcium Channel	109.1	104.3	107.4	99.0
1156	S7775	Emricasan	Caspase	94.1	81.2	114.0	94.1

1157	S4635	Cyproheptadine hydrochloride	5-HT Receptor	38.4	46.3	92.0	89.3
1158	S7818	Pexidartinib (PLX3397)	CSF-1R,c-Kit	98.2	96.7	101.1	93.7
1159	S4647	Cefmenoxime hydrochloride	Anti-infection	107.2	104.0	101.7	88.9
1160	S7975	Favipiravir (T-705)	DNA/RNA Synthesis	105.8	101.4	103.0	87.9
1161	S4657	Eslicarbazepine Acetate	Others	101.7	111.8	112.3	94.3
1162	S8034	Apremilast (CC-10004)	TNF-alpha,PDE	105.5	94.8	111.8	85.5
1163	S4666	Sivelestat sodium tetrahydrate	Serine Protease	104.4	98.0	102.1	92.1
1164	S8134	Radotinib	Bcr-Abl	82.5	80.5	62.2	46.3
1165	S4680	Protirelin	Others	110.0	103.0	110.2	93.2
1166	S8266	Melphalan	DNA alkylator	67.3	49.9	117.0	82.4
1167	S4696	CarbinoxaMine Maleate	Histamine Receptor	120.9	104.6	121.2	88.8
1168	S1750	Octocrylene	Others	80.3	82.6	112.2	94.9
1169	S4734	Retigabine 2HCl	Potassium Channel	77.6	83.6	116.1	114.4
1170	S4570	Halothane	Others	110.5	107.6	109.7	97.3
1171	S4817	Atenolol	Adrenergic Receptor	106.2	97.4	105.0	106.0
1172	S4709	Latanoprost	Immunology & Inflammation related, AR/RXR, GPR	107.3	107.1	113.1	99.2
1173	S4837	Ibudilast	PDE	68.8	68.6	111.7	108.1
1174	S4852	Gadopentetate Dimeglumine	Others	116.5	116.7	113.4	101.3
1175	S4898	Sulfalozine sodium	Anti-infection	121.1	105.2	111.1	103.3
1176	S4976	Sulfalene(SMPZ)	Others	111.1	102.0	99.4	89.9
1177	S4504	6-Mercaptopurine (6-MP) Monohydrate	DNA/RNA Synthesis	37.7	30.4	97.5	86.4
1178	S5013	Ioversol	Others	15.5	10.3	112.3	85.5
1179	S4518	Chloroxylenol	Anti-infection	94.3	101.5	113.2	97.6
1180	S5032	Mephenesin	NMDAR	108.9	97.6	104.7	89.7
1181	S4530	i-Inositol	Others	103.2	96.2	107.6	89.2
1182	S5070	Mupirocin calcium	Anti-infection	95.1	105.6	57.9	49.4
1183	S4543	Trimetazidine dihydrochloride	Others	114.2	105.8	116.4	92.4

1184	S5208	Ciprofloxacin hydrochloride hydrate	Anti-infection	112.5	106.8	67.7	49.9
1185	S4556	Carzenide	Others	114.6	99.2	107.0	98.1
1186	S7028	Duvelisib (IPI-145, INK1197)	PI3K	30.5	32.8	99.5	83.4
1187	S4569	Fluphenazine dihydrochloride	Dopamine Receptor	11.8	9.3	115.0	48.2
1188	S7179	BAF312 (Siponimod)	S1P Receptor	74.5	83.8	42.2	100.6
1189	S4585	Succinylsulfathiazole	Anti-infection	117.7	103.7	111.1	97.1
1190	S7326	Tasisulam	Caspase	51.0	39.9	116.0	90.5
1191	S4597	Lercanidipine hydrochloride	Calcium Channel	26.5	11.9	71.3	84.6
1192	S7521	Afuresertib (GSK2110183)	Akt	16.8	12.1	59.4	57.1
1193	S4612	Dapson	Anti-infection	100.1	98.6	98.2	103.9
1194	S7635	Lomitapide	Others	12.6	9.1	27.9	22.4
1195	S4628	(+/-)-Sulfinpyrazone	COX	116.5	107.6	114.4	103.9
1196	S7781	Sunitinib	PDGFR,c-Kit,VEGFR	16.3	10.5	60.6	51.6
1197	S4636	Teneligliptin hydrobromide	DPP-4	93.5	103.0	115.3	101.0
1198	S7829	CB1954	DNA alkylator	90.1	81.9	103.2	100.6
1199	S4648	Dantrolene sodium hemiheptahydrate	Calcium Channel	17.8	13.7	96.5	91.6
1200	S7977	Napabucasin	STAT	13.4	16.0	29.8	22.3
1201	S4658	Hydroquinidine	Others	70.6	98.1	98.1	96.0
1202	S8041	Cobimetinib (GDC-0973, RG7420)	MEK	33.8	9.8	44.6	47.0
1203	S4667	Lidocaine hydrochloride	EGFR	95.2	94.8	112.6	86.2
1204	S8135	Riociguat (BAY 63-2521)	Others	106.4	96.9	105.7	77.7
1205	S4682	Loxoprofen	Immunology & Inflammation related	109.2	100.7	111.8	90.6
1206	S8294	Olmutinib (HM61713, BI 1482694)	EGFR, BTK	18.3	13.3	49.5	38.7
1207	S4699	Etretinate	Retinoid Receptor	73.1	76.4	113.7	98.6
1208	S2023	Propofol	GABA Receptor	97.6	97.4	106.2	92.2
1209	S4736	Trapidil	PDGFR	94.6	105.0	118.9	96.8

1210	S4578	Tyloxapol	Others	103.4	111.4	93.1	86.7
1211	S4819	Saccharin	Others	122.6	105.8	114.7	93.0
1212	S4851	Tafluprost	Others	107.7	104.8	117.4	102.6
1213	S4838	Acotiamide hydrochloride	AChR	93.4	80.6	94.1	89.2
1214	S4854	Bedaquiline fumarate	Microtubule Associated,AChR	40.8	60.7	83.1	99.5
1215	S4899	Sulfogaiacol	Others	116.4	109.3	102.9	105.7
1216	S5002	Fingolimod (FTY720) HCl	S1P Receptor	12.4	9.6	28.8	22.7
1217	S4505	Vinblastine sulfate	Microtubule Associated,AChR	13.6	10.6	90.2	89.2
1218	S5015	Simeprevir	HCV Protease	12.9	9.4	99.4	75.0
1219	S4519	Citric acid trilithium salt tetrahydrate	Others	54.2	87.4	106.8	95.3
1220	S5033	Terconazole	Anti-infection	26.0	11.0	74.8	74.3
1221	S4532	Iopamidol	Others	105.9	78.4	108.7	91.7
1222	S5074	Argatroban Monohydrate	Thrombin	103.5	90.0	95.9	103.8
1223	S4546	Xylitol	Others	93.7	88.8	101.8	86.5
1224	S5209	Enoxacin Sesquihydrate	Anti-infection	113.6	102.3	112.4	89.0
1225	S4558	Citiolone	Others	106.0	96.4	111.4	95.1
1226	S7077	Cilengitide?trifluoroacetate	Integrin	99.7	98.5	94.5	78.7
1227	S4571	Hexylresorcinol	Tyrosinase	102.1	90.9	121.6	101.3
1228	S7215	Losmapimod (GW856553X)	p38 MAPK	92.4	79.2	100.8	91.6
1229	S4588	Docusate Sodium	Opioid Receptor	102.6	97.0	98.8	96.0
1230	S7358	Poziotinib (HM781-36B)	HER2, EGFR	92.3	79.7	77.9	91.0
1231	S4601	Clioquinol	Anti-infection	65.6	79.4	43.7	59.4
1232	S7523	Entospletinib (GS-9973)	Syk	98.3	65.2	100.2	85.9
1233	S4617	Dextromethorphan hydrobromide hydrate	AChR	99.5	105.0	96.0	93.7
1234	S7645	Pilaralisib (XL147)	PI3K	78.7	62.1	90.3	80.0
1235	S4629	Chlorotrianisene	Estrogen/progestogen Receptor	72.4	76.3	97.1	90.5
1236	S7782	Dasatinib Monohydrate	Src,c-Kit,Bcr-Abl	36.3	20.4	87.8	78.4
1237	S4637	Prasugrel Hydrochloride	P2 Receptor	52.2	77.1	119.2	118.7
1238	S7852	Eliglustat	Others	70.7	71.3	100.0	84.7
1239	S4649	Atipamezole hydrochloride	Adrenergic Receptor	25.8	42.0	88.5	103.7
1240	S7995	Ripasudil (K-115) hydrochloride dihydrate	ROCK	13.3	9.3	91.5	94.1

1241	S4660	Glycopyrrolate	AChR	14.0	9.9	100.8	99.0
1242	S8048	Venetoclax (ABT-199, GDC-0199)	Bcl-2	13.4	9.6	60.9	39.3
1243	S4668	Procaine	Sodium Channel	75.3	47.5	102.8	88.2
1244	S8146	Mitomycin C	DNA/RNA Synthesis	12.4	9.1	31.8	25.6
1245	S4683	Sildenafil Mesylate	PDE	91.9	78.2	107.8	88.5
1246	S8432	Troglitazone (CS-045)	PPAR	105.3	96.7	120.4	80.4
1247	S4714	(-)-Menthol	Opioid Receptor	61.7	91.7	100.1	94.4
1248	S3669	Carmustine	DNA/RNA Synthesis	73.9	76.9	114.3	93.2
1249	S4748	Ondansetron Hydrochloride Dihydrate	5-HT Receptor	98.8	94.8	119.0	94.3
1250	S4581	Triacetin	Anti-infection	102.6	97.4	103.7	90.7
1251	S4830	Maltose	Others	107.8	94.8	116.3	100.3
1252	S7684	Salirasib	Rho	91.9	91.1	103.3	90.3
1253	S4839	Mosapride	5-HT Receptor	103.1	91.3	105.6	79.9
1254	S4869	Sulfamethoxazole sodium	Anti-infection	111.7	99.8	104.8	92.3
1255	S4927	Cyclen	Others	53.9	37.5	81.3	59.8
1256	S5003	Tacrolimus (FK506)	mTOR	71.2	63.0	101.8	103.6
1257	S4506	Acetazolamide	Carbonic Anhydrase	12.7	9.6	106.0	100.4
1258	S5017	Oxyclozanide	Anti-infection	14.0	9.3	115.4	59.9
1259	S4522	Dehydroacetic acid	Anti-infection	115.9	101.9	113.8	98.0
1260	S5039	Actarit	Immunology & Inflammation related	107.6	83.2	105.6	102.4
1261	S4535	Methylene Blue	Others	23.5	18.2	93.6	93.7
1262	S5075	Acotiamide	AChR	108.2	88.3	107.5	84.4
1263	S4548	Aminoguanidine hydrochloride	NOS	109.1	93.2	111.0	88.2
1264	S5210	Sulfamethazine Sodium Salt	Anti-infection	58.3	92.6	115.4	88.4
1265	S4559	Cloxiquine	Anti-infection	23.8	11.6	61.2	67.6
1266	S7083	Ceritinib (LDK378)	ALK	11.0	9.7	27.6	20.8
1267	S4574	Piperazine	GABA Receptor	106.3	105.3	112.6	103.6
1268	S7218	Alvelestat (AZD9668)	Serine Protease	113.1	96.1	95.1	81.4
1269	S4589	Amodiaquine dihydrochloride dihydrate	Transferase,Histone Methyltransferase	58.7	58.8	98.9	83.8

1270	S7393	Aloxistatin	Cysteine Protease	109.9	101.1	112.2	92.1
1271	S4602	Acetohydroxamic acid	Anti-infection	95.9	79.8	104.2	93.4
1272	S7550	Erythromycin Cyclocarbonate	Anti-infection	103.1	103.6	93.2	81.6
1273	S4618	Fenoldopam mesylate	Dopamine Receptor	88.0	73.4	161.4	128.5
1274	S7646	Voxtalisib (XL765, SAR245409)	mTOR, PI3K	19.2	13.0	86.6	84.7
1275	S4630	Diazoxide	Potassium Channel	90.1	90.8	106.5	94.8
1276	S7784	Fumagillin	Anti-infection	58.5	36.8	96.4	99.0
1277	S4638	Desogestrel	Estrogen/progestogen Receptor	27.6	33.3	113.5	128.5
1278	S7854	Ulixertinib (BVD-523, VRT752271)	ERK	72.5	57.8	143.7	117.1
1279	S4650	Atipamezole	Adrenergic Receptor	14.1	9.9	108.1	96.0
1280	S7998	Entrectinib (RXDX-101)	Trk receptor,ALK	29.7	25.8	32.3	21.4
1281	S4661	Tiagabine hydrochloride	GABA Receptor	81.3	86.3	107.5	91.9
1282	S8051	Macitentan	Endothelin Receptor	99.6	78.4	104.9	96.9
1283	S4669	Benzocaine hydrochloride	Sodium Channel	86.7	70.0	111.3	95.5
1284	S8183	Pimavanserin	5-HT Receptor	11.9	9.0	30.5	21.4
1285	S4685	Efavirenz	Reverse Transcriptase	90.4	91.4	88.1	76.8
1286	S8558	Tofogliflozin (CSG 452)	SGLT	76.2	91.5	110.6	90.9
1287	S4718	Acetylcholine iodide	AChR	45.3	87.9	107.9	92.1
1288	S3681	Vitamin E Acetate	Vitamin	110.7	104.8	108.0	96.7
1289	S4750	Sulfacetamide sodium salt hydrate	Autophagy,Anti-infection	99.7	105.2	112.0	98.8
1290	S4599	Benzyl benzoate	Others	107.6	89.8	106.7	90.1
1291	S4831	Piperonyl butoxide	Others	101.1	93.7	111.2	101.5
1292	S4843	Potassium acetate	Anti-infection	106.1	97.8	106.6	93.4
1293	S4877	Eperisone hydrochloride	Others	22.5	15.8	101.4	96.3
1294	S4928	Brivaracetam	Others	108.2	100.9	115.2	106.5
1295	S5004	Pimecrolimus	Immunology & Inflammation related	56.7	64.1	104.3	113.1
1296	S4507	17-Hydroxyprogesterone	Estrogen/progestogen Receptor	57.0	85.0	98.2	109.6
1297	S5023	Nadolol	Androgen Receptor	100.1	99.2	107.1	90.4
1298	S4525	Ethylparaben	Anti-infection	88.3	90.1	110.1	91.6
1299	S5042	Bevantolol hydrochloride	Adrenergic Receptor	99.1	72.5	104.0	93.0

1300	S4536	Nitrofurantoin	Anti-infection	95.4	80.6	62.8	59.4
1301	S5077	Regorafenib Monohydrate	VEGFR,PDGFR,c-Kit,c-RET,Raf	17.2	12.2	102.6	82.9
1302	S4550	Azelaic acid	Anti-infection	84.5	81.0	102.1	91.4
1303	S5305	Bromisoval	Others	103.8	93.5	122.8	91.8
1304	S4561	Danthron	Others	53.6	70.3	121.5	97.3
1305	S7155	Batimastat (BB-94)	MMP	40.8	47.7	100.1	89.0
1306	S4576	Sulfabenzamide	Anti-infection	96.1	82.9	115.6	100.7
1307	S7252	Selinexor (KPT-330)	CRM1	23.8	16.7	86.3	88.8
1308	S4591	Nitroxoline	Topoisomerase	17.2	11.9	46.9	44.8
1309	S7397	Sorafenib	Raf	19.6	21.3	101.8	97.3
1310	S4603	Gallic acid	ROS	31.8	21.8	109.8	91.4
1311	S7579	Ledipasvir (GS5885)	HCV Protease	52.5	46.5	75.1	61.9
1312	S4619	Itopride hydrochloride	AChR	105.5	97.9	112.5	92.5
1313	S7660	Obeticholic Acid	FXR	101.1	97.9	107.3	104.2
1314	S4631	Prochlorperazine dimaleate salt	Dopamine Receptor	19.3	14.2	49.6	28.5
1315	S7786	Erlotinib	EGFR	61.1	46.7	104.1	87.8
1316	S4639	Brexpiprazole	5-HT Receptor,Dopamine Receptor	103.2	92.2	103.3	102.0
1317	S7856	Tenofovir Alafenamide (GS-7340)	Reverse Transcriptase	63.5	56.0	106.3	95.8
1318	S4651	Etoricoxib	COX	14.2	9.5	113.1	102.7
1319	S8001	Ricolinostat (ACY-1215)	HDAC	12.8	9.8	100.4	80.8
1320	S4662	Atazanavir	HIV Protease	97.5	94.8	109.3	107.1
1321	S8057	Pacritinib (SB1518)	FLT3,JAK	12.5	9.4	39.2	37.7
1322	S4673	Etonogestrel	Estrogen/progestogen Receptor	67.0	71.0	95.0	84.0
1323	S8195	Oclacitinib?maleate	JAK	93.5	77.9	99.4	85.3
1324	S4689	Deoxycholic acid	Others	94.6	79.0	105.8	99.4
1325	S8565	Omarigliptin (MK-3102)	DPP-4	104.4	87.0	115.3	89.9
1326	S4727	Cinnarizine	Calcium Channel	95.8	91.7	113.5	88.7
1327	S3738	Travoprost	Others	61.3	68.5	125.3	96.2
1328	S4751	Cisapride hydrate	5-HT Receptor	68.9	62.4	112.2	86.2
1329	S4675	Tiagabine	GABA Receptor	90.6	87.0	98.7	83.8

1330	S4832	Tolmetin	Immunology & Inflammation related	90.8	85.1	97.5	85.1
1331	S4844	Cefcapene Pivoxil Hydrochloride	Anti-infection	101.5	89.1	103.4	89.1
1332	S4878	Neticonazole Hydrochloride	Anti-infection	30.0	39.1	96.2	80.9
1333	S4931	Propiverine hydrochloride	Others	64.0	71.4	117.7	97.5
1334	S5005	Cefotiam hydrochloride	Anti-infection	33.8	19.4	108.7	102.1
1335	S4512	Aceglutamide	Others	16.8	10.2	106.0	107.5
1336	S5025	Efinaconazole	Anti-infection	56.6	67.5	105.8	106.5
1337	S4526	Fenbufen	COX	112.0	93.9	116.8	92.6
1338	S5052	Granisetron	5-HT Receptor	104.7	72.6	100.8	91.8
1339	S4538	Pantoprazole sodium	Proton Pump	96.3	81.6	109.7	94.3
1340	S5079	Sitagliptin	DPP-4	103.1	84.3	117.9	96.3
1341	S4552	Bithionol	cAMP	100.7	95.6	102.0	60.9
1342	S5309	Propyphenazone (4-Isopropylantipyrine)	Others	107.9	89.7	119.2	96.8
1343	S4562	Dehydrocholic acid	Others	106.2	88.0	119.4	104.9
1344	S7156	Marimastat (BB-2516)	MMP	48.7	50.6	109.2	86.7
1345	S4577	Terpin hydrate	Others	106.5	95.5	117.8	122.4
1346	S7262	Vidofludimus	Dehydrogenase	25.5	17.4	95.4	99.8
1347	S4593	Chlormadinone acetate	Androgen Receptor,5-alpha Reductase	53.9	59.2	105.9	108.7
1348	S7417	Puromycin 2HCl	Anti-infection	12.2	9.0	32.9	26.7
1349	S4604	Levofloxacin hydrate	Topoisomerase	62.9	62.3	71.7	55.7
1350	S7605	Filgotinib (GLPG0634)	JAK	38.5	30.0	104.3	98.1
1351	S4620	Cefuroxime sodium	Anti-infection	105.6	93.3	120.7	107.4
1352	S7678	Sacubitril/valsartan (LCZ696)	RAAS	27.1	15.5	111.2	103.0
1353	S4632	Hexachlorophene	Potassium Channel	26.7	18.6	155.6	148.8
1354	S7787	Docetaxel Trihydrate	Microtubule Associated	33.0	20.0	111.0	97.9
1355	S4640	Lesinurad	Others	32.0	19.7	109.6	104.1
1356	S7858	Dibutyryl-cAMP (Bucladesine)	PKA	35.4	22.5	114.6	111.5
1357	S4652	Sulisobenzone	Others	89.9	91.2	107.2	109.7

1358	S8016	Vonoprazan Fumarate (TAK-438)	Potassium Channel	91.0	85.2	109.2	143.3
1359	S4663	Fusidate Sodium	Anti-infection	91.9	90.7	65.2	55.4
1360	S8067	Vorapaxar	Estrogen/progestogen Receptor	77.1	76.7	110.0	100.3
1361	S4674	Hydroxyprogesterone caproate	Estrogen/progestogen Receptor	37.7	50.4	87.8	110.8
1362	S8205	Enasidenib (AG-221)	Dehydrogenase	106.7	73.3	99.3	72.5
1363	S4690	Escin	Immunology & Inflammation related	91.9	97.9	105.6	117.2
1364	S8594	Tirofiban Hydrochloride	Integrin	93.9	85.8	255.3	110.8
1365	S4729	Tamsulosin hydrochloride	Adrenergic Receptor	92.9	93.0	114.7	140.7
1366	S3756	Methyl salicylate	TRPV	112.0	99.6	111.8	110.3
1367	S4813	Cefuroxime axetil	Anti-infection	109.6	101.0	122.6	152.6
1368	S4686	Vitamin E	COX,VEGFR	116.7	99.4	112.0	96.0
1369	S4834	Propantheline bromide	AChR	101.9	95.5	110.0	98.7
1370	S4847	Faropenem Sodium	Anti-infection	106.1	91.5	109.2	105.9
1371	S4887	Nikethamide	Others	113.2	98.2	112.7	115.3
1372	S4932	Proxyphylline	Adenosine Receptor	35.8	21.9	112.7	113.9
1373	S5006	Teprenone	Others	39.0	23.0	106.1	118.4
1374	S1046	Vandetanib	VEGFR	36.9	27.1	44.8	30.8
