# Supplementary material for: Nintedanib inhibits the VEGFR–ERK signaling pathway in human KRAS-mutated cancer cells
Source: Cell Death Dis. 2026 Jun 23;17(1):780. doi: 10.1038/s41419-026-09015-2 (PMC13547290; doi:10.1038/s41419-026-09015-2)
Supplement: Supplementary file 5 [file 41419_2026_9015_MOESM5_ESM.pdf]

Table S4. Effect of 22 VEGFR inhibitors on the proliferation of *KRAS*^{G12D} and *KRAS*^{WT} cells.

No	Product Name	Target	Sachi (% cells)	Sachi^{G12A} (% cells)	HPNE (% cells)	HPNE^{G12D} (% cells)
1	Axitinib	c-Kit, PDGFR, VEGFR	55.0	28.7	91.0	72.1
2	Nintedanib	FGFR, PDGFR, VEGFR	91.4	53.2	46.4	21.3
3	Cediranib	VEGFR	45.6	16.4	73.5	56.8
4	Pazopanib HCl	c-Kit, PDGFR, VEGFR	71.9	90.4	85.7	60.0
5	Sorafenib Tosylate	PDGFR, Raf, VEGFR	105.8	108.8	107.5	80.5
6	Sunitinib Malate	c-Kit, PDGFR, VEGFR	14.5	11.0	53.9	37.5
7	Vatalanib	VEGFR	32.8	29.7	75.2	61.0
8	Foretinib	c-Met, VEGFR	23.7	23.5	33.9	18.8
9	Cabozantinib	c-Met, VEGFR	29.6	20.0	102.9	93.9
10	Lenvatinib	VEGFR	24.6	18.5	104.9	80.2
11	Regorafenib	c-RET, VEGFR	27.1	19.8	91.4	67.0
12	Tivozanib	c-Kit, PDGFR, VEGFR	36.0	58.1	76.1	70.9
13	Ponatinib	BcrAbl, FGFR, PDGFR, VEGFR	20.3	15.2	44.5	37.6
14	Pazopanib	c-Kit, PDGFR, VEGFR	107.2	104.3	87.3	95.2
15	Cabozantinib malate	TAM Receptor, VEGFR	34.0	22.4	102.9	93.9
16	Regorafenib Monohydrate	VEGFR, PDGFR, c-Kit, c-RET, Raf	17.2	12.2	102.6	82.9
17	Dovitinib	FLT3, cKit, FGFR, PDGFR, VEGFR	58.1	41.5	110.3	111.0
18	Sunitinib	PDGFR, c-Kit, VEGFR	16.3	10.5	60.6	51.6
19	Vitamin E	COX, VEGFR	116.7	99.4	112.0	96.0
20	Vandetanib	VEGFR	36.9	27.1	44.8	30.8
21	Linifanib	CSF-1R, PDGFR, VEGFR	95.2	101.9	99.4	86.8
