# Supplementary material for: Nintedanib inhibits the VEGFR–ERK signaling pathway in human KRAS-mutated cancer cells
Source: Cell Death Dis. 2026 Jun 23;17(1):780. doi: 10.1038/s41419-026-09015-2 (PMC13547290; doi:10.1038/s41419-026-09015-2)
Supplement: Supplementary file 6 [file 41419_2026_9015_MOESM6_ESM.pdf]

Table S5. IC50 values of cell lines used in this study

Cell line	Cell type	*KRAS* gene status	IC$_{50}$ (μM) Nintedanib	Axitinib	Motesanib
HPNE	pancreatic normal ductal epithelium	WT	15.9	20>	
HPNE/*KRAS*$^{\mathrm{G12D}}$		G12D	3.1	20>	20>
HBEC	bronchial normal epithelium	WT	20>	20>	20>
HBEC/*KRAS*$^{\mathrm{G12D}}$		G12D	2.8	20>	20>
Sachi	MM	WT	20>	20>	20>
Sachi/*KRAS*$^{\mathrm{G12A}}$	MM	G12A	2.7	20>	20>
A549	alveolar basal epithelial carcinoma	G12D	3.0	7.2	20>
Lu-99A	giant cell carcinoma	G12C	2.5	6.4	19.5
DLD1	CRC	G13D	6.8	7.9	18.0
SW-480	CRC	G12V	4.1	20>	20>
HCT116	CRC	G13D	2.3	4.9	20>
PL5	PDAC	G12D	3.1	18.1	20>
PL8	PDAC	G12D	2.9	12.1	20>
AsPc-1	PDAC	G12D	4.3	20>	20>

*CRC, colorectal PDAC, pancreatic ductal carcinoma; MM, multiple myeloma

Analysis: Origin 2021b
