# Supplementary material for: Nintedanib inhibits the VEGFR–ERK signaling pathway in human KRAS-mutated cancer cells
Source: Cell Death Dis. 2026 Jun 23;17(1):780. doi: 10.1038/s41419-026-09015-2 (PMC13547290; doi:10.1038/s41419-026-09015-2)
Supplement: Supplementary file 7 [file 41419_2026_9015_MOESM7_ESM.pdf]

Table S6. Primer sets used in this study

Symbol	primer (5′→3′)
KRAS epegRNA(tevo) PBS RT G12D F	gtgcTGCCTACGCCATCAGCTCCAACTACCACAA
KRAS epegRNA(tevo) PBS RT G12D R	cgcgTTGTGGTAGTTGGAGCTGATGGCGTAGGCA
pegRNA scaffold F	TAAAATAAGGCTAGTCCGTTATCAACTTGAAAAAGTGGCACCGAGTCG
pegRNA scaffold R	GCACCGACTCGGTGCCACTTTTTCAAGTTGATAACGGACTAGCCTTAT
KRAS pegRNA-1 sgRNA F	CACCGAAACTTGTGGTAGTTGGAGCGTTTTAGAGCTAGAAATAGCAAGT
KRAS pegRNA-1 sgRNA R	TTTAACTTGCTATTTCTAGCTCTAAAACGCTCCAACTACCACAAGTTTC
