# Supplementary material for: Nintedanib inhibits the VEGFR–ERK signaling pathway in human KRAS-mutated cancer cells
Source: Cell Death Dis. 2026 Jun 23;17(1):780. doi: 10.1038/s41419-026-09015-2 (PMC13547290; doi:10.1038/s41419-026-09015-2)
Supplement: Supplementary file 8 [file 41419_2026_9015_MOESM8_ESM.pdf]

Table S7. Antibodies used in this study

Molecule	Cat number	Company	Species	Dilution
p-VEGFR1	AF4170	R&D	Rb	×1000
VEGFR1	A19132	ABclonal	Rb	×1000
P-VEGFR2	AP0382	ABclonal	Rb	×1000
VEGFR2	A5609	ABclonal	Rb	×1000
p-ERK1/2	#9101	CST	Rb	×1000
ERK	4695	CST	Rb	×1000
p-AKT	#4060	CST	Rb	×1000
AKT	9272	CST	Rb	×1000
p-DRP1	#3455	CST	Rb	×1000
DRP1	12957-1-AP	Proteintech	Rb	x 1000
C-caspas3	#9664	CST	Rb	×1000
SOS1	BT-AP14373	BT LAP	Rb	×1000
KRAS	05-516	MERCK	M	×1000
Bcl2	#2872	CST	Rb	×1000
Bax	#2772	CST	Rb	×1000
VEGFR2	#2479	CST	Rb	×50
p53	M7001	Agilent	M	×150
GAPDH	#2118	CST	Rb	x 3000
Anti-rabbit IgG-HRP	#7074	CST	Goat	x 4000
Hoechst 33342	SW150	Dojindo		x 1000
Alexa Fluor Plus 488	A32723	Invitrogen		x 1000
Alexa Fluor® 568	A11011	Invitrogen		x 1000
Mitotracker®	M7512	Invitrogen		x 10000

CST, Cell Signaling Technology; Rb, rabbit; M, mouse
