# Supplementary material for: Nintedanib inhibits the VEGFR–ERK signaling pathway in human KRAS-mutated cancer cells
Source: Cell Death Dis. 2026 Jun 23;17(1):780. doi: 10.1038/s41419-026-09015-2 (PMC13547290; doi:10.1038/s41419-026-09015-2)
Supplement: Supplementary file 9 [file 41419_2026_9015_MOESM9_ESM.pdf]

Supplementary information

All the full and uncropped version of the western blot images used in manuscript

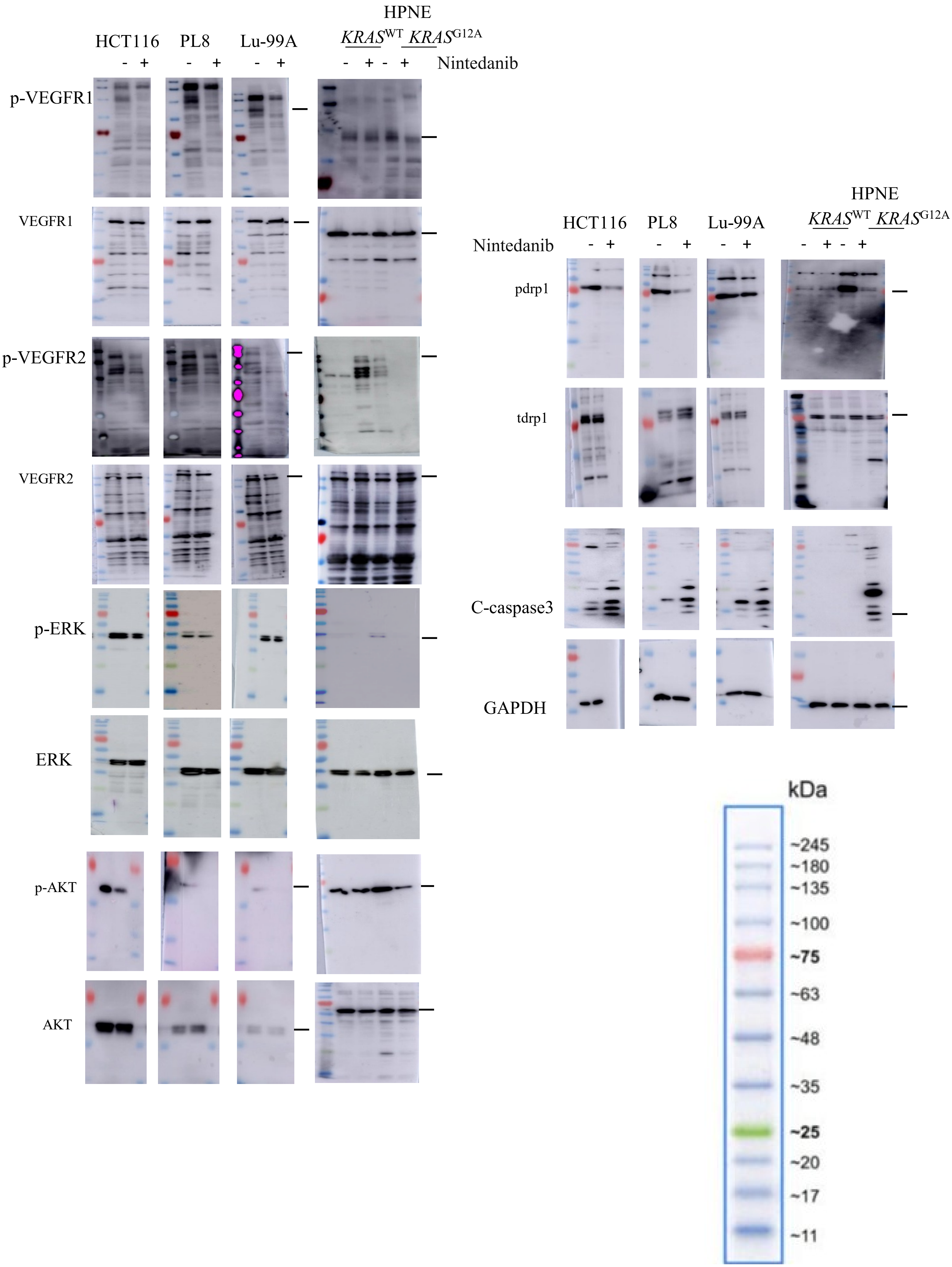

Fig. 3. Complete and unaltered Western blot image corresponding to Figure 3B

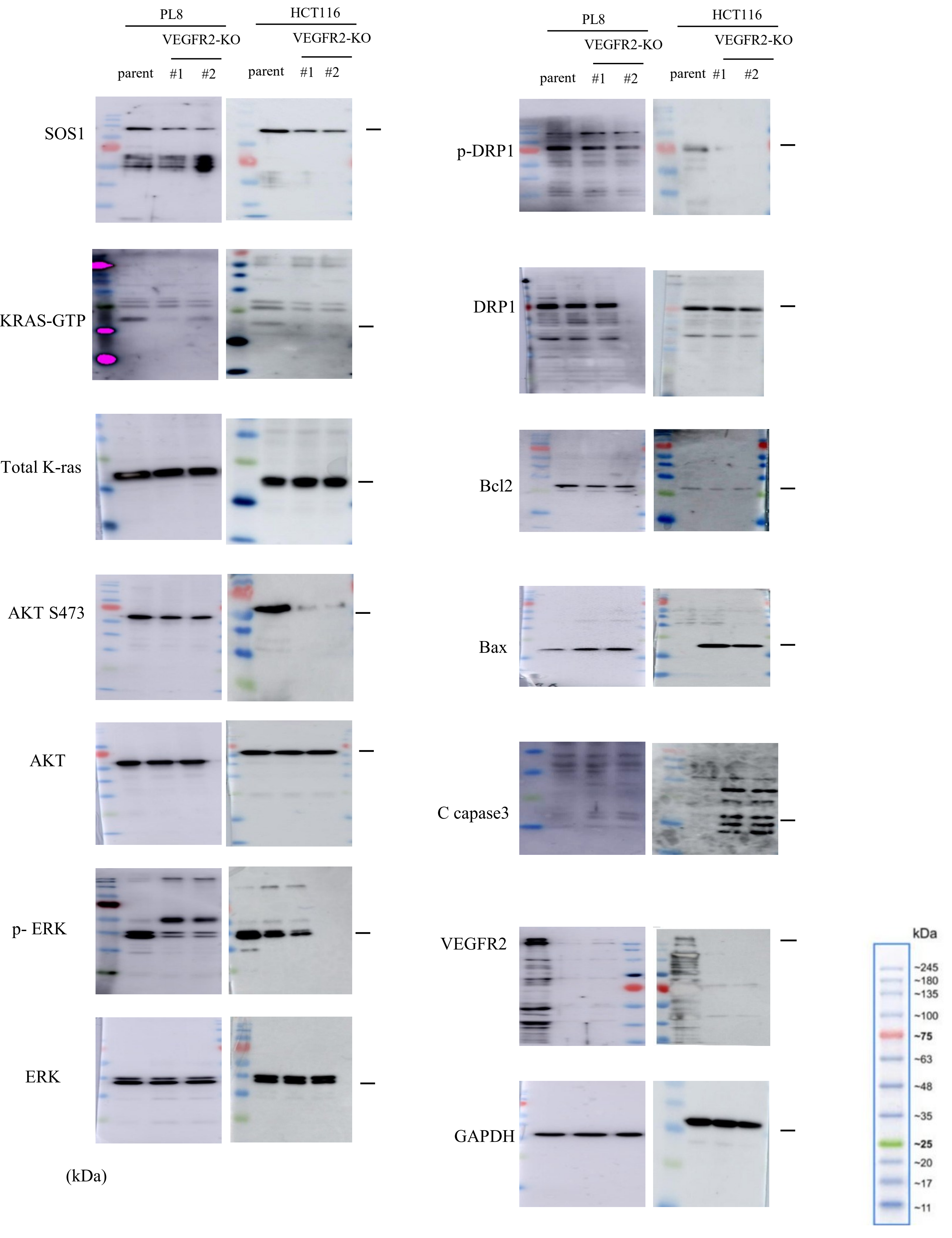

Fig. 4. Complete and unaltered Western blot image corresponding to Figure 4C

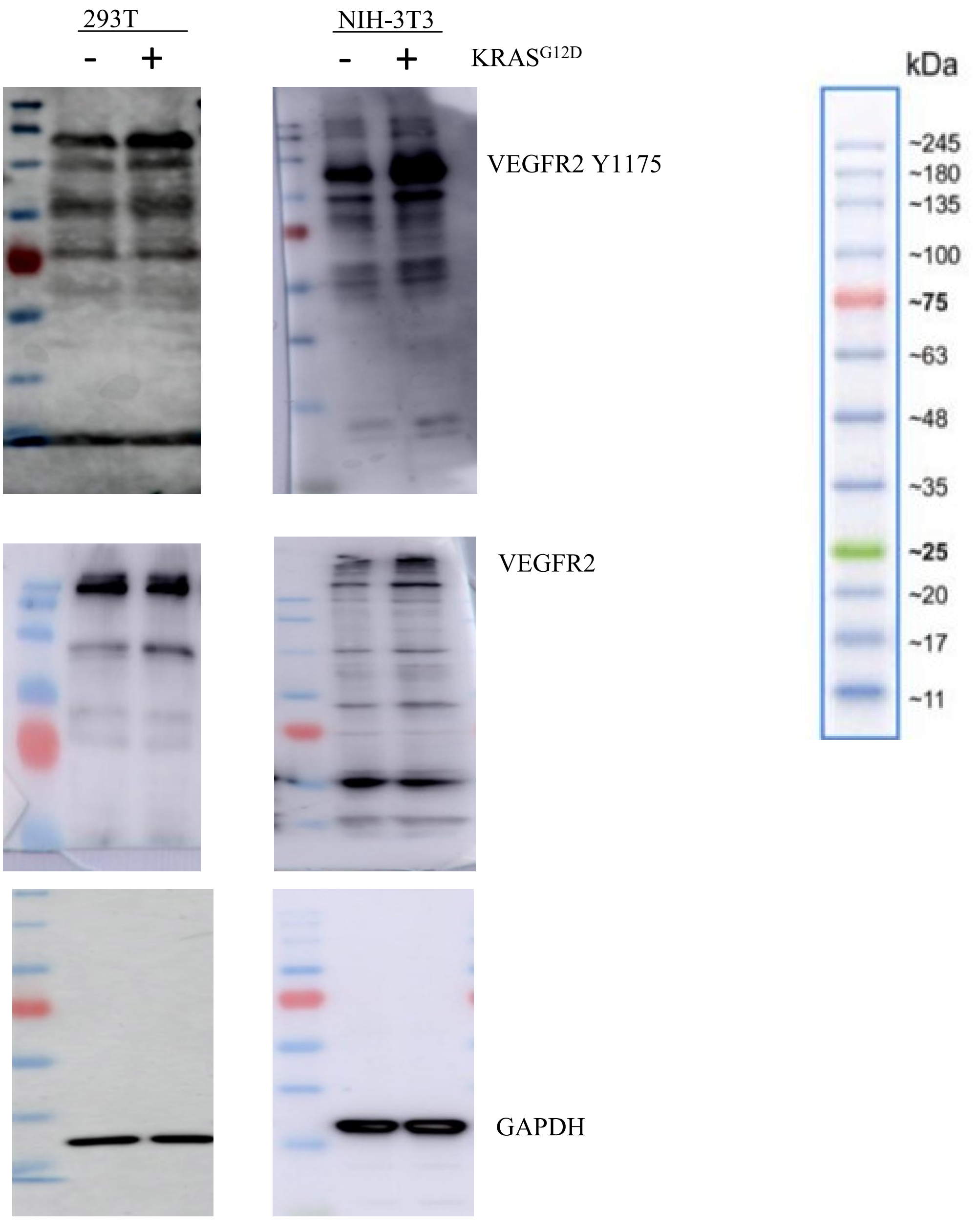

Fig. 4. Complete and unaltered Western blot image corresponding to Figure 4F

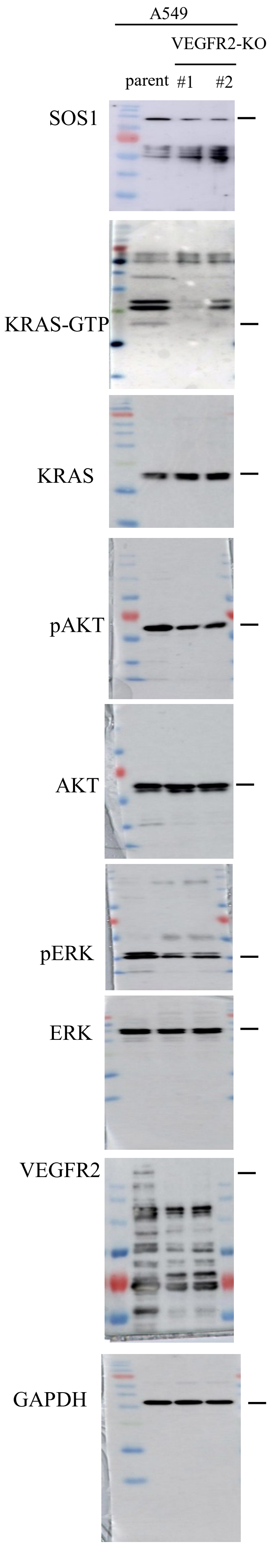

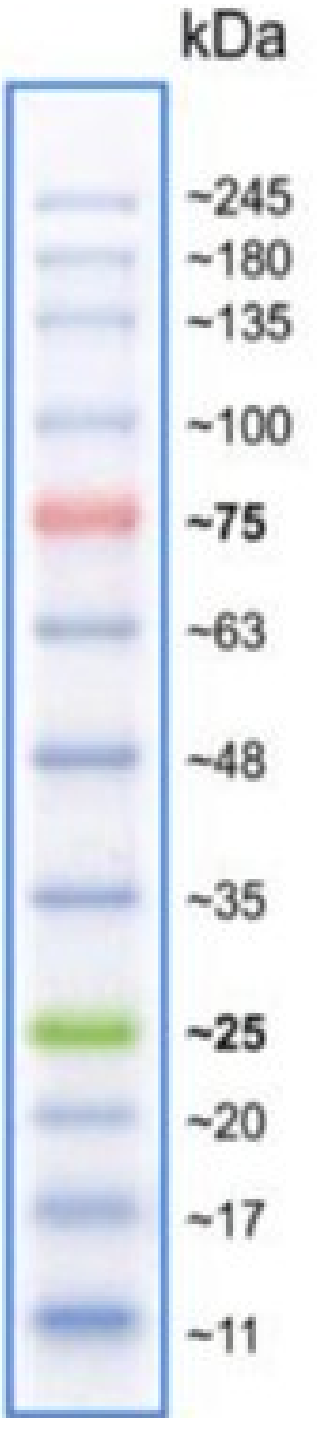

Fig. S5 Complete and unaltered Western blot image corresponding to Figure Fig.S5C
